# Synthesis and biological activity of 2-cyanoacrylamide derivatives tethered to imidazopyridine as TAK1 inhibitors

**DOI:** 10.1080/14756366.2020.1833876

**Published:** 2020-10-22

**Authors:** Seok Jong Kang, Jung Wuk Lee, Jiho Song, Jiwon Park, Jaeyul Choi, Kwee Hyun Suh, Kyung Hoon Min

**Affiliations:** a College of Pharmacy, Chung-Ang University, Seoul, Republic of Korea; b Hanmi Research Center, Hanmi Pharm. Co. Ltd., Gyeonggi-Do, Republic of Korea

**Keywords:** TAK1, imidazopyridine, 2-cyanoacrylamide, reversible covalent chemistry, small molecule inhibitor

## Abstract

The importance of transforming growth factor beta-activated kinase 1 (TAK1) to cell survival has been demonstrated in many studies. TAK1 regulates signalling cascades, the NF-κB pathway and the mitogen-activated protein kinase (MAPK) pathway. TAK1 inhibitors can induce the apoptosis of cancerous cells, and irreversible inhibitors such as (5Z)-7-oxozeaenol are highly potent. However, they can react non-specifically with cysteine residues in proteins, which may have serious adverse effects. Reversible covalent inhibitors have been suggested as alternatives. We synthesised imidazopyridine derivatives as novel TAK1 inhibitors, which have 2-cyanoacrylamide moiety that can form reversible covalent bonding. A derivative with 2-cyano-3-(6-methylpyridin-2-yl)acrylamide (**13h**) exhibited potent TAK1 inhibitory activity with an IC_50_ of 27 nM. It showed a reversible reaction with β-mercaptoethanol, which supports its potential as a reversible covalent inhibitor.

## Introduction

Transforming growth factor beta-activated kinase 1 (TAK1), also known as mitogen-activated protein kinase kinase kinase 7 (MAP3K7) or MEK kinase 7 (MEKK7), is a serine/threonine kinase encoded by *MAP3K7* gene. Since it was first found to be activated by transforming growth factor beta (TGFβ) and bone morphologic protein (BMP)^1^, TAK1 has been reported to mediate signal transduction for the regulation of cell proliferation and apoptosis pathways^2^. TAK1 is activated by various exogenous stimuli, including interleukin-1 (IL-1), lipopolysaccharide (LPS), tumour necrosis factor alpha (TNFα), and TGFβ^3^^,^^4^. TNFα has critical roles in signalling pathways for both cell survival and death^5–7^.

**Figure 1.**
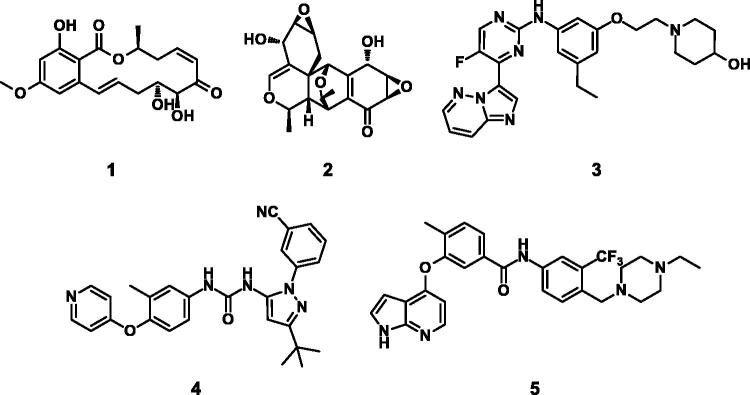
Structures of previously reported TAK1 inhibitors.

Because TAK1 is a key signalling element that is required for cell survival and death in TNF α signalling, it has emerged as a potential therapeutic target for cancer and inflammatory disease^8–10^. In TNFα stimulated breast cancer cells, inhibition of TAK1 causes apoptosis by switching from NFκB pro-survival signalling to induction of effector caspases^11^. *In-vivo* studies have provided evidence of a strong relationship between TAK1 and various malignancies, including pancreatic cancer^12^, colon cancer^13^, and breast cancer^14^. A number of small molecules have been reported to inhibit TAK1 (Figure 1). (5Z)-7-Oxozeaenol (5Z7O, **1**)^15^ and epoxyquinol B (EPQB, **2**)^16^ are compounds derived from fungi which possess a resorcylic lactone and an epoxide, respectively. Imidazo[1,2-*b*]pyridazine (**3**) was developed as a reversible type I inhibitor. It fits into an active DFG-in conformation with TAK1^17^. Pyrazole urea (**4**)^18^ and 1*H*-pyrrolo[2,3-*b*]pyridine (**5**)^19^ have been reported as Type II inhibitors which bind to TAK1 in the inactive DFG-out confirmation.

5Z7O is a potent irreversible TAK1 inhibitor^15^, although it is also a promiscuous kinase inhibitor. The *cis*-enone of 5Z7O has off-target effects because it forms covalent bonds with reactive cysteine residues^20^. Acrylamide Michael acceptors irreversibly bind to nucleophiles such as cysteine under physiological conditions^21^. Michael acceptors that undergo dual activation by electron-withdrawing groups form reversible covalent bonds by increasing the α-carbon acidity of covalent adducts^22^. Converting an irreversible warhead to a reversible warhead can limit off-target binding and increase the probability of binding the target site^23^.

**Figure 2.**
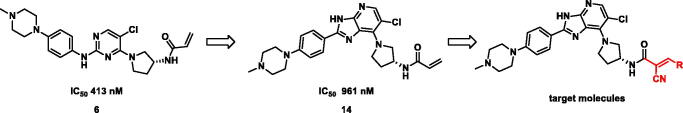
Schematic illustration of target molecule design.

In this study, we designed imidazopyridines with 2-cyanoacrylamide moiety for reversible covalent TAK1 inhibition. The screening of our in-house chemical library led to identification of a pyrimidine compound (**6**) with an IC_50_ of 413 nM against TAK1. To identify novel scaffold as TAK1 inhibitors, imidazopyridine scaffold was designed based on a bioisosteric replacement strategy. The imidazopyridine **14** showed retained activity although its IC_50_ was approximately twice that of the pyrimidine (**6**). Target molecules were designed for reversible covalent chemistry by replacing the acrylamide moiety with various 2-cyanoacrylamide moieties (Figure 2).

## Materials and methods

### Chemistry

Unless otherwise noted, all reagents and solvents were purchased from a commercial vendor and used without further purification. Reactions were monitored via thin-layer chromatography (TLC) using Merck TLC silica gel 60 F_254_ 250 µm plates. Flash column chromatography was performed using ZEOprep 60 silica gel (Zeochem, 40–63 µm) and a CombiFlash system (Teledyne ISCO) loaded with pre-packed silica gel flash column cartridges (Welux™). ^1^H and ^13^C NMR spectra were obtained using a Resonance ECZ 600R NMR spectrometer (JEOL). ^1^H NMR spectra were collected at 600 MHz, and ^13^C spectra were collected at 150 MHz using tetramethylsilane (TMS) as an internal standard. Chemical shifts are reported in parts per million (ppm, *δ*) downfield of TMS, and the coupling constant (*J*) is reported in hertz (Hz). Splitting patterns are reported with the following abbreviations: s, singlet; d, doublet; t, triplet; q, quartette; p, pentet; dd, doublet of doublets; dt, doublet of triplets; td, triplet of doublets; m, multiplet; br, broad signal. High-resolution mass spectrometry (HRMS) was performed using a Q-Exactive MS (ThermoScientific) coupled with an Ultimate 3000 LC system (Dionex). A ThermoScientific Hypersil GOLD C18 column (2.1 mm × 50 mm, 1.9 µm) was used for separation.

### (R)-tert-butyl-(1-(2-amino-5-chloro-3-nitropyridin-4-yl)pyrrolidin-3-yl)carbamate (8)

2-Amino-4,5-dichloro-3-nitropyridine (208 mg, 1 mmol), (*R*)-3-(Boc-amino)pyrrolidine (186 mg, 1 mmol) and K_2_CO_3_ (276 mg, 2 mmol) were dissolved in MeCN (3 ml) and stirred at room temperature (RT) for 8 h. Excess water was added to the reaction mixture, and the mixture was stirred at RT for an additional 17 h. The reaction mixture was then filtered, and the filter cake was washed with water to obtain **8** (191 mg, 53%). ^1^H-NMR (600 MHz, DMSO-d_6_) *δ* 7.87 (s, 1H), 7.21 (d, *J =* 5.5 Hz, 1H), 6.88 (s, 2H), 4.02 (d, *J =* 5.5 Hz, 1H), 3.64 (m, 1H), 3.53 (m, 2H), 3.23 (m, 1H), 2.05 (m, 1H), 1.83 (m, 1H), 1.38 (s, 9H). ^13^C-NMR (150 MHz, DMSO-d_6_) *δ* 155.3, 152.7, 151.5, 146.0, 123.2, 107.7, 78.0, 56.2, 49.6, 49.3, 30.4, 28.2. HRMS (ESI) [M + H]^+^: *m*/*z* calcd. 358.1277. Found 358.1274.

### (R)-tert-butyl (1-(2,3-diamino-5-chloropyridin-4-yl)pyrrolidin-3-yl)carbamate (9)

Compound **8** (179 mg, 0.5 mmol) and Fe powder (84 mg, 1.5 mmol) were dissolved in acetic acid (3 ml), and the mixture was stirred at 40 °C for 1 h. Saturated NaHCO_3_ was carefully added to the reaction mixture at 0 °C, and the mixture was extracted three times with ethyl acetate (EA). The organic layer was washed with brine, dried with Na_2_SO_4_, filtered and concentrated on a rotary evaporator. The concentrated mixture was purified via medium pressure liquid chromatography (MPLC) to obtain **9** (108 mg, 66%). ^1^H-NMR (300 MHz, CDCl_3_) *δ* 7.58 (s, 1H), 5.03 (m, 1H), 4.80 (m, 1H), 4.38 (m, 1H), 4.17 (bs, 2H), 3.85 (bs, 2H), 3.56 (m, 1H), 3.34 (m, 1H), 3.05 (m, 1H), 2.40 (m, 1H), 1.89 (m, 1H), 1.47 (s, 9H).

### (R)-tert-butyl-(1-(6-chloro-2-(4-(4-methylpiperazin-1-yl)phenyl)-3H-imidazo[4,5-b]pyridin-7-yl)pyrrolidin-3-yl)carbamate (10)

Compound **9** (75 mg, 0.22 mmol), 4-(4-methylpiperazin-1-yl)benzaldehyde (45 mg, 0.22 mmol) and FeCl_3_ (1 mg, 0.007 mmol) were dissolved in dimethylformamide (DMF, 1 ml), and the mixture was stirred at 120 °C for 16 h. Water was added to the reaction mixture, and the mixture was extracted with dichloromethane (DCM) three times. The extract was washed with brine, dried with Na_2_SO_4_, filtered and concentrated on a rotary evaporator. The concentrated mixture was purified via MPLC to obtain **10** (38 mg, 32%). ^1^H-NMR (600 MHz, CDCl_3_) *δ* 8.00–7.96 (m, 3H), 7.01 (d, *J =* 8.3 Hz, 2H), 5.56 (s, 1H), 4.34–4.15 (m, 5H), 3.35 (s, 4H), 2.61 (s, 4H), 2.38 (s, 3H), 2.22 (t, *J =* 6.2 Hz, 1H), 2.03 (s, 1H), 1.74 (s, 1H), 1.46 (s, 9H), 1.26 (s, 8H). ^13^C-NMR (150 MHz, DMSO-d_6_) *δ* 155.0, 151.5, 148.7, 148.7, 144.2, 142.4, 127.0, 119.3, 114.2, 109.5, 77.5, 57.1, 54.1, 50.2, 50.0, 47.0, 45.3, 30.7, 28.0. HRMS (ESI) [M + H]^+^: *m*/*z* calcd. 512.2535. Found 512.2534.

### (R)-1-(6-chloro-2-(4-(4-methylpiperazin-1-yl)phenyl)-3H-imidazo[4,5-b]pyridin-7-yl)pyrrolidin-3-amine (11)

Compound **10** (35 mg, 0.09 mmol) was dissolved in DCM (0.5 ml). Trifluoroacetic acid (TFA, 0.5 ml) was slowly, and the mixture was stirred at RT for 1 h. Saturated NaHCO_3_ was added dropwise to the reaction mixture in addition to CHCl_3_/2-propanol (4:1). The organic layer was washed with saturated NaHCO_3_ and brine, dried with Na_2_SO_4_, filtered and concentrated on a rotary evaporator to obtain **11** (26 mg, 70%). ^1^H-NMR (300 MHz, DMSO-d_6_) *δ* 7.98 (d, 2H), 7.88 (s, 1H), 7.04 (d, 2H), 4.30 (m, 2H), 4.05 (m, 1H), 3.74 (m, 1H), 3.26 (m, 7H), 2.44 (m, 4H), 2.21 (s, 3H), 2.19 (m, 1H), 1.88 (m, 1H).

### (R)-N-(1-(6-chloro-2-(4-(4-methylpiperazin-1-yl)phenyl)-3H-imidazo[4,5-b]pyridin-7-yl)pyrrolidin-3-yl)-2-cyanoacetamide (12)

Compound **11** (1.62 g, 3.93 mmol), cyanoacetic acid (505 mg, 5.90 mmol), EDCI (1.13g, 5.90 mmol), HOBt (160 mg, 1.18 mmol), and DIPEA (2.1 ml, 11.79 mmol) were dissolved in DMF (30 ml). The mixture was stirred at RT for 16 h, then diluted with EA, washed with water and brine, dried with Na_2_SO_4_, filtered, and concentrated on a rotary evaporator. The concentrated mixture was purified via MPLC to obtain **12** (1.05 g, 56%). ^1^H-NMR (600 MHz, DMSO-d_6_) *δ* 8.64 (d, *J =* 5.5 Hz, 1H), 7.99 (d, *J =* 9.0 Hz, 2H), 7.89 (d, *J =* 4.8 Hz, 1H), 7.06 (d, *J =* 9.0 Hz, 2H), 4.34 (q, *J =* 4.8 Hz, 2H), 4.29–4.26 (m, 1H), 4.17–4.14 (m, 1H), 3.98 (d, *J =* 7.6 Hz, 1H), 3.65 (s, 2H), 3.31 (s, 6H), 2.60 (s, 4H), 2.33 (s, 3H), 2.15 (d, *J =* 6.9 Hz, 1H), 1.98–1.90 (m, 2H). ^13^C-NMR (150 MHz, DMSO-d_6_) *δ* 162.2, 151.6, 148.9, 148.9, 144.7, 142.3, 127.2, 127.0, 119.6, 116.2, 114.7, 109.2, 57.2, 54.0, 50.5, 49.3, 46.8, 30.7, 25.3. HRMS (ESI) [M + H]^+^: *m*/*z* calcd. 479.2069. Found 479.2068.

### (R,E)-N-(1-(6-chloro-2-(4-(4-methylpiperazin-1-yl)phenyl)-3H-imidazo[4,5-b]pyridin-7-yl)pyrrolidin-3-yl)-2-cyano-3-phenylacrylamide (13a)

Benzaldehyde (7 µl, 0.07 mmol) and piperidine (1 µl, 0.01 mmol) were added to a solution of **12** (35 mg, 0.07 mmol) in 2-propanol (1 ml). After stirring at 60 °C for 2 h, the reaction mixture was filtered, and the filtrate was purified via MPLC to obtain **13a** (8 mg, 20%). ^1^H-NMR (600 MHz, DMSO-d_6_) *δ* 8.78 (d, *J =* 6.9 Hz, 1H), 8.14 (s, 1H), 7.99 (d, *J =* 9.0 Hz, 2H), 7.91 (s, 1H), 7.90–7.84 (m, 2H), 7.62–7.49 (m, 3H), 7.03 (d, *J =* 9.0 Hz, 2H), 4.50 (q, *J =* 5.5 Hz, 1H), 4.32 (q, *J =* 6.0 Hz, 1H), 4.29–4.19 (m, 3H), 3.24 (t, *J =* 4.8 Hz, 4H), 2.45 (t, *J =* 5.2 Hz, 4H), 2.28–2.16 (m, 4H), 2.09 (q, *J =* 6.0 Hz, 1H). ^13^C-NMR (150 MHz, DMSO-d_6_) *δ* 161.66, 151.81, 150.34, 148.99, 148.93, 144.56, 142.53, 132.21, 131.93, 129.88, 129.17, 127.23, 127.13, 119.34, 116.27, 114.48, 109.66, 106.79, 56.49, 54.39, 50.61, 50.10, 47.12, 45.72, 30.67. HRMS (ESI) [M + H]^+^: *m*/*z* calcd. 567.2382. Found 567.2379.

### (R,E)-N-(1-(6-chloro-2-(4-(4-methylpiperazin-1-yl)phenyl)-3H-imidazo[4,5-b]pyridin-7-yl)pyrrolidin-3-yl)-2-cyano-3-(4-methylthiazol-2-yl)acrylamide (13b)

Compound **13b** was synthesised as described for **13a** using 4-methylthiazole-2-carbaldehyde as an aldehyde source. ^1^H-NMR (600 MHz, DMSO-d_6_) *δ* 8.87 (d, *J =* 6.9 Hz, 1H), 8.25 (s, 1H), 7.98 (d, *J =* 9.0 Hz, 2H), 7.91 (s, 1H), 7.79 (s, 1H), 7.03 (d, *J =* 9.0 Hz, 2H), 4.49 (d, *J =* 5.5 Hz, 1H), 4.33–4.30 (m, 1H), 4.28–4.17 (m, 3H), 3.26 (s, 4H), 2.46 (s, 3H), 2.32–2.16 (m, 4H), 2.08 (d, *J =* 6.9 Hz, 1H). ^13^C-NMR (150 MHz, DMSO-d_6_) *δ* 160.98, 158.09, 155.01, 151.74, 148.96, 148.92, 144.57, 142.53, 140.88, 127.22, 127.11, 121.71, 119.40, 115.48, 114.51, 109.63, 107.68, 56.46, 54.28, 50.59, 50.17, 47.01, 45.55, 30.63, 16.59. HRMS (ESI) [M + H]^+^: *m*/*z* calcd. 588.2055. Found 588.2048.

### (R,E)-N-(1-(6-chloro-2-(4-(4-methylpiperazin-1-yl)phenyl)-3H-imidazo[4,5-b]pyridin-7-yl)pyrrolidin-3-yl)-2-cyano-3-(1-methyl-1H-imidazol-2-yl)acrylamide (13c)

Compound **13c** was synthesised as described for **13a** using 1-methyl-1H-imidazole-2-carbaldehyde as an aldehyde source. ^1^H-NMR (600 MHz, DMSO-d_6_) *δ* 8.63 (d, *J =* 6.9 Hz, 1H), 8.02–7.94 (2H), 7.94–7.83 (m, 2H), 7.51 (s, 1H), 7.29 (s, 1H), 7.04 (d, *J =* 9.0 Hz, 2H), 4.50 (q, *J =* 5.7 Hz, 1H), 4.39–4.15 (m, 4H), 3.80 (s, 3H), 3.26 (s, 4H), 2.48 (s, 4H), 2.30–2.16 (m, 4H), 2.10 (q, *J =* 5.7 Hz, 1H). ^13^C-NMR (150 MHz, DMSO-d_6_) *δ* 161.96, 151.77, 148.98, 148.94, 144.58, 142.48, 140.11, 133.30, 131.31, 127.24, 127.15, 126.45, 119.41, 115.82, 114.52, 109.59, 104.09, 56.49, 54.31, 50.64, 50.05, 47.05, 45.60, 32.82, 30.63. HRMS (ESI) [M + H]^+^: *m*/*z* calcd. 571.2444. Found 571.2440.

### (R,E)-N-(1-(6-chloro-2-(4-(4-methylpiperazin-1-yl)phenyl)-3H-imidazo[4,5-b]pyridin-7-yl)pyrrolidin-3-yl)-2-cyano-3-(1-methyl-1H-pyrazol-3-yl)acrylamide (13d)

Compound **13d** was synthesised as described for **13a** using 1-methyl-1H-pyrazole-3-carbaldehyde as an aldehyde source. ^1^H-NMR (600 MHz, DMSO-d_6_) *δ* 8.73 (d, *J =* 6.2 Hz, 1H), 8.08 (s, 1H), 7.98 (d, *J =* 9.0 Hz, 2H), 7.91 (d, *J =* 2.1 Hz, 1H), 7.90 (s, 1H), 7.03 (d, *J =* 9.0 Hz, 2H), 7.01 (d, *J =* 2.8 Hz, 1H), 4.47 (q, *J =* 5.5 Hz, 1H), 4.32 (q, *J =* 6.0 Hz, 1H), 4.29–4.14 (m, 3H), 3.94 (s, 3H), 3.25 (s, 4H), 2.46 (s, 4H), 2.29–2.14 (m, 4H), 2.09–2.07 (m, 1H). ^13^C-NMR (150 MHz, DMSO-d_6_) *δ* 161.61, 151.80, 148.93, 144.59, 144.31, 142.50, 142.26, 133.44, 127.22, 127.12, 119.41, 116.18, 114.51, 109.51, 107.10, 105.02, 56.54, 54.38, 50.59, 50.00, 47.12, 45.70, 30.65. HRMS (ESI) [M + H]^+^: *m*/*z* calcd. 571.2444. Found 571.2439.

### (R,E)-N-(1-(6-chloro-2-(4-(4-methylpiperazin-1-yl)phenyl)-3H-imidazo[4,5-b]pyridin-7-yl)pyrrolidin-3-yl)-2-cyano-3-(pyridin-2-yl)acrylamide (13e)

Compound **13e** was synthesised as described for **13a** using 2-pyridinecarboxaldehyde as an aldehyde source. ^1^H-NMR (600 MHz, DMSO-d_6_) *δ* 8.83 (d, *J =* 6.9 Hz, 1H), 8.80–8.70 (1H), 8.09 (s, 1H), 8.04–7.97 (2H), 7.96 (dd, *J =* 7.9, 1.7 Hz, 1H), 7.91 (s, 1H), 7.79 (d, *J =* 8.3 Hz, 1H), 7.53 (q, *J =* 4.1 Hz, 1H), 7.04 (t, *J =* 9.3 Hz, 2H), 4.50 (t, *J =* 5.5 Hz, 1H), 4.33 (q, *J =* 5.7 Hz, 1H), 4.30–4.18 (m, 2H), 3.24 (t, *J =* 4.8 Hz, 4H), 2.48–2.40 (m, 4H), 2.22 (d, *J =* 9.0 Hz, 4H), 2.09 (q, *J =* 5.7 Hz, 1H). ^13^C-NMR (150 MHz, DMSO-d_6_) *δ* 161.62, 151.72, 150.00, 149.91, 148.83, 148.13, 144.48, 137.37, 127.13, 127.04, 126.83, 125.98, 119.25, 115.50, 114.39, 109.43, 62.96, 56.40, 54.30, 50.51, 49.99, 47.03, 45.63, 30.57. HRMS (ESI) [M + H]^+^: *m*/*z* calcd. 568.2334. Found 568.2352.

### (R,E)-N-(1-(6-chloro-2-(4-(4-methylpiperazin-1-yl)phenyl)-3H-imidazo[4,5-b]pyridin-7-yl)pyrrolidin-3-yl)-2-cyano-3–(6-fluoropyridin-2-yl)acrylamide (13f)

Compound **13f** was synthesised as described for **13a** using 6-fluoropicolinaldehyde as an aldehyde source. ^1^H-NMR (600 MHz, DMSO-d_6_) *δ* 8.87 (d, *J =* 6.2 Hz, 1H), 8.16 (q, *J =* 8.0 Hz, 1H), 8.05 (s, 1H), 7.98 (d, *J =* 9.0 Hz, 2H), 7.91 (s, 1H), 7.74 (dd, *J =* 7.6, 2.1 Hz, 1H), 7.37 (dd, *J =* 8.3, 2.1 Hz, 1H), 7.05–7.02 (m, 3H), 4.49 (t, *J =* 5.5 Hz, 1H), 4.32 (q, *J =* 5.7 Hz, 1H), 4.29–4.19 (m, 2H), 3.25 (t, *J =* 5.2 Hz, 4H), 2.47 (d, *J =* 4.1 Hz, 4H), 2.30–2.17 (m, 4H), 2.09 (dd, *J =* 7.2, 5.2 Hz, 1H). ^13^C-NMR (150 MHz, DMSO-d_6_) *δ* 161.41, 161.08, 151.79, 149.00, 148.93, 148.33, 146.20, 144.56, 143.58, 143.52, 142.53, 127.23, 127.13, 125.27, 119.34, 115.03, 114.49, 110.49, 109.67, 56.45, 54.36, 50.62, 50.13, 47.07, 45.68, 30.66. HRMS (ESI) [M + H]^+^: *m*/*z* calcd. 586.2240. Found 586.2237.

### (R,E)-N-(1–(6-chloro-2–(4–(4-methylpiperazin-1-yl)phenyl)-3H-imidazo[4,5-b]pyridin-7-yl)pyrrolidin-3-yl)-3-(6-chloropyridin-2-yl)-2-cyanoacrylamide (13g)

Compound **13g** was synthesised as described for **13a** using 6-chloropicolinaldehyde as an aldehyde source. ^1^H-NMR (600 MHz, DMSO-d_6_) *δ* 8.88 (d, *J =* 6.9 Hz, 1H), 8.05 (s, 1H), 8.03 (t, *J =* 7.9 Hz, 1H), 7.98 (d, *J =* 9.0 Hz, 2H), 7.91 (s, 1H), 7.85–7.75 (1H), 7.66 (d, *J =* 7.6 Hz, 1H), 7.02 (d, *J =* 9.0 Hz, 2H), 4.50 (d, *J =* 5.5 Hz, 1H), 4.32 (q, *J =* 6.0 Hz, 1H), 4.24 (td, *J =* 7.4, 3.7 Hz, 2H), 3.25 (t, *J =* 4.8 Hz, 4H), 2.49–2.42 (4H), 2.23 (q, *J =* 7.1 Hz, 4H), 2.14–2.04 (m, 1H). ^13^C-NMR (150 MHz, DMSO-d_6_) *δ* 161.37, 151.75, 150.53, 150.11, 148.98, 148.92, 146.17, 144.56, 142.52, 141.06, 127.23, 127.13, 126.80, 125.86, 119.36, 114.96, 114.49, 110.77, 109.67, 56.45, 54.31, 50.61, 50.12, 47.04, 45.60, 30.66. HRMS (ESI) [M + H]^+^: *m*/*z* calcd. 602.1945. Found 602.1943.

### (R,E)-N-(1–(6-chloro-2–(4-(4-methylpiperazin-1-yl)phenyl)-3H-imidazo[4,5-b]pyridin-7-yl)pyrrolidin-3-yl)-2-cyano-3-(6-methylpyridin-2-yl)acrylamide (13h)

Compound **13h** was synthesised as described for **13a** using 6-methylpicolinaldehyde as an aldehyde source. ^1^H-NMR (600 MHz, DMSO-d_6_) *δ* 8.82 (d, *J =* 6.2 Hz, 1H), 8.04 (s, 1H), 7.99 (d, *J =* 9.0 Hz, 2H), 7.91 (s, 1H), 7.84 (t, *J =* 7.9 Hz, 1H), 7.61 (d, *J =* 7.6 Hz, 1H), 7.39 (d, *J =* 8.3 Hz, 1H), 7.03 (d, *J =* 9.0 Hz, 2H), 4.50 (q, *J =* 5.5 Hz, 1H), 4.33 (q, *J =* 5.7 Hz, 1H), 4.30–4.18 (m, 2H), 3.24 (t, *J =* 4.8 Hz, 4H), 2.46 (t, *J =* 4.8 Hz, 4H), 2.29–2.16 (m, 4H), 2.09 (q, *J =* 6.2 Hz, 1H). ^13^C-NMR (150 MHz, DMSO-d_6_) *δ* 161.80, 158.49, 151.79, 149.32, 148.96, 148.16, 144.57, 142.51, 137.56, 127.22, 127.12, 125.67, 123.88, 119.37, 115.58, 114.49, 109.60, 109.35, 56.50, 54.36, 50.60, 50.05, 47.10, 45.69, 30.66, 23.71. HRMS (ESI) [M + H]^+^: *m*/*z* calcd. 582.2491. Found 582.2494.

### (R,E)-N-(1–(6-chloro-2–(4-(4-methylpiperazin-1-yl)phenyl)-3H-imidazo[4,5-b]pyridin-7-yl)pyrrolidin-3-yl)-2-cyano-3-(6-cyclopropylpyridin-2-yl)acrylamide (13i)

Compound **13i** was synthesised as described for **13a** using 6-cyclopropylpicolinaldehyde as an aldehyde source. ^1^H-NMR (600 MHz, DMSO-d_6_) *δ* 8.76 (d, *J =* 6.9 Hz, 1H), 7.99 (s, 1H), 7.97 (s, 2H), 7.91 (s, 1H), 7.77 (t, *J =* 7.9 Hz, 1H), 7.48 (d, *J =* 7.6 Hz, 1H), 7.43 (d, *J =* 7.6 Hz, 1H), 7.03 (d, *J =* 9.0 Hz, 2H), 4.49 (d, *J =* 5.5 Hz, 1H), 4.32 (q, *J =* 6.0 Hz, 1H), 4.29–4.17 (m, 3H), 3.24 (t, *J =* 4.8 Hz, 4H), 2.48–2.40 (m, 4H), 2.28–2.18 (m, 4H), 2.18–2.11 (m, 1H), 2.08 (dd, *J =* 7.2, 5.2 Hz, 1H), 1.20–1.11 (m, 2H), 1.01–0.89 (m, 2H). ^13^C-NMR (150 MHz, DMSO-d_6_) *δ* 163.41, 161.90, 151.80, 149.34, 148.92, 148.32, 144.56, 142.51, 137.15, 127.22, 127.13, 124.68, 124.31, 119.34, 115.90, 114.48, 109.63, 108.99, 56.50, 54.39, 50.59, 50.02, 47.11, 45.71, 30.67, 17.11, 10.33. HRMS (ESI) [M + H]^+^: *m*/*z* calcd. 608.2648. Found 608.2641.

### (R,E)-N-(1–(6-chloro-2–(4-(4-methylpiperazin-1-yl)phenyl)-3H-imidazo[4,5-b]pyridin-7-yl)pyrrolidin-3-yl)-2-cyano-3-(5-methylpyridin-2-yl)acrylamide (13j)

Compound **13j** was synthesised as described for **13a** using 5-methylpicolinaldehyde as an aldehyde source. ^1^H-NMR (600 MHz, DMSO-d_6_) *δ* 8.79 (d, *J =* 6.2 Hz, 1H), 8.60 (d, *J =* 1.4 Hz, 1H), 8.05 (s, 1H), 7.98 (d, *J =* 9.0 Hz, 2H), 7.91 (s, 1H), 7.77 (dd, *J =* 8.3, 1.4 Hz, 1H), 7.70 (d, *J =* 7.6 Hz, 1H), 7.03 (t, *J =* 8.3 Hz, 2H), 4.50 (q, *J =* 5.7 Hz, 1H), 4.33 (q, *J =* 6.0 Hz, 1H), 4.30–4.18 (m, 2H), 3.24 (t, *J =* 4.8 Hz, 4H), 2.47 (t, *J =* 4.8 Hz, 4H), 2.37 (s, 3H), 2.29–2.16 (m, 4H), 2.09 (q, *J =* 6.2 Hz, 1H). ^13^C-NMR (150 MHz, DMSO-d_6_) *δ* 161.85, 151.78, 150.55, 148.93, 148.30, 147.47, 144.57, 142.52, 137.41, 136.36, 127.24, 127.14, 126.50, 119.38, 115.74, 114.50, 109.63, 108.43, 56.50, 54.36, 50.61, 50.07, 47.08, 45.67, 30.67, 18.17. HRMS (ESI) [M + H]^+^: *m*/*z* calcd. 582.2491. Found 582.2508.

### (R,E)-N-(1–(6-chloro-2–(4-(4-methylpiperazin-1-yl)phenyl)-3H-imidazo[4,5-b]pyridin-7-yl)pyrrolidin-3-yl)-2-cyano-3-(5-methoxypyridin-2-yl)acrylamide (13k)

Compound **13k** was synthesised as described for **13a** using 5-methoxypicolinaldehyde as an aldehyde source. ^1^H-NMR (600 MHz, DMSO-d_6_) *δ* 8.71 (d, *J =* 6.2 Hz, 1H), 8.47 (d, *J =* 2.8 Hz, 1H), 8.05 (s, 1H), 7.99 (d, *J =* 8.3 Hz, 2H), 7.91 (s, 1H), 7.82 (d, *J =* 9.0 Hz, 1H), 7.52 (dd, *J =* 8.6, 3.1 Hz, 1H), 7.03 (d, *J =* 9.0 Hz, 2H), 4.50 (q, *J =* 5.7 Hz, 1H), 4.33 (q, *J =* 6.0 Hz, 1H), 4.30–4.18 (m, 3H), 3.93 (d, *J =* 18.6 Hz, 3H), 3.24 (t, *J =* 4.8 Hz, 4H), 2.46 (t, *J =* 4.5 Hz, 4H), 2.29–2.16 (m, 4H), 2.10 (q, *J =* 6.0 Hz, 1H). ^13^C-NMR (150 MHz, DMSO-d_6_) *δ* 162.01, 156.96, 151.77, 148.97, 148.93, 147.86, 144.55, 142.51, 142.36, 138.80, 128.62, 127.23, 127.13, 120.20, 119.38, 116.01, 114.48, 109.62, 106.35, 56.50, 55.99, 54.35, 50.61, 50.05, 47.09, 45.67, 30.67. HRMS (ESI) [M + H]^+^: *m*/*z* calcd. 598.2440. Found 598.2434.

### (R,E)-N-(1-(6-chloro-2-(4-(4-methylpiperazin-1-yl)phenyl)-3H-imidazo[4,5-b]pyridin-7-yl)pyrrolidin-3-yl)-2-cyano-3–(4-methylpyridin-2-yl)acrylamide (13l)

Compound **13l** was synthesised as described for **13a** using 4-methylpicolinaldehyde as an aldehyde source. ^1^H-NMR (600 MHz, DMSO-d_6_) *δ* 8.81 (d, *J =* 6.9 Hz, 1H), 8.59 (d, *J =* 4.8 Hz, 1H), 8.01 (s, 1H), 7.99 (d, *J =* 8.3 Hz, 2H), 7.92 (s, 1H), 7.57 (s, 1H), 7.36 (d, *J =* 4.8 Hz, 1H), 7.03 (t, *J =* 8.3 Hz, 2H), 4.49 (t, *J =* 5.5 Hz, 1H), 4.36–4.18 (m, 3H), 3.24 (t, *J =* 4.8 Hz, 4H), 2.46 (t, *J =* 4.8 Hz, 4H), 2.35 (s, 3H), 2.29–2.16 (4H), 2.10 (q, *J =* 6.0 Hz, 1H). ^13^C-NMR (150 MHz, DMSO-d_6_) *δ* 161.74, 151.80, 149.94, 149.73, 148.98, 148.93, 148.34, 148.19, 144.55, 142.56, 127.74, 127.43–127.16 (0 °C), 127.14, 126.70, 119.34, 115.60, 114.48, 109.73, 109.40, 56.46, 54.37, 50.58, 50.10, 47.09, 45.69, 30.65, 20.35. HRMS (ESI) [M + H]^+^: *m*/*z* calcd. 582.2491. Found 582.2503.

### (R,E)-N-(1–(6-chloro-2–(4-(4-methylpiperazin-1-yl)phenyl)-3H-imidazo[4,5-b]pyridin-7-yl)pyrrolidin-3-yl)-2-cyano-3-(pyridin-3-yl)acrylamide (13m)

Compound **13m** was synthesised as described for **13a** using nicotinaldehyde as an aldehyde source. ^1^H-NMR (600 MHz, DMSO-d_6_) *δ* 8.92 (d, *J =* 2.1 Hz, 1H), 8.86 (d, *J =* 6.9 Hz, 1H), 8.73–8.66 (1H), 8.36–8.27 (1H), 8.23–8.14 (1H), 7.98 (d, *J =* 9.0 Hz, 2H), 7.92 (s, 1H), 7.59 (q, *J =* 4.4 Hz, 1H), 7.10–6.97 (m, 2H), 4.50 (q, *J =* 5.7 Hz, 1H), 4.37–4.18 (m, 3H), 3.25 (t, *J =* 4.8 Hz, 4H), 2.49 (d, *J =* 4.1 Hz, 4H), 2.23 (q, *J =* 6.9 Hz, 4H), 2.09 (q, *J =* 6.0 Hz, 1H). ^13^C-NMR (150 MHz, DMSO-d_6_) *δ* 161.19, 152.24, 151.75, 151.03, 149.00, 148.93, 147.44, 144.55, 142.57, 135.81, 128.10, 127.23, 127.11, 124.05, 119.33, 115.89, 114.49, 109.73, 108.98, 56.41, 54.29, 50.65, 50.14, 47.02, 45.59, 30.66. HRMS (ESI) [M + H]^+^: *m*/*z* calcd. 568.2335. Found 568.2336.

### (R,E)-N-(1–(6-chloro-2–(4-(4-methylpiperazin-1-yl)phenyl)-3H-imidazo[4,5-b]pyridin-7-yl)pyrrolidin-3-yl)-2-cyano-3-(6-methylpyridin-3-yl)acrylamide (13n)

Compound **13n** was synthesised as described for **13a** using 6-methylnicotinaldehyde as an aldehyde source. ^1^H-NMR (600 MHz, DMSO-d_6_) *δ* 8.80 (t, *J =* 6.2 Hz, 2H), 8.23 (dd, *J =* 8.3, 2.1 Hz, 1H), 8.14 (s, 1H), 7.98 (d, *J =* 9.0 Hz, 2H), 7.91 (s, 1H), 7.45 (d, *J =* 8.3 Hz, 1H), 7.02 (d, *J =* 9.0 Hz, 2H), 4.50 (d, *J =* 5.5 Hz, 1H), 4.35–4.26 (m, 2H), 4.24 (t, *J =* 6.9 Hz, 2H), 3.24 (d, *J =* 4.1 Hz, 4H), 2.59–2.52 (3H), 2.46 (d, *J =* 4.1 Hz, 4H), 2.22 (d, *J =* 13.8 Hz, 4H), 2.09 (dd, *J =* 6.9, 4.8 Hz, 1H). ^13^C-NMR (150 MHz, DMSO-d_6_) *δ* 161.80, 161.37, 151.80, 150.92, 149.01, 148.93, 147.47, 144.55, 142.57, 135.78, 127.23, 127.12, 125.33, 123.46, 119.30, 116.10, 114.46, 109.74, 107.64, 56.42, 54.37, 50.64, 50.11, 47.09, 45.70, 30.68, 24.26. HRMS (ESI) [M + H]^+^: *m*/*z* calcd. 582.2491. Found 582.2490.

### (R,E)-N-(1–(6-chloro-2–(4-(4-methylpiperazin-1-yl)phenyl)-3H-imidazo[4,5-b]pyridin-7-yl)pyrrolidin-3-yl)-2-cyano-3-(2-methylpyridin-3-yl)acrylamide (13o)

Compound **13o** was synthesised as described for **13a** using 2-methylnicotinaldehyde as an aldehyde source. ^1^H-NMR (600 MHz, DMSO-d_6_) *δ* 8.85 (d, *J =* 6.9 Hz, 1H), 8.55 (q, *J =* 2.1 Hz, 1H), 8.30 (s, 1H), 8.08 (d, *J =* 6.9 Hz, 1H), 7.99 (d, *J =* 9.0 Hz, 2H), 7.92 (s, 1H), 7.38 (q, *J =* 4.1 Hz, 1H), 7.02 (d, *J =* 9.0 Hz, 2H), 4.51 (t, *J =* 5.5 Hz, 1H), 4.27 (m, 3H), 3.24 (t, *J =* 4.8 Hz, 4H), 2.52 (s, 3H), 2.49–2.40 (m, 4H), 2.24 (t, *J =* 9.3 Hz, 4H), 2.12 (t, *J =* 5.9 Hz, 1H). ^13^C-NMR (150 MHz, DMSO-d_6_) *δ* 160.98, 157.44, 151.79, 151.04, 149.04, 148.94, 148.05, 144.55, 142.59, 135.53, 127.25, 127.14, 127.03, 121.46, 119.33, 115.53, 114.48, 111.24, 109.80, 56.37, 54.35, 50.83–50.47 (0 °C), 50.16, 47.07, 45.67, 30.63, 22.69. HRMS (ESI) [M + H]^+^: *m*/*z* calcd. 582.2491. Found 582.2489.

### (R,E)-N-(1–(6-chloro-2–(4-(4-methylpiperazin-1-yl)phenyl)-3H-imidazo[4,5-b]pyridin-7-yl)pyrrolidin-3-yl)-2-cyano-3-(4-methylpyridin-3-yl)acrylamide (13p)

Compound **13p** was synthesised as described for **13a** using 4-methylnicotinaldehyde as an aldehyde source. ^1^H-NMR (600 MHz, DMSO-d_6_) *δ* 8.85 (d, *J =* 6.9 Hz, 1H), 8.81–8.73 (1H), 8.53 (d, *J =* 4.8 Hz, 1H), 8.29 (s, 1H), 7.98 (d, *J =* 9.0 Hz, 2H), 7.92 (s, 1H), 7.38 (d, *J =* 4.8 Hz, 1H), 7.02 (d, *J =* 9.0 Hz, 2H), 4.51 (q, *J =* 5.5 Hz, 1H), 4.35–4.19 (m, 3H), 3.24 (d, *J =* 4.8 Hz, 4H), 2.46 (t, *J =* 4.8 Hz, 4H), 2.34 (s, 3H), 2.30–2.17 (m, 4H), 2.11 (q, *J =* 6.2 Hz, 1H). ^13^C-NMR (150 MHz, DMSO-d_6_) *δ* 160.87, 151.81, 151.27, 149.03, 148.93, 147.98, 147.11, 146.92, 144.54, 142.56, 128.51, 127.23, 127.16–126.96 (0 °C), 125.31, 119.31, 115.65, 114.47, 111.21, 109.76, 56.36, 54.37, 50.65, 50.15, 47.08, 45.70, 30.62, 18.76. HRMS (ESI) [M + H]^+^: *m*/*z* calcd. 582.2491. Found 582.2486.

### (R,E)-N-(1–(6-chloro-2–(4-(4-methylpiperazin-1-yl)phenyl)-3H-imidazo[4,5-b]pyridin-7-yl)pyrrolidin-3-yl)-2-cyano-3-(pyridin-4-yl)acrylamide (13q)

Compound **13q** was synthesised as described for **13a** using isonicotinaldehyde as an aldehyde source. ^1^H-NMR (600 MHz, DMSO-d_6_) *δ* 8.94 (d, *J =* 6.2 Hz, 1H), 8.76 (q, *J =* 2.1 Hz, 1H), 8.13 (s, 1H), 8.08–7.94 (2H), 7.92 (s, 1H), 7.71 (d, *J =* 6.2 Hz, 1H), 7.04 (dd, *J =* 13.1, 9.0 Hz, 2H), 4.50 (t, *J =* 5.5 Hz, 1H), 4.38–4.20 (m, 3H), 3.26 (t, *J =* 4.8 Hz, 4H), 2.51 (s, 4H), 2.33–2.17 (m, 4H), 2.10 (t, *J =* 5.9 Hz, 1H). ^13^C-NMR (150 MHz, DMSO-d_6_) *δ* 160.93, 151.73, 150.62, 150.14, 149.00, 148.93, 147.95, 144.57, 142.57, 139.07, 127.26, 127.12, 122.89, 122.82, 119.39, 115.32, 114.52, 111.45, 109.74, 56.42, 54.22, 50.63, 50.18, 46.95, 45.47, 30.66. HRMS (ESI) [M + H]^+^: *m*/*z* calcd. 568.2335. Found 568.2335.

### (R,E)-N-(1–(6-chloro-2–(4-(4-methylpiperazin-1-yl)phenyl)-3H-imidazo[4,5-b]pyridin-7-yl)pyrrolidin-3-yl)-3-(2-chloropyridin-4-yl)-2-cyanoacrylamide (13r)

Compound **13r** was synthesised as described for **13a** using 2-chloroisonicotinaldehyde as an aldehyde source. ^1^H-NMR (600 MHz, DMSO-d_6_) *δ* 8.96 (d, *J =* 6.2 Hz, 1H), 8.59 (d, *J =* 5.5 Hz, 1H), 8.11 (s, 1H), 7.97 (d, *J =* 8.3 Hz, 2H), 7.92 (s, 1H), 7.77 (s, 1H), 7.73 (t, *J =* 2.4 Hz, 1H), 7.02 (d, *J =* 9.0 Hz, 2H), 4.50 (q, *J =* 5.0 Hz, 1H), 4.33 (dd, *J =* 11.4, 3.8 Hz, 1H), 4.27 (q, *J =* 6.0 Hz, 1H), 4.22 (t, *J =* 6.9 Hz, 2H), 3.24 (s, 4H), 2.47 (s, 4H), 2.27–2.21 (m, 4H), 2.14–2.03 (m, 1H). ^13^C-NMR (150 MHz, DMSO-d_6_) *δ* 160.56, 151.77, 151.04, 150.95, 149.05, 148.93, 146.44, 144.52, 142.75, 142.64, 127.25, 127.13, 123.53, 121.93, 119.26, 115.00, 114.46, 112.61, 109.96, 56.28, 54.31, 50.65, 50.25, 47.01, 45.61, 30.64. HRMS (ESI) [M + H]^+^: *m*/*z* calcd. 602.1945. Found 602.1937.

### (R,E)-N-(1–(6-chloro-2–(4-(4-methylpiperazin-1-yl)phenyl)-3H-imidazo[4,5-b]pyridin-7-yl)pyrrolidin-3-yl)-2-cyano-3–(2-methoxypyridin-4-yl)acrylamide (13s)

Compound **13 s** was synthesised as described for **13a** using 2-methoxyisonicotinaldehyde as an aldehyde source. ^1^H-NMR (600 MHz, DMSO-d_6_) *δ* 8.91 (d, *J =* 6.9 Hz, 1H), 8.32 (t, *J =* 4.1 Hz, 1H), 8.07 (s, 1H), 7.98 (d, *J =* 9.0 Hz, 2H), 7.92 (s, 1H), 7.32 (d, *J =* 5.5 Hz, 1H), 7.14 (s, 1H), 7.02 (d, *J =* 9.0 Hz, 2H), 4.49 (d, *J =* 5.5 Hz, 1H), 4.29 (d, *J =* 4.8 Hz, 2H), 4.23 (t, *J =* 6.5 Hz, 2H), 3.89 (s, 3H), 3.24 (d, *J =* 4.1 Hz, 4H), 2.46 (d, *J =* 4.1 Hz, 4H), 2.25–2.16 (m, 4H), 2.14–2.03 (m, 1H). ^13^C-NMR (150 MHz, DMSO-d_6_) *δ* 164.13, 160.91, 151.80, 149.02, 148.92, 148.04, 147.73, 144.54, 142.04, 127.23, 127.12, 119.29, 115.81, 115.29, 114.66–114.30 (0 °C), 111.41, 110.40, 109.79, 56.37, 54.35, 53.52, 50.78–50.45 (0 °C), 50.17, 47.07, 45.68, 30.64. HRMS (ESI) [M + H]^+^: *m*/*z* calcd. 598.2440. Found 598.2435.

### (R)-N-(1–(5-chloro-2-((4–(4-methylpiperazin-1-yl)phenyl)amino)pyrimidin-4-yl)pyrrolidin-3-yl)acrylamide (14)

Acryloyl chloride (4 µl, 0.05 mmol) was added to a solution of **11** (21 mg, 0.05 mmol) and Na_2_CO_3_ (16 mg, 0.15 mmol) in 1 ml aqueous THF (THF/H_2_O 3:1) at 0 °C, and the mixture was stirred at 0 °C for 2 h. The reaction mixture was extracted with DCM three times. The extract washed with brine, dried with Na_2_SO_4_ and concentrated on a rotary evaporator. The concentrate was then purified via MPLC to obtain **14** (11 mg, 47%). ^1^H-NMR (600 MHz, DMSO-d_6_) *δ* 9.00 (s, 1H), 8.42 (d, *J =* 6.9 Hz, 1H), 7.91 (s, 1H), 7.55 (d, *J =* 9.0 Hz, 2H), 6.84 (d, *J =* 9.0 Hz, 2H), 6.24 (dd, *J =* 17.2, 10.3 Hz, 1H), 6.13 (dd, *J =* 16.9, 2.4 Hz, 1H), 5.60 (dd, *J =* 10.3, 2.1 Hz, 1H), 4.39 (q, *J =* 5.0 Hz, 1H), 3.96 (q, *J =* 5.7 Hz, 1H), 3.90–3.75 (m, 2H), 3.67 (dd, *J =* 11.7, 3.4 Hz, 1H), 3.56–3.36 (1H), 3.09–2.98 (4H), 2.45 (t, *J =* 4.8 Hz, 4H), 2.21 (s, 3H), 2.12 (q, *J =* 6.2 Hz, 1H), 1.91 (t, *J =* 5.9 Hz, 1H). ^13^C-NMR (150 MHz, DMSO-d_6_) *δ* 164.53, 157.44, 156.21, 145.76, 133.01, 131.44, 125.46, 119.75, 115.82, 102.08, 54.69, 54.13, 48.86, 48.51, 47.15, 45.69, 30.29. HRMS (ESI) [M + H]^+^: calcd. 442.2117. Found 442.2120.

### HPLC analysis for reversible addition of BME to 13h

Phosphate-buffered saline (PBS) was prepared by mixing a solution of 91.2 mg monobasic potassium phosphate (KH_2_PO_4_) in 10 ml H_2_O and a solution of 116.7 mg dibasic potassium phosphate (K_2_HPO_4_) in 10 ml H_2_O. The 0.067 M phosphate solutions were mixed to obtain a pH 7.4 phosphate buffer. A solution of 48 mM β-mercaptoethanol (BME) in PBS (0.25 ml) was added to a solution of **13h** (1 mg, ∼2 µmol) in dimethylsulphoxide (DMSO, 0.75 ml). Analysis of the reaction mixture after 30 min showed full conversion to the BME adduct. To determine whether the reaction between **13h** and BME was reversible, the mixture was diluted 1:10 with PBS in DMSO and analysed via HPLC and mass spectra. The analysis was performed using a Waters HPLC system equipped with a 1525 pump, a PDA2998 detector, and a SunFire C18 column (4.6 × 150 mm, 5 μm). The eluent system consisted of 0.05% TFA in 8:2 to 1:9 water/acetonitrile.

### Kinase assays

Kinase assays were performed by Invitrogen (now Thermo Fisher Scientific, Waltham, MA) or Reaction Biology Corp. (Malvern, PA). Inhibitory activity of compounds for TAK1 was evaluated using LanthaScreen^®^ Eu Kinase Binding Assay (Invitrogen, Waltham, MA). The kinase profile of compound **13h** was determined by the kinase HotSpot Profiling service of Reaction Biology Corporation. All assays were performed at *K*m for ATP.

### Cell culture

MDA-MB-231 cells were obtained from the Korean Cell Line Bank. Cells were cultured in Roswell Park Memorial Institute (RPMI) 1640 medium containing 10% foetal bovine serum and 1% penicillin/streptomycin at 37 °C with 5% CO_2_ under humidified atmosphere.

### Caspase-3/7 assay

The assay was performed using Apo-ONE^®^ Homogeneous Caspase-3/7 Assay kits (Catalogue No. G7790, Promega, Madison, WI). MDA-MB-231 cells in a concentration of 5000 cells/100 µl were seeded in each well of a black 96-well plate and starved after adhering to the plate. The cells were incubated for 24 h, and the serum-starved cells were treated with either Takinib or **13h** in the presence or absence of TNFα (10 ng/ml). All samples and the control contained DMSO in a final concentration of 0.5%. After another 24 h of incubation, 100 µL of Apo-ONE^®^ caspase-3/7 reagent was added to each well, and the cells were incubated at room temperature in darkness for 2 h. Fluorescence was then measured at 530 nm with excitation at 485 nm using a FlexStation 3 Multi-Mode microplate reader (Molecular Devices). Statistical analyses of the data included a one-way ANOVA, followed by Tukey’s multiple comparison test.

## Results and discussion

**Scheme 1.**
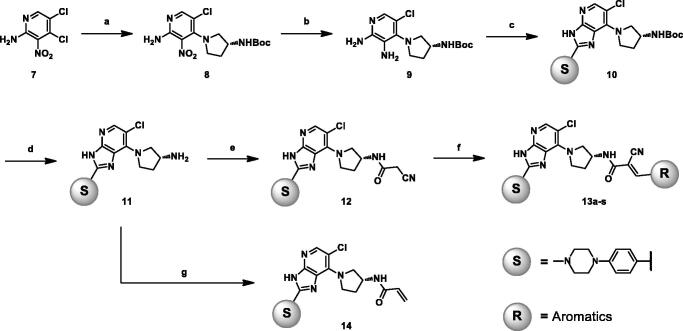
Reagents and conditions: (a) (*R*)-3-Boc-aminopyrrolidine, K_2_CO_3_, MeCN, RT, 17 h, 53%; (b) Fe powder, AcOH, 40 °C, 1 h, 66%; (c) 4–(4-Methylpiperazin-1-yl)benzaldehyde, FeCl_3_, DMF, 120 °C, 16 h, 32%; (d) TFA, DCM, RT, 1 h, 70%; (e) Cyanoacetic acid, EDCI, HOBt, DIPEA, DMF, RT, 16 h, 56%; (f) Aldehyde, piperidine, 2-propanol, 60 °C, 2 h, 20%; (g) Na_2_CO_3_, aqueous THF, acryloyl chloride, 0 °C, 2 h, 47%.

Transforming the irreversible terminal acrylamide to 2-cyanoacrylamide was key for the synthesis of reversible derivatives. The synthetic route for the imidazopyridine derivatives is outlined in Scheme 1. Synthesis of target molecules commenced with nucleophilic addition of aminopyrrolidine to **7,** which gave **8** in a moderate yield. The nitro compound (**8**) was reduced in the presence of Fe and acetic acid at 40 °C to yield **9**, which was followed by ring closure with 4–(4-methylpiperazin-1-yl)benzaldehyde to give imidazopyridine **10**^24^. The Boc group was removed under acidic conditions, and a subsequent reaction with cyanoacetic acid provided the key intermediate (**12**). Target compounds **13a–s** were obtained from **12** via Knoevenagel condensation with various aldehydes. Irreversible derivative **14** was synthesised by reacting **11** with acryloyl chloride at 0 °C.

**Figure 3.**
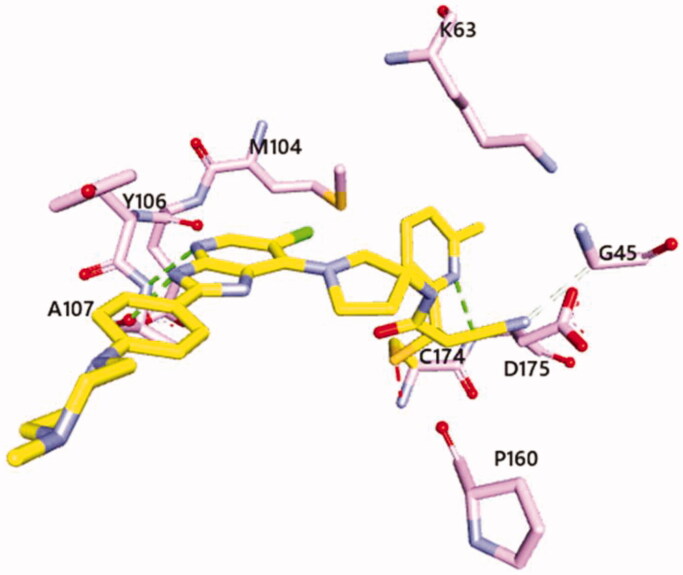
Predicted binding mode of **13h** with C174 of TAK1 kinase domain (PDB: 4L52)^25^. Imidazopyridine core interacts with hinge region of TAK1 and pyridinyl nitrogen forms a hydrogen bond with D175. The estimated free energy of binding was found to be −9.65 kcal/mol and −7.05 kcal/mol for **13h** and **14**, respectively. Covalent docking study was performed using Autodock via flexible side chain method^26^. The figure was visualised using Discovery Studio 2020 Visualiser.

A structure-activity relationship (SAR) study was performed to optimise the R group on the 2-cyanoacrylamide moiety (Table 1). Phenyl derivative **13a** had an IC_50_ of 385 nM, which was ∼2.5-fold lower than the IC_50_ of **14**. Among the compounds with five-membered heterocycles (**13b–d)**, the 4-methylthiazolyl derivative (**13b**) exhibited the highest potency. Conversion of the phenyl group (**13a**) to pyridine without a substituent afforded **13e**, **13m**, and **13q**, which had activities that were 8-fold to 14-fold higher. Further SAR analysis was performed for substituted pyridine derivatives. The IC_50_ values of the 2-pyridinyl derivatives increased with additional bulky substituents on the aromatic ring (e.g. **13g**, **13i**, and **13k**) whereas small substituents (e.g. **13f** and **13h**) maintained or improved potency. Interestingly, the potency of the derivatives with methyl substituents (**13h**, **13j**, and **13l**) was excellent regardless of the methyl position. Unlike the 2-pyridinyl derivatives, the position of the methyl substituent affected the activity of 3-pyridinyl derivatives (**13n**–**p**). Introducing a substituent to the 4-pyridinyl group resulted in lower activity (**13q**–**s**). Covalent docking studies of the **13h** and **14** with C174 of the TAK1 kinase domain were conducted. The free energies of binding were estimated to be −9.65 kcal/mol and −7.05 kcal/mol for **13h** and **14**, respectively. Imidazopyridine of both compounds formed two hydrogen bonds with A107 in the hinge region. The pyridinyl group of **13h** occupied the back pocket of TAK1 and formed a hydrogen bonding with D175. These results correspond to the TAK1 inhibitory activity of **13h** and **14** (Figure 3). A representative imidazopyridine derivative (**13h**) had an IC_50_ of 27 nM for TAK1, but inhibition of other MAP kinases by **13h** (1 µM) was as low as 10–15% (Table 2). Although limited, the kinase profile indicated that **13h** was selective for TAK1 over other MAP kinases and BRAF.

**Table 1.** TAK1 enzymatic assay with imidazopyridine derivatives.

^a^ *In-vitro* enzymatic assay data obtained from Invitrogen^TM^.

**Table 2.** Kinase profile of **13h** (1 µM).

Kinase	% inhibition^a^
ASK1/MAP3K5	4 ± 0
BRAF	0 ± 0.4
MEK1/MAP2K1	10 ± 0.7
MEK2/MAP2K2	23 ± 1.9
MEKK1	12 ± 0.7
MEKK2	0 ± 0.6
MEKK6	0 ± 6.3
MINK1/MAP4K6	15 ± 2.4
MLK1/MAP3K9	35 ± 1.2
MLK2/MAP3K10	30 ± 2
ZAK/MLTK	29 ± 1.4

^a^ *In-vitro* enzymatic assay was performed by Reaction Biology Corp.

**Scheme 2.**
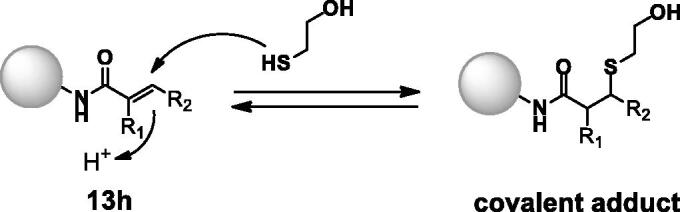
Compound **13h** reversibly reacted with β-mercaptoethanol (BME).

To evaluate the reversible covalent properties of the described compounds, we reacted **13h** with β-mercaptoethanol (BME) and determined the reversibility of covalent adduct formation using a previously reported method (Scheme 2)^22^.

The reaction between **13h** and BME generated a covalent adduct, which was identified via high-resolution mass spectrometry (HRMS, Figure S1). The BME adduct mixture was diluted 10-fold to confirm that adduct formation was reversible. After dilution, the BME adduct gradually reverted to **13h** (Figure S1).

**Figure 4.**
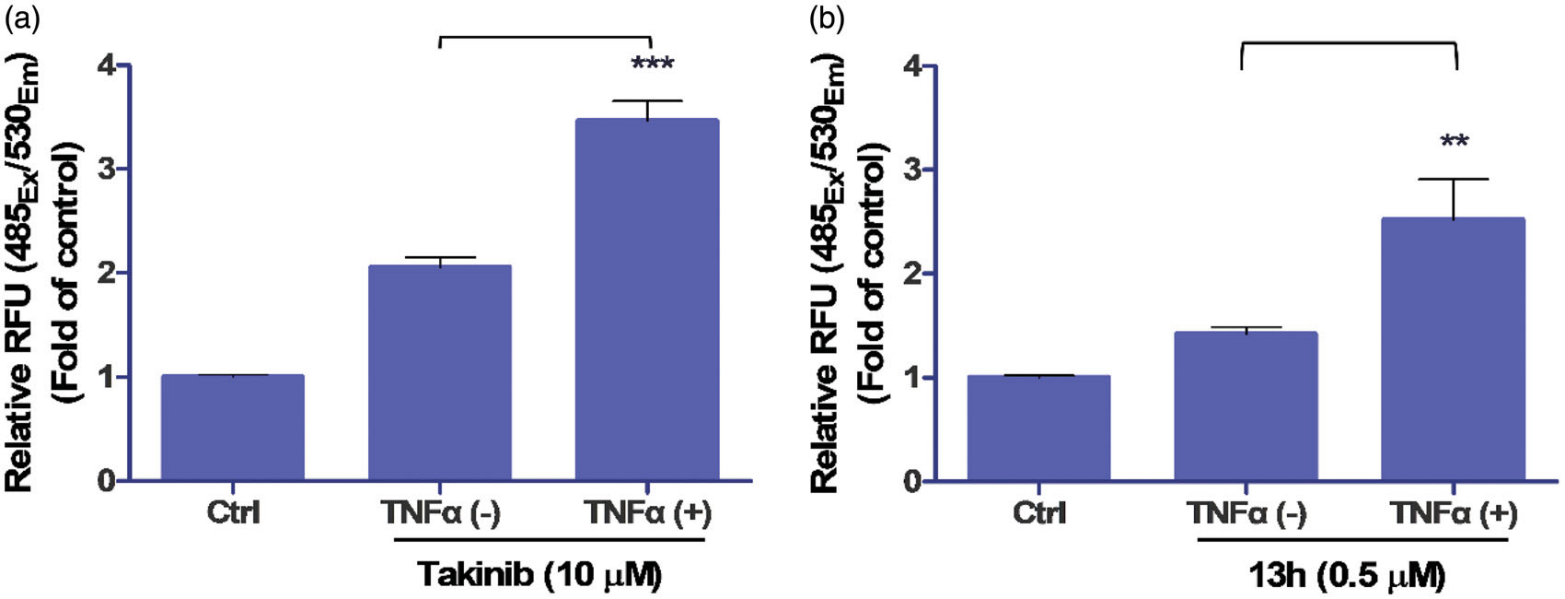
Effect of **13h** on MDA-MB-231 cells. Caspase-3/7 activity of (a) Takinib (10 µM) and (b) **13h** (0.5 µM) in the presence or absence of TNFα. Data are reported as the mean ± SD (*n* = 3). *** *p* < 0.001, ** *p* < 0.01.

TAK1 inhibition has been shown to induce the apoptosis of TNFα-stimulated breast cancer cells^11^. To assess its activity in a cell-based model, the caspase-3/7 activity of **13h** was measured in MDA-MB-231 cells. Takinib, a potent TAK1 inhibitor^11^, was used as a positive control. Like Takinib, **13h** (0.5 µM) induced significant caspase activation in the presence of TNFα, indicating that **13h** strongly inhibited TAK1 in the cells (Figure 4).

## Conclusions

We discovered potent imidazopyridine TAK1 inhibitors derived from 2-cyanoacrylamide-bearing pyrimidine derivatives. The introduction of the phenyl group into 2-cyanoacrylamide moiety led to increased activity. Among substituents of 2-cyanoacrylamide, pyridines exhibited better activity than the phenyl group or 5-membered heterocycles. These data indicated that aryl group of 2-cyanoacrylamide should provide a contribution to the interaction with TAK1. We postulate that they will act as reversible covalent TAK1 inhibitors based on the reversible reaction between **13h** and BME. Our results may contribute to the identification of novel kinase inhibitors or reversible covalent inhibitors.

## Supplementary Material

Supplemental Material
