# Supplementary material for: Synthesis and biological activity of 2-cyanoacrylamide derivatives tethered to imidazopyridine as TAK1 inhibitors
Source: J Enzyme Inhib Med Chem. 2020 Oct 22;35(1):1928–36. doi: 10.1080/14756366.2020.1833876 (PMC7594721; doi:10.1080/14756366.2020.1833876)
Supplement: Supplemental Material [file IENZ_A_1833876_SM6475.pdf]

Supporting Information

Synthesis and biological activity of 2-cyanoacrylamide derivatives tethered to imidazopyridine as TAK1 inhibitors

Seok Jong Kang[a,b,†], Jung Wuk Lee[a, †], Jiho Song[a], Jiwon Park[a], Jaeyul Choi[b], Kwee Hyun Suh[b], and Kyung Hoon Min[a,*]

[a]College of Pharmacy, Chung-Ang University, Seoul 06974, Republic of Korea

[b]Hanmi Research Center, Hanmi Pharm. Co. Ltd., Gyeonggi-Do 18469, Republic of Korea

[†]These authors are contributed equally to this work.

* Corresponding author, E-mail: khmin@cau.ac.kr

Table of Contents

1. Supplementary Figures 3

2. NMR spectral data of compound **8-14** 8

1. Supplementary Figures

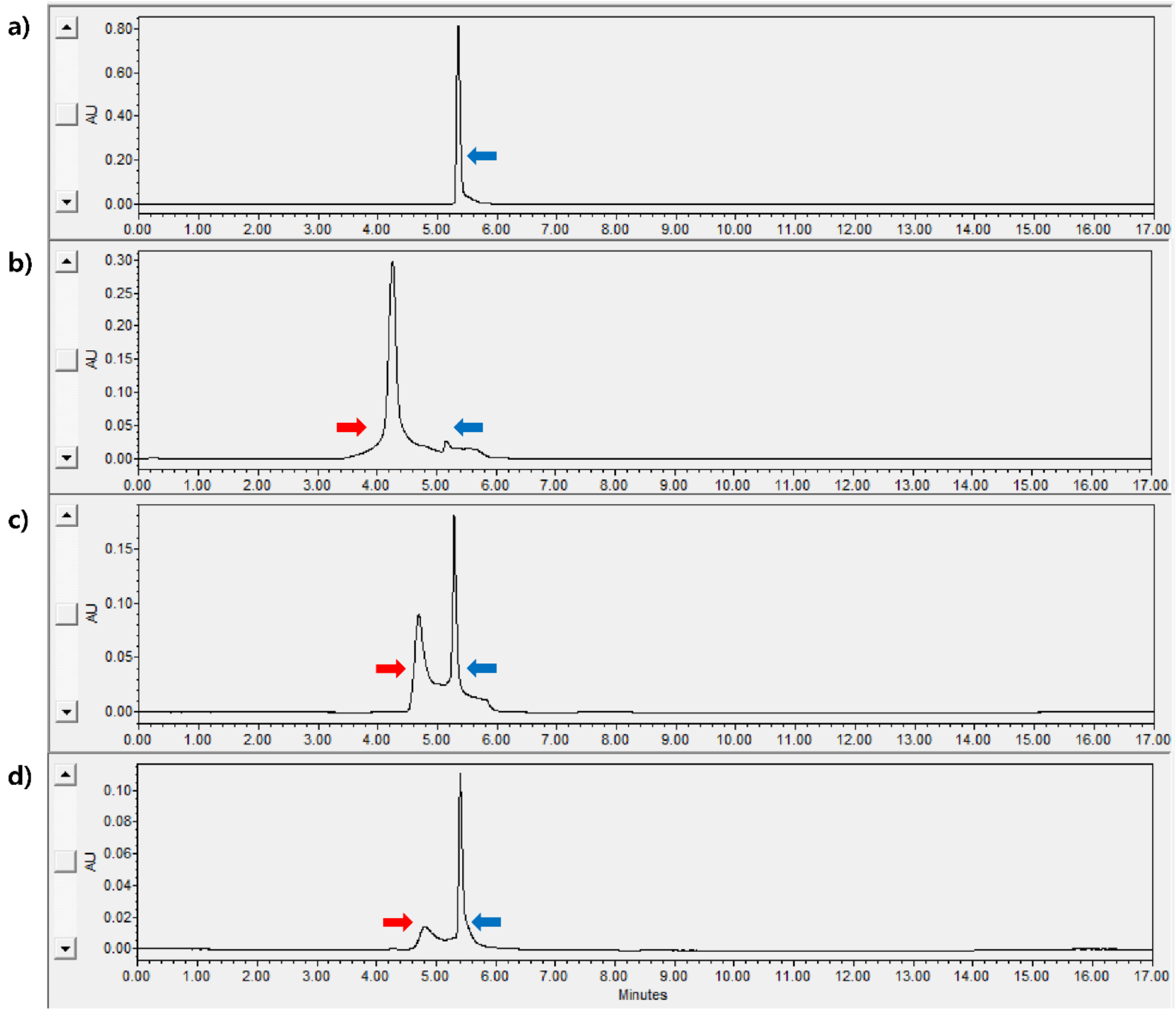

Figure S1. Time course HPLC analysis of **13h** with addition of BME. Red and blue arrows point to the peaks of the BME – **13h** adduct and **13h**, respectively. **(a)** **13h** only, **(b)** 30 min after mixing **13h** with BME, **(c)** 30 min after dilution of BME adduct and **13h** mixture with buffer, **(d)** 60 min after dilution of mixture with buffer.

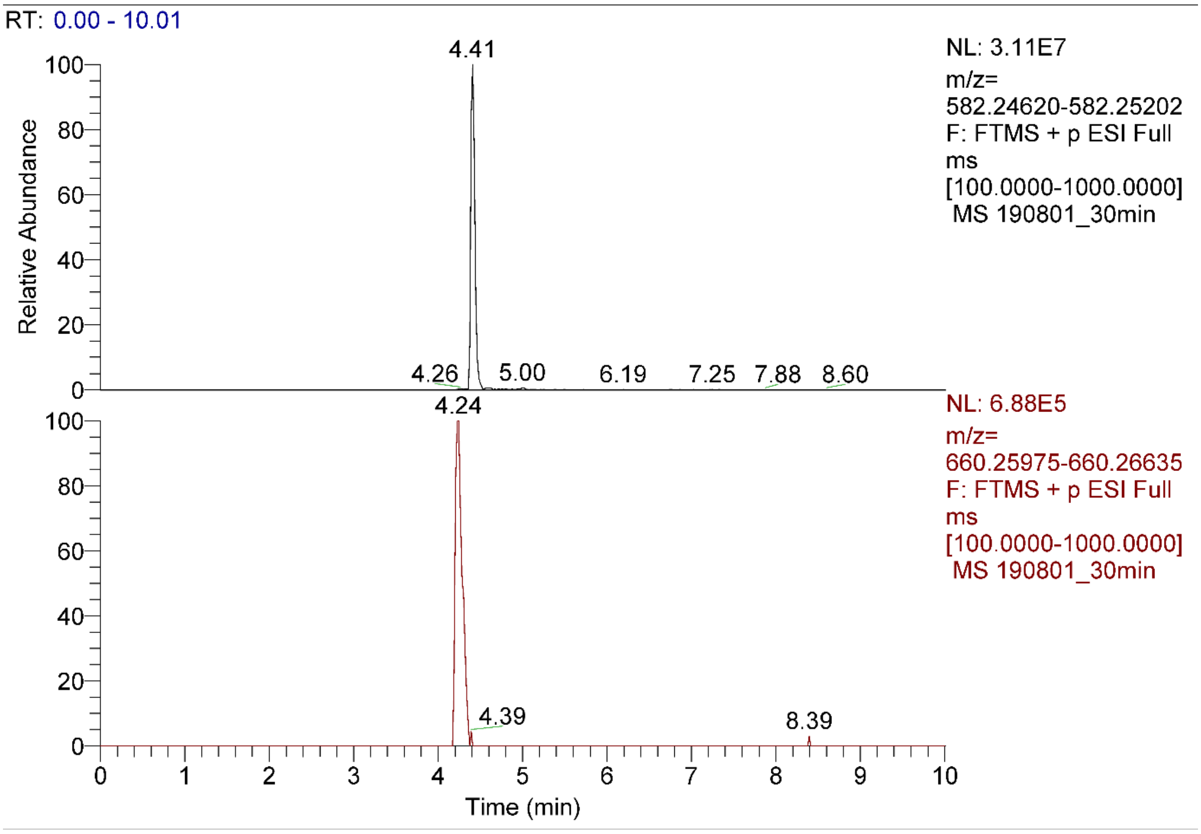

Figure S2. Time course HRMS analysis: **Figure S1-(c)** mixture.

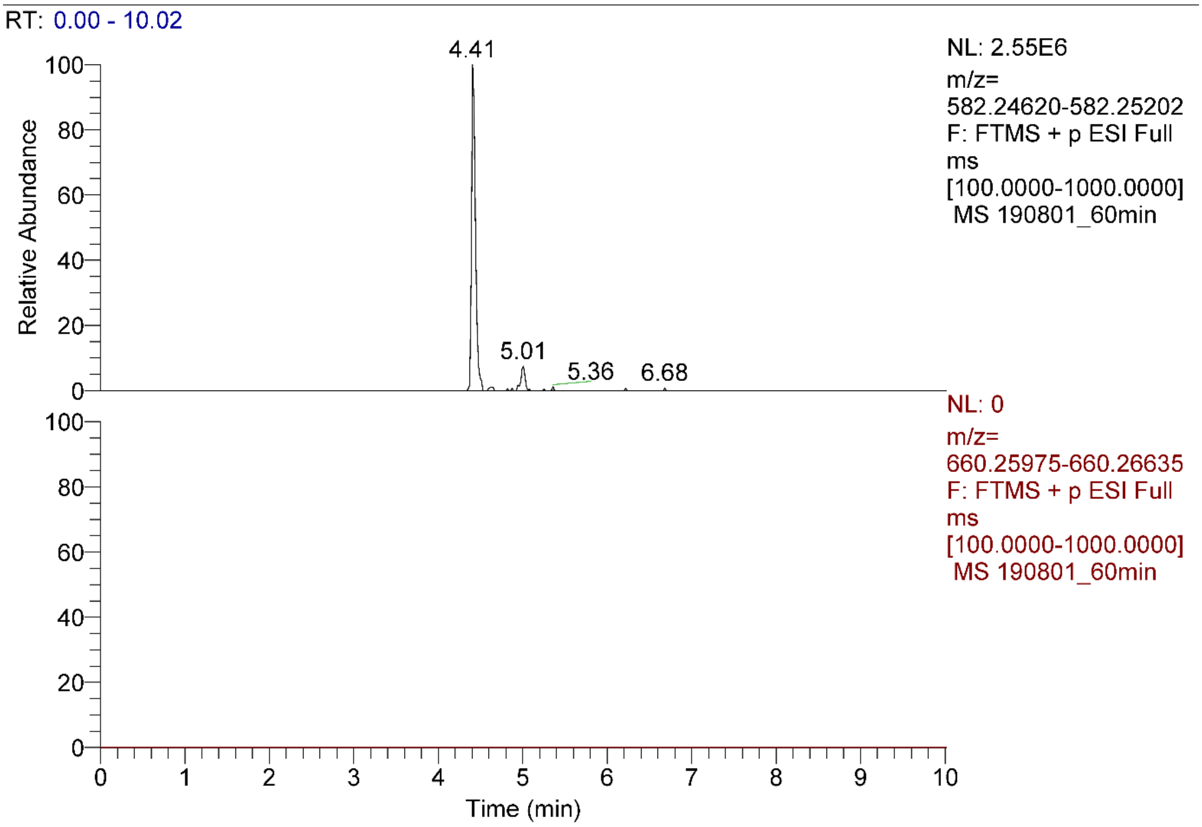

Figure S3. Time course HRMS analysis: **Figure S1-(d)** mixture.

The mixture of **Figure S1-(b)** was diluted with buffer and detected after 30 min (**Figure S2**) and 60 min (**Figure S3**), respectively. BME adduct was detected in **Figure S2** but not in **Figure S3**, which demonstrates the reversibility of the adducted compound over the time course. Each retention time of **13h** and BME – **13h** adduct mixture was 4.41 min and 4.24 min, respectively (Data not shown).

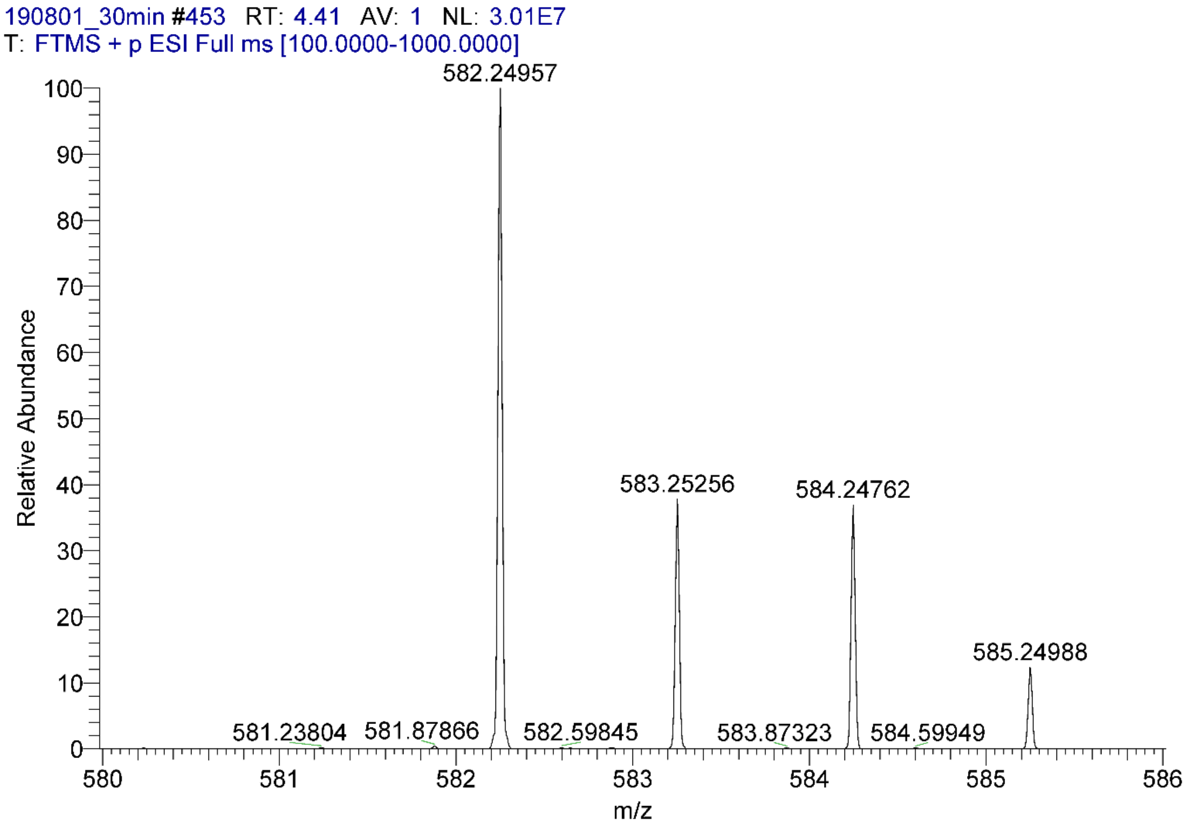

Figure S4. HRMS fragment pattern of **13h**.

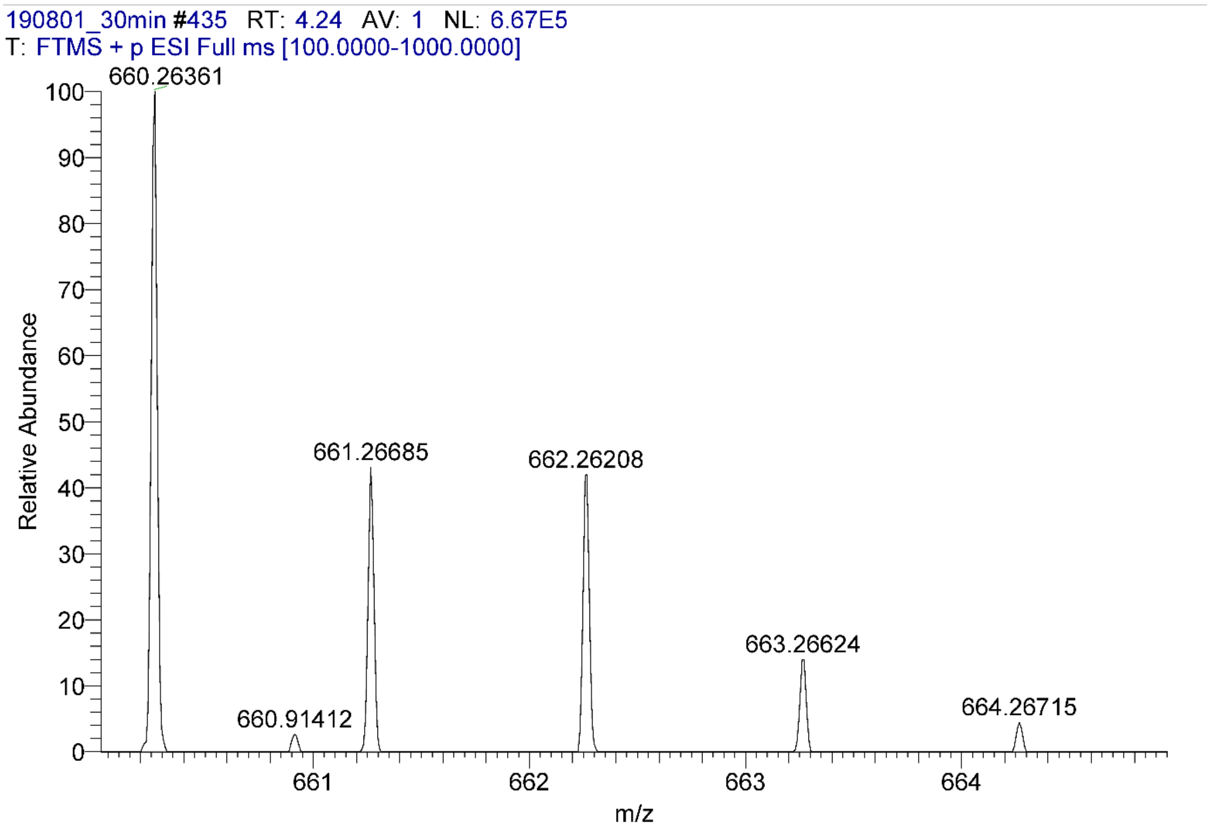

Figure S5. HRMS fragment pattern of **13h** with BME adduct.

2. NMR spectral data

All the NMR data was described in order of ^{1}H-NMR (600 MHz) and ^{13}C-NMR (150 MHz).

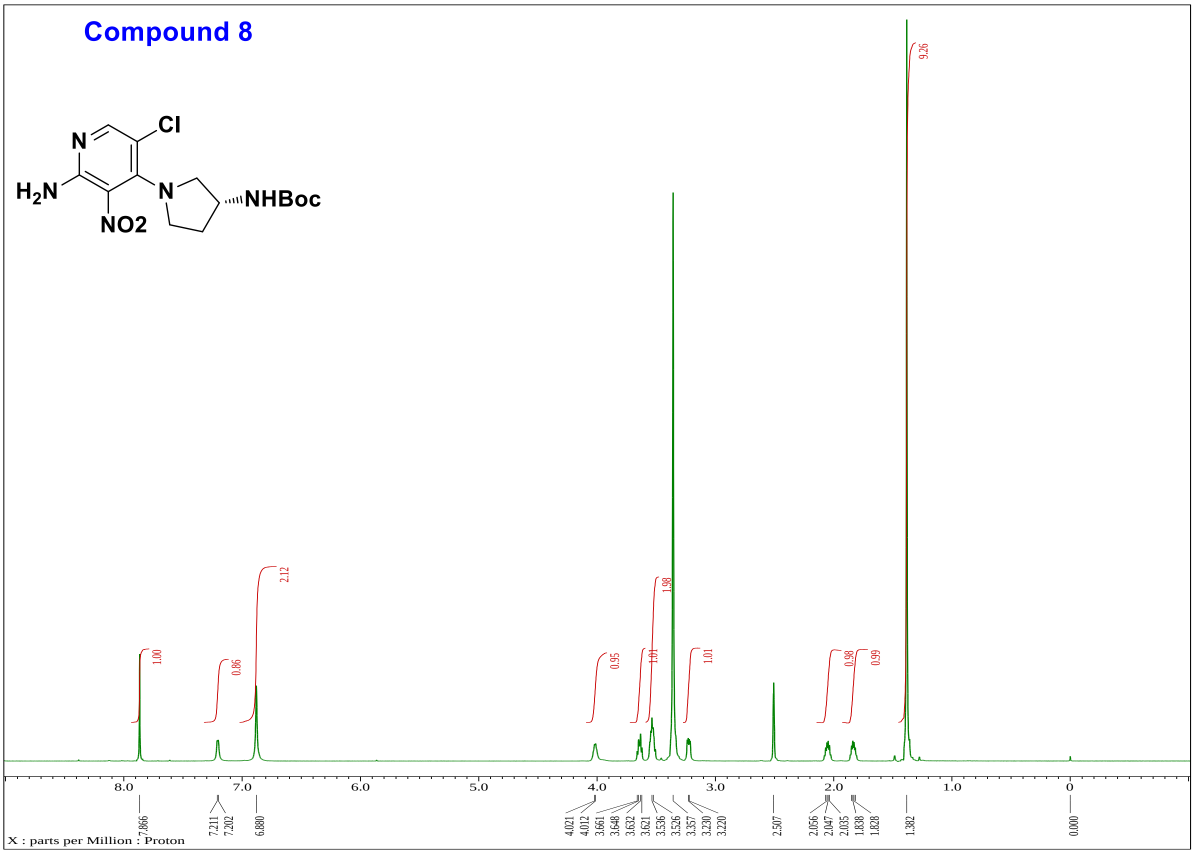
Compound 8
Cl
N
H2N
N
NHBoc
NO2
1.00
0.86
2.12
0.95
1.01
1.98
1.01
0.98
0.99
9.26
8.0
7.0
6.0
5.0
4.0
3.0
2.0
1.0
0
7.866
7.211
7.202
6.880
4.021
4.012
3.661
3.648
3.632
3.621
3.536
3.526
3.357
3.230
3.220
2.507
2.056
2.047
2.035
1.838
1.828
1.382
0.000
X : parts per Million : Proton

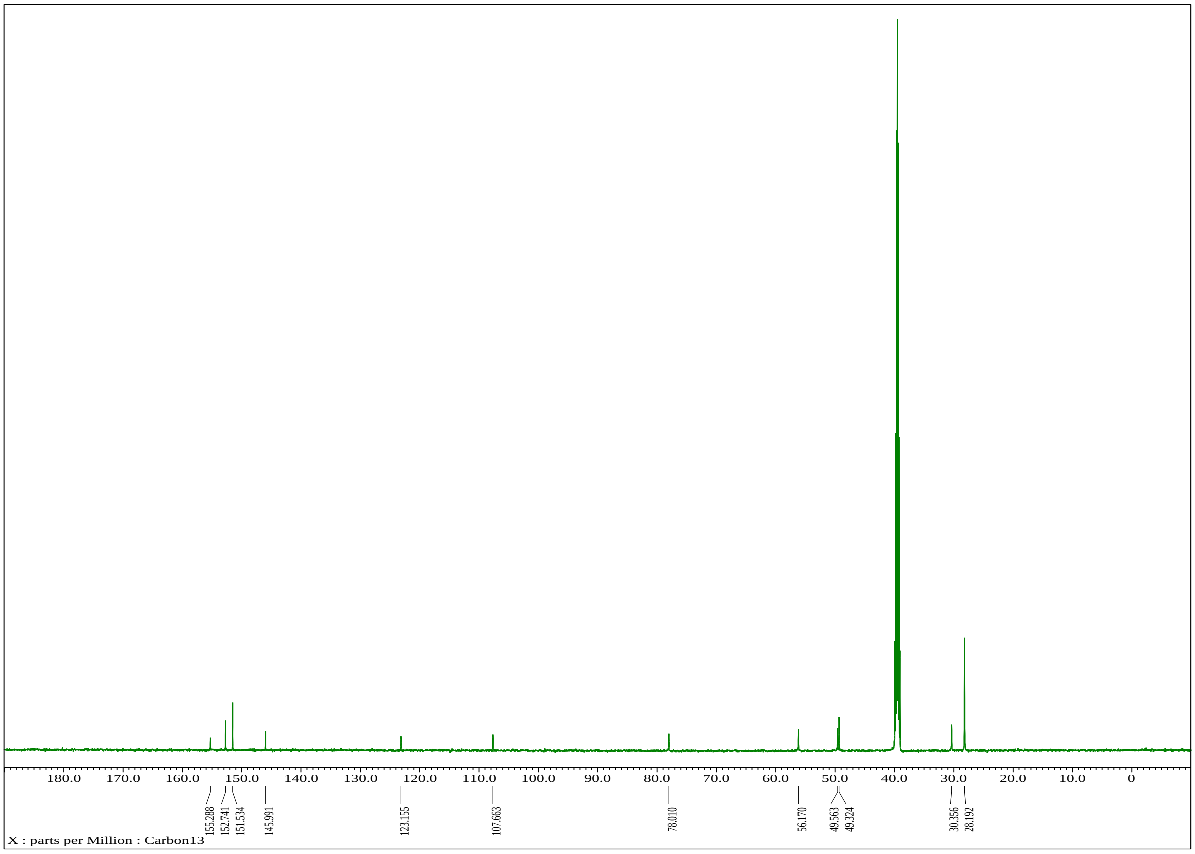
180.0
170.0
160.0
150.0
140.0
130.0
120.0
110.0
100.0
90.0
80.0
70.0
60.0
50.0
40.0
30.0
20.0
10.0
0
155.288
152.741
151.534
145.991
123.155
107.663
78.010
56.170
49.563
49.324
30.356
28.192
X : parts per Million : Carbon13

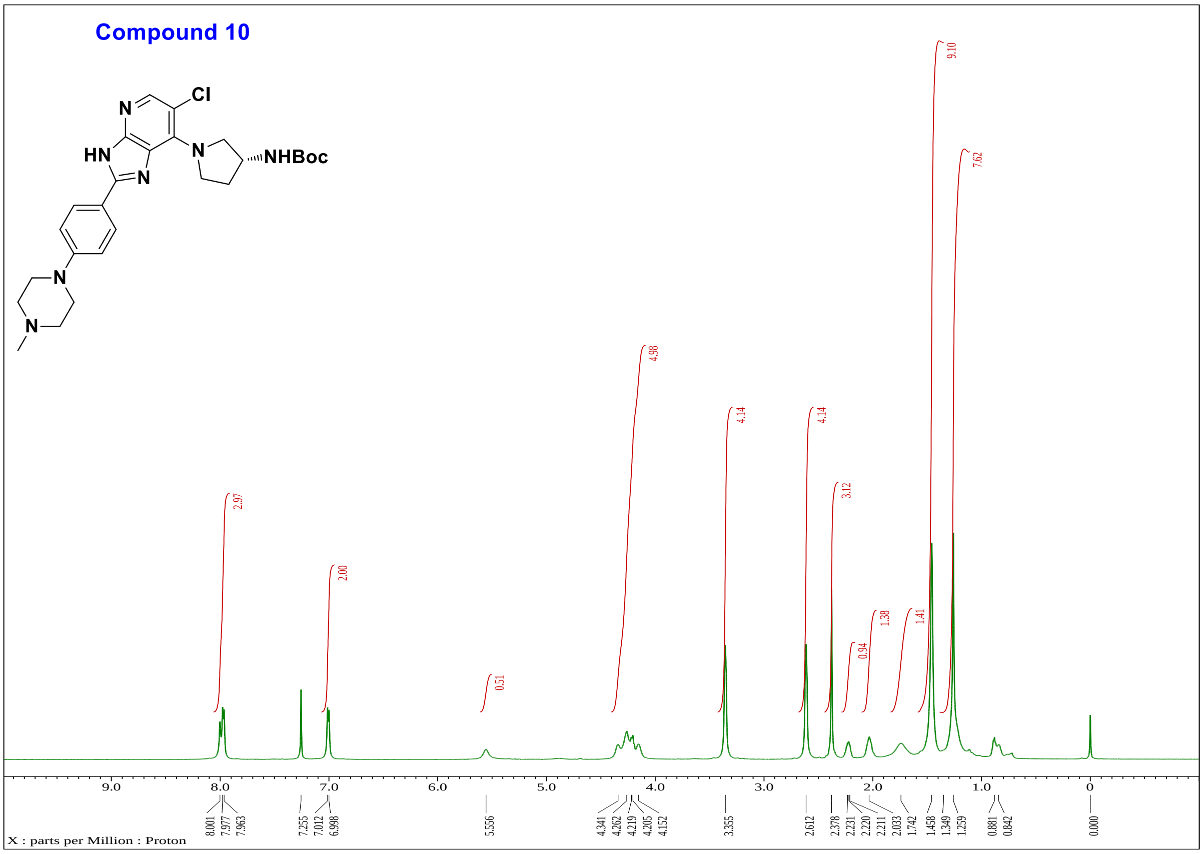
Compound 10
Cl
N
HN
N
N
NHBoc
N
N
2.97
2.00
0.51
4.98
4.14
4.14
3.12
0.94
1.38
1.41
9.10
7.62
9.0
8.0
7.0
6.0
5.0
4.0
3.0
2.0
1.0
0
8.001
7.977
7.963
7.255
7.012
6.998
5.556
4.341
4.262
4.219
4.205
4.152
3.355
2.612
2.378
2.231
2.220
2.211
2.033
1.742
1.458
1.349
1.259
0.881
0.842
0.000
X : parts per Million : Proton

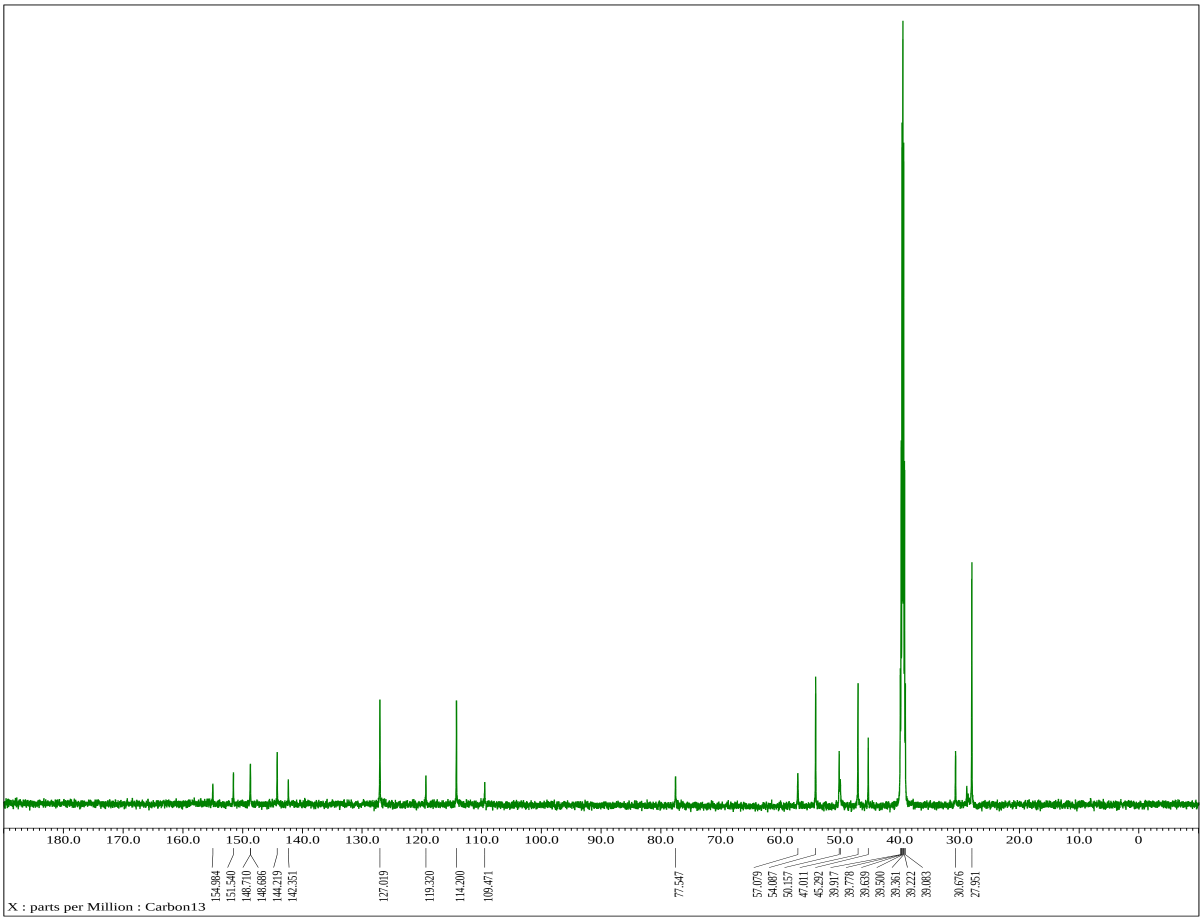
180.0 170.0 160.0 150.0 140.0 130.0 120.0 110.0 100.0 90.0 80.0 70.0 60.0 50.0 40.0 30.0 20.0 10.0 0
154.984
151.540
148.710
148.686
144.219
142.351
127.019
119.320
114.200
109.471
77.547
57.079
54.087
50.157
47.011
45.292
39.917
39.778
39.639
39.500
39.361
39.222
39.083
30.676
27.951
X : parts per Million : Carbon13

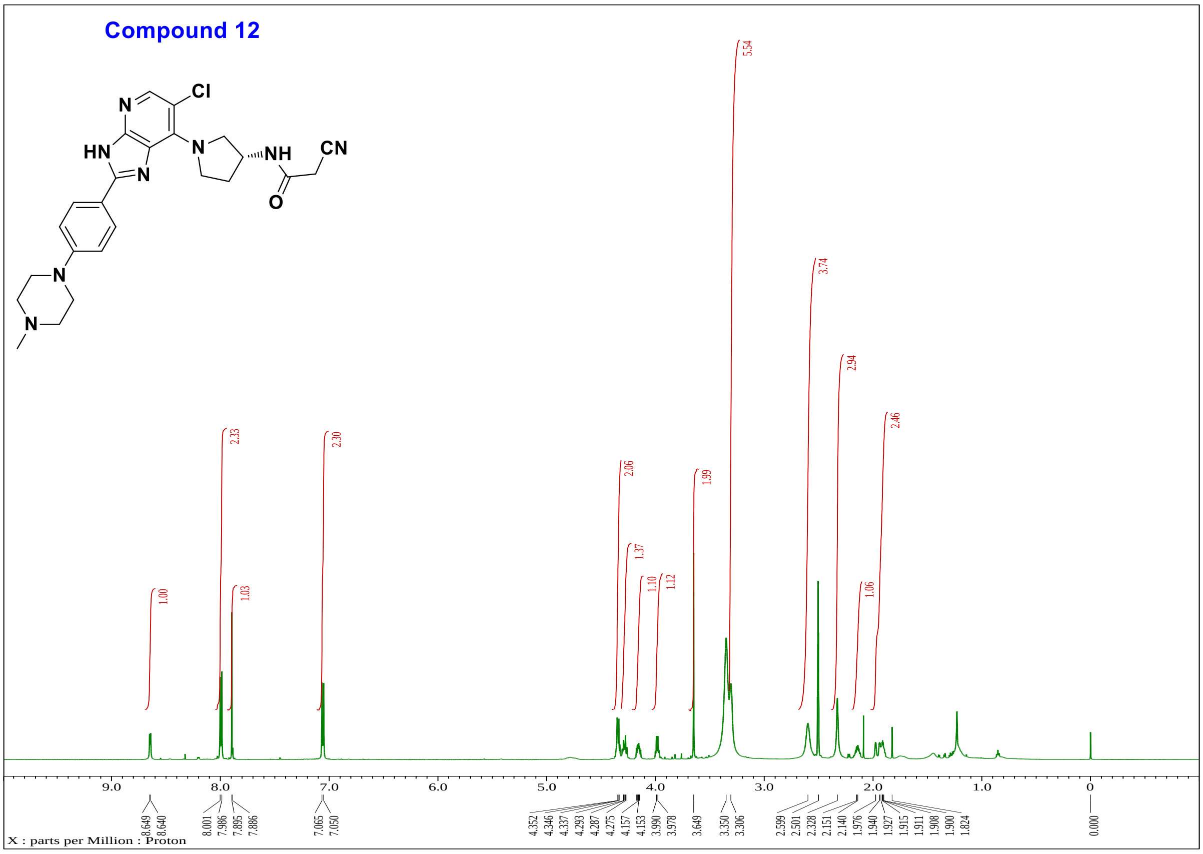
Compound 12
X : parts per Million : Proton

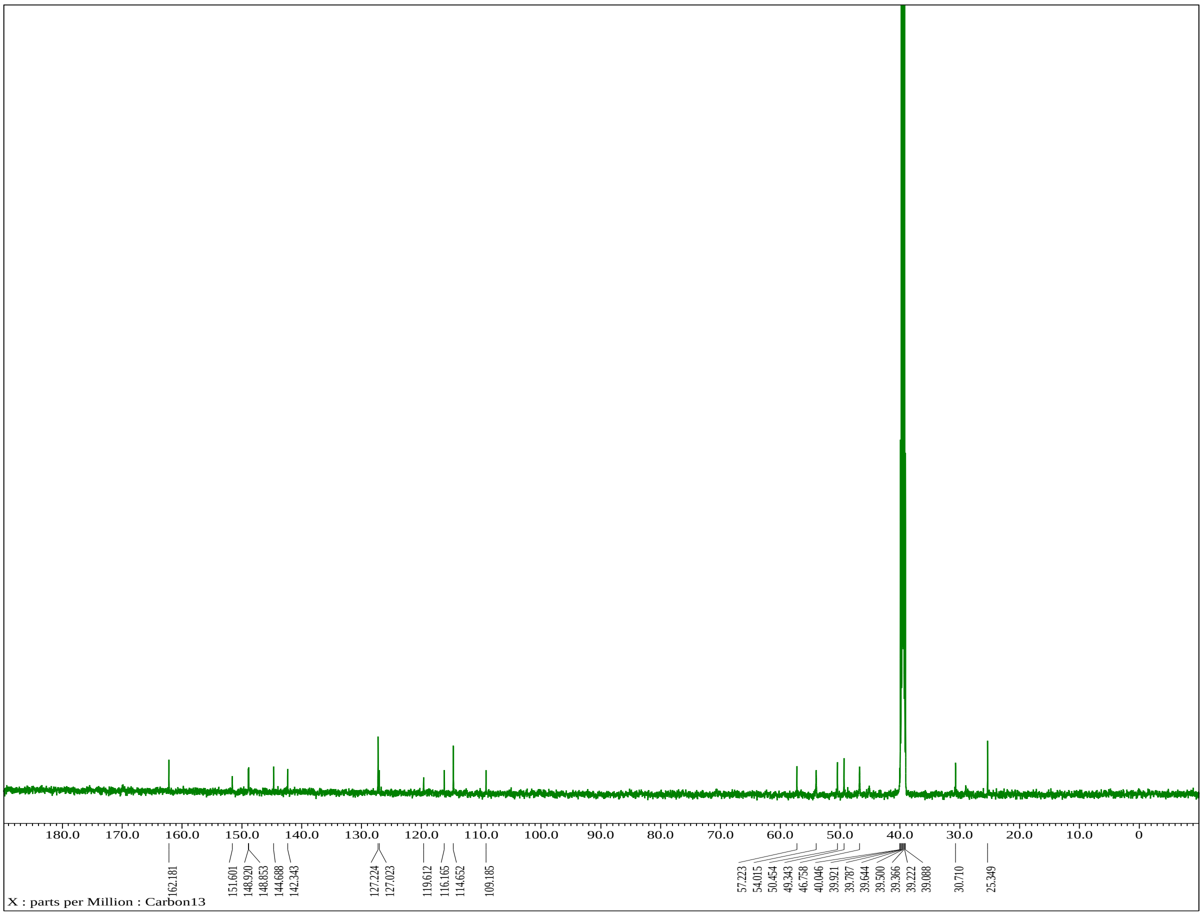
180.0 170.0 160.0 150.0 140.0 130.0 120.0 110.0 100.0 90.0 80.0 70.0 60.0 50.0 40.0 30.0 20.0 10.0 0
162.181
151.601 148.920 148.853 144.688 142.343
127.224 127.023
119.612 116.165 114.652
109.185
57.223 54.015 50.454 49.343 46.758 40.046 39.921 39.787 39.644 39.500 39.366 39.222 39.088
30.710
25.349
X : parts per Million : Carbon13

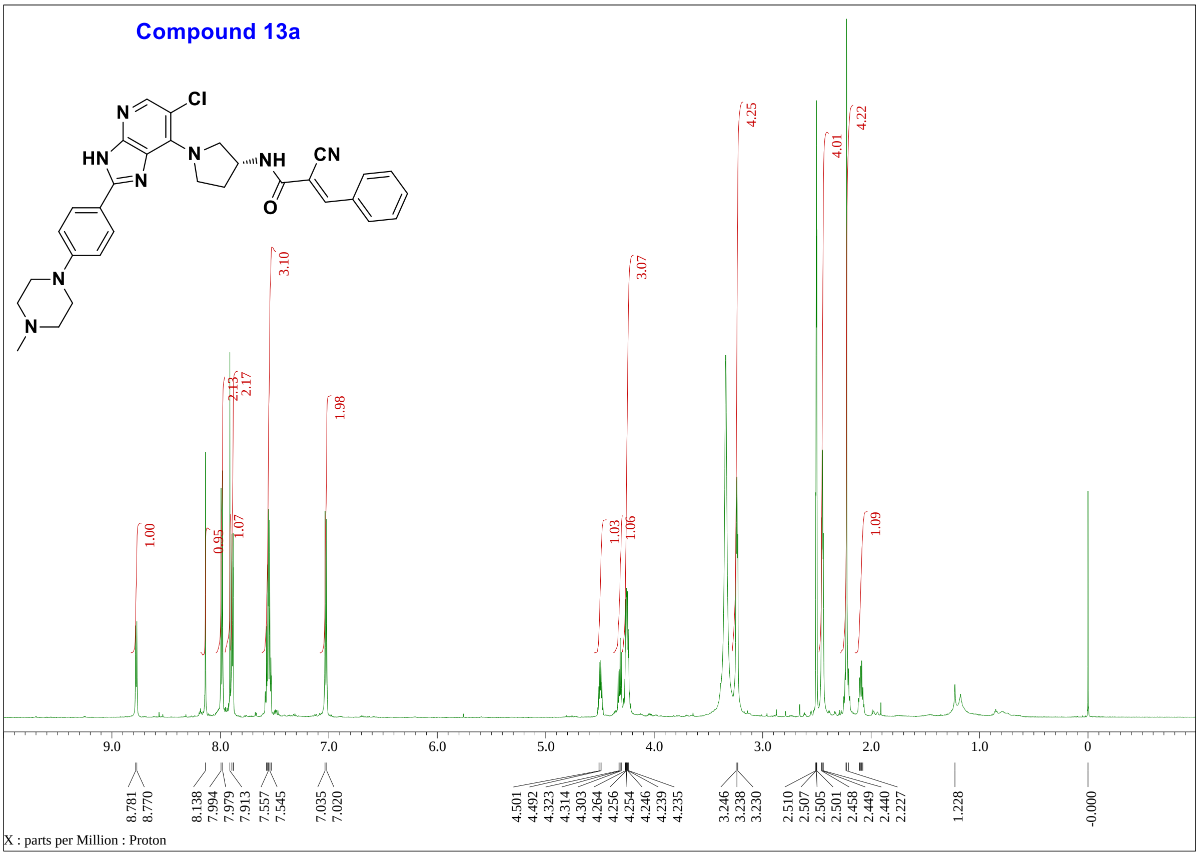
Compound 13a
Cl
N
HN
N
N
NH
CN
O
N
N
1.00
0.95
2.13
2.17
1.07
3.10
1.98
1.03
1.06
3.07
4.25
4.01
4.22
1.09
9.0
8.0
7.0
6.0
5.0
4.0
3.0
2.0
1.0
0
8.781
8.770
8.138
7.994
7.979
7.913
7.557
7.545
7.035
7.020
4.501
4.492
4.323
4.314
4.303
4.264
4.256
4.254
4.246
4.239
4.235
3.246
3.238
3.230
2.510
2.507
2.505
2.501
2.458
2.449
2.440
2.227
1.228
-0.000
X : parts per Million : Proton

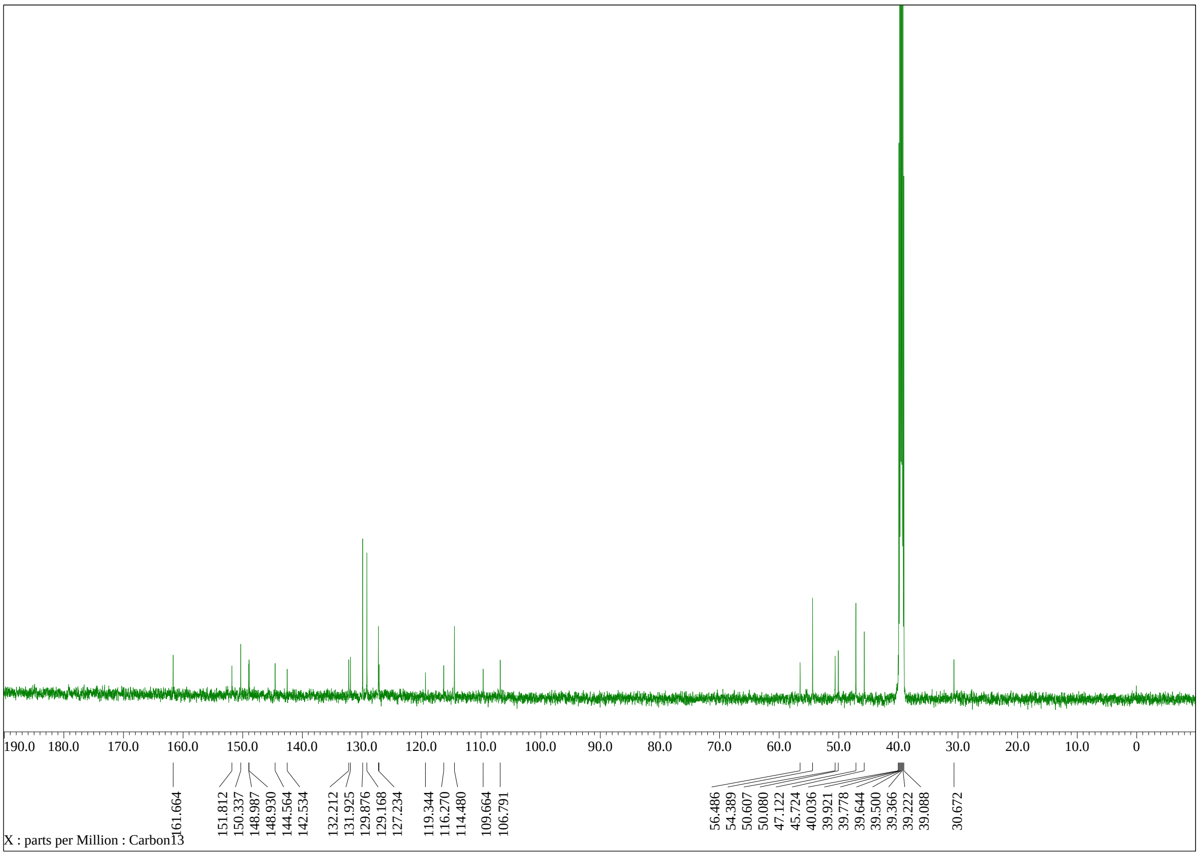
190.0
180.0
170.0
160.0
150.0
140.0
130.0
120.0
110.0
100.0
90.0
80.0
70.0
60.0
50.0
40.0
30.0
20.0
10.0
0
161.664
151.812
150.337
148.987
148.930
144.564
142.534
132.212
131.925
129.876
129.168
127.234
119.344
116.270
114.480
109.664
106.791
56.486
54.389
50.607
50.080
47.122
45.724
40.036
39.921
39.778
39.644
39.500
39.366
39.222
39.088
30.672
X : parts per Million : Carbon13

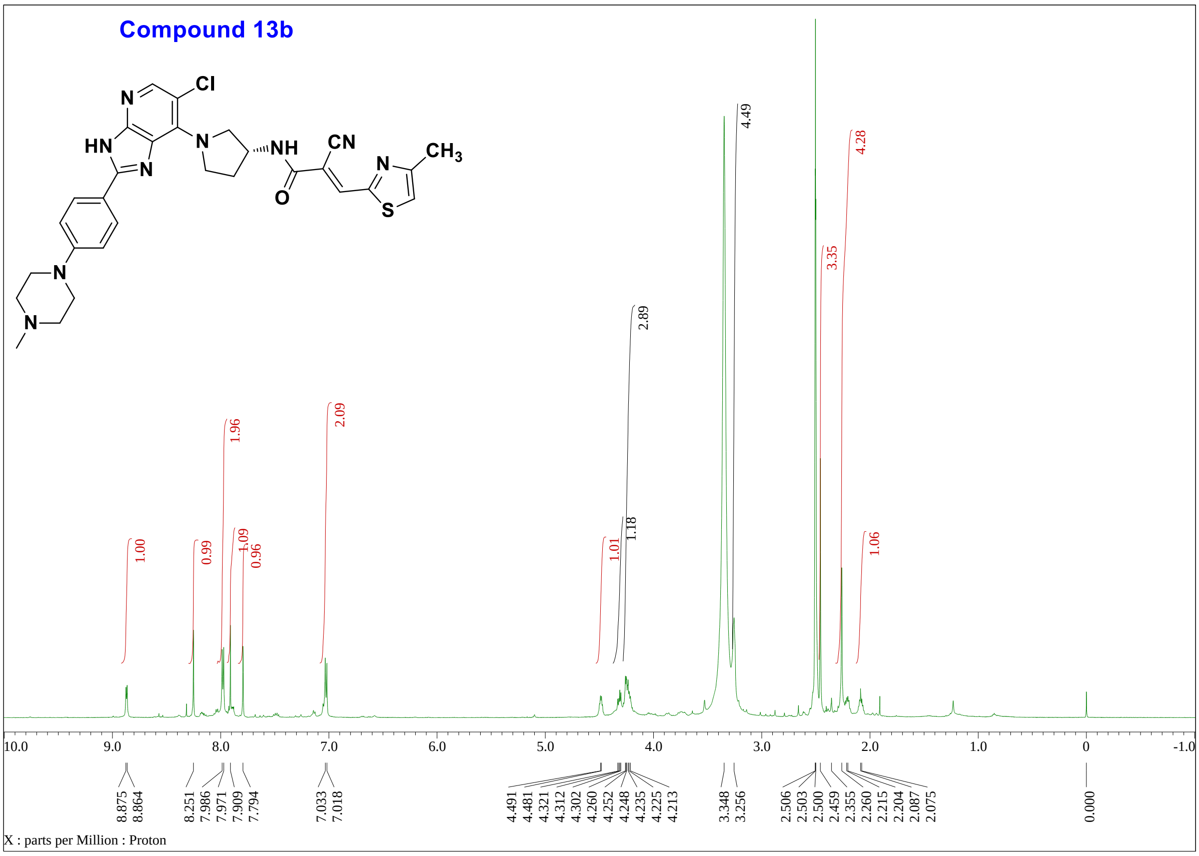
Compound 13b
CH3
CN
NH
HN
N
Cl
S
O
1.00
0.99
1.96
1.09
0.96
2.09
1.01
1.18
2.89
4.49
3.35
4.28
1.06
10.0 9.0 8.0 7.0 6.0 5.0 4.0 3.0 2.0 1.0 0 -1.0
8.875
8.864
8.251
7.986
7.971
7.909
7.794
7.033
7.018
4.491
4.481
4.321
4.312
4.302
4.260
4.252
4.248
4.235
4.225
4.213
3.348
3.256
2.506
2.503
2.500
2.459
2.355
2.260
2.215
2.204
2.087
2.075
0.000
X : parts per Million : Proton

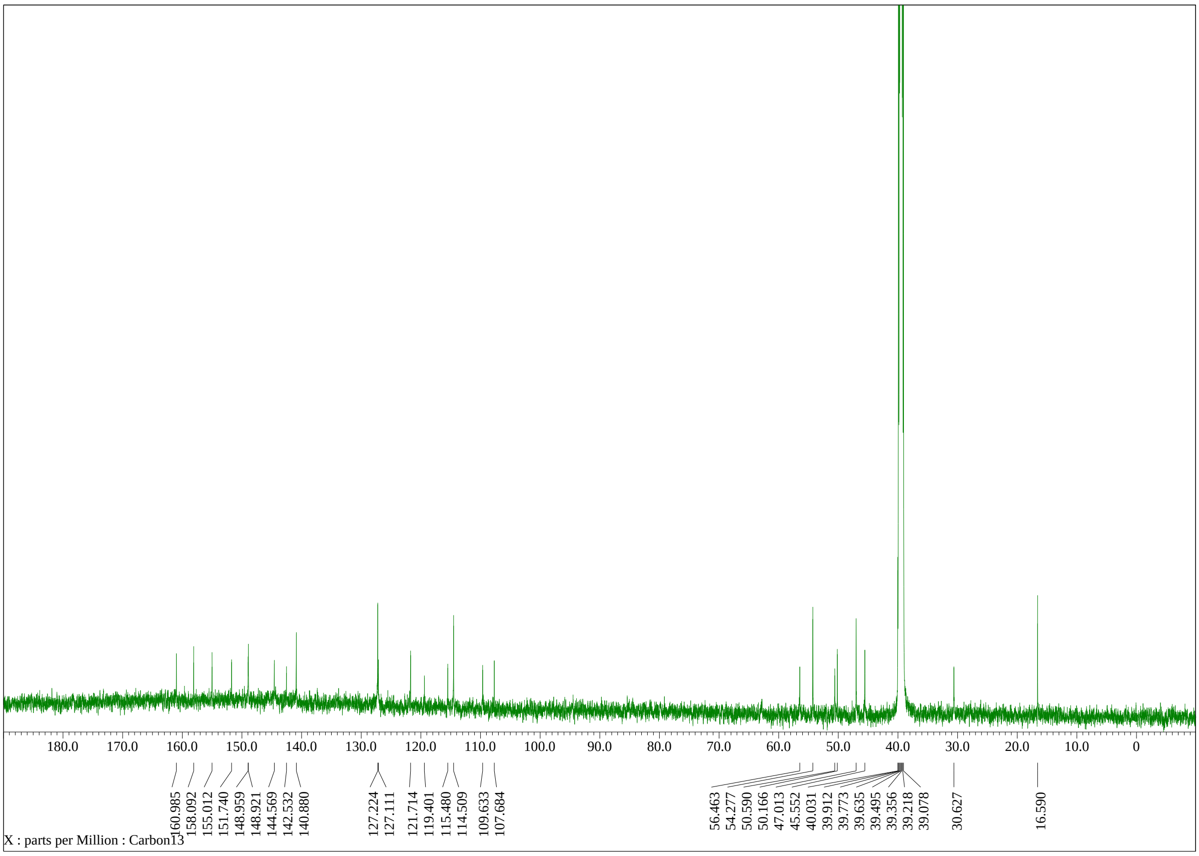
180.0 170.0 160.0 150.0 140.0 130.0 120.0 110.0 100.0 90.0 80.0 70.0 60.0 50.0 40.0 30.0 20.0 10.0 0
160.985
158.092
155.012
151.740
148.959
148.921
144.569
142.532
140.880
127.224
127.111
121.714
119.401
115.480
114.509
109.633
107.684
56.463
54.277
50.590
50.166
47.013
45.552
40.031
39.912
39.773
39.635
39.495
39.356
39.218
39.078
30.627
16.590
X : parts per Million : Carbon13

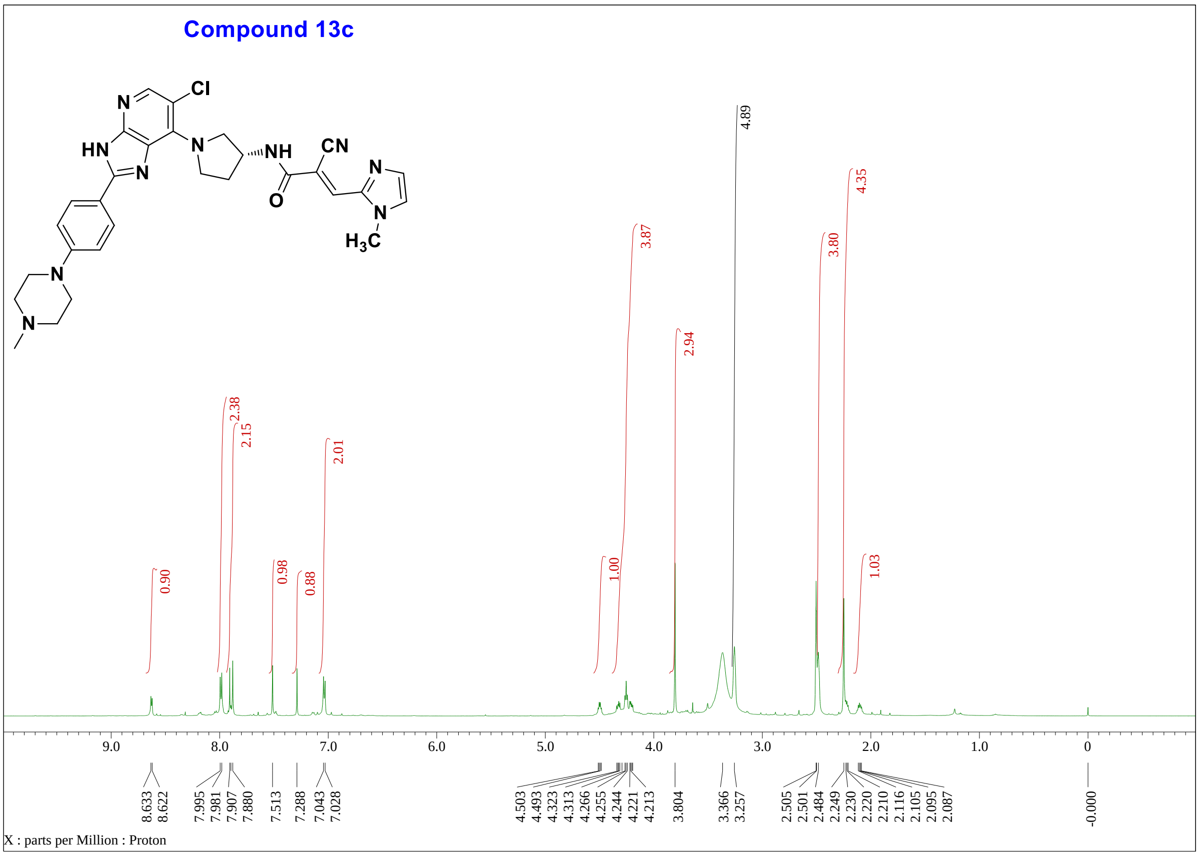
Compound 13c
N
Cl
HN
N
N
NH
CN
N
O
N
H_3C
N
N
0.90
2.38
2.15
0.98
0.88
2.01
1.00
3.87
2.94
4.89
3.80
4.35
1.03
9.0
8.0
7.0
6.0
5.0
4.0
3.0
2.0
1.0
0
8.633
8.622
7.995
7.981
7.907
7.880
7.513
7.288
7.043
7.028
4.503
4.493
4.323
4.313
4.266
4.255
4.244
4.221
4.213
3.804
3.366
3.257
2.505
2.501
2.484
2.249
2.230
2.220
2.210
2.116
2.105
2.095
2.087
-0.000
X : parts per Million : Proton

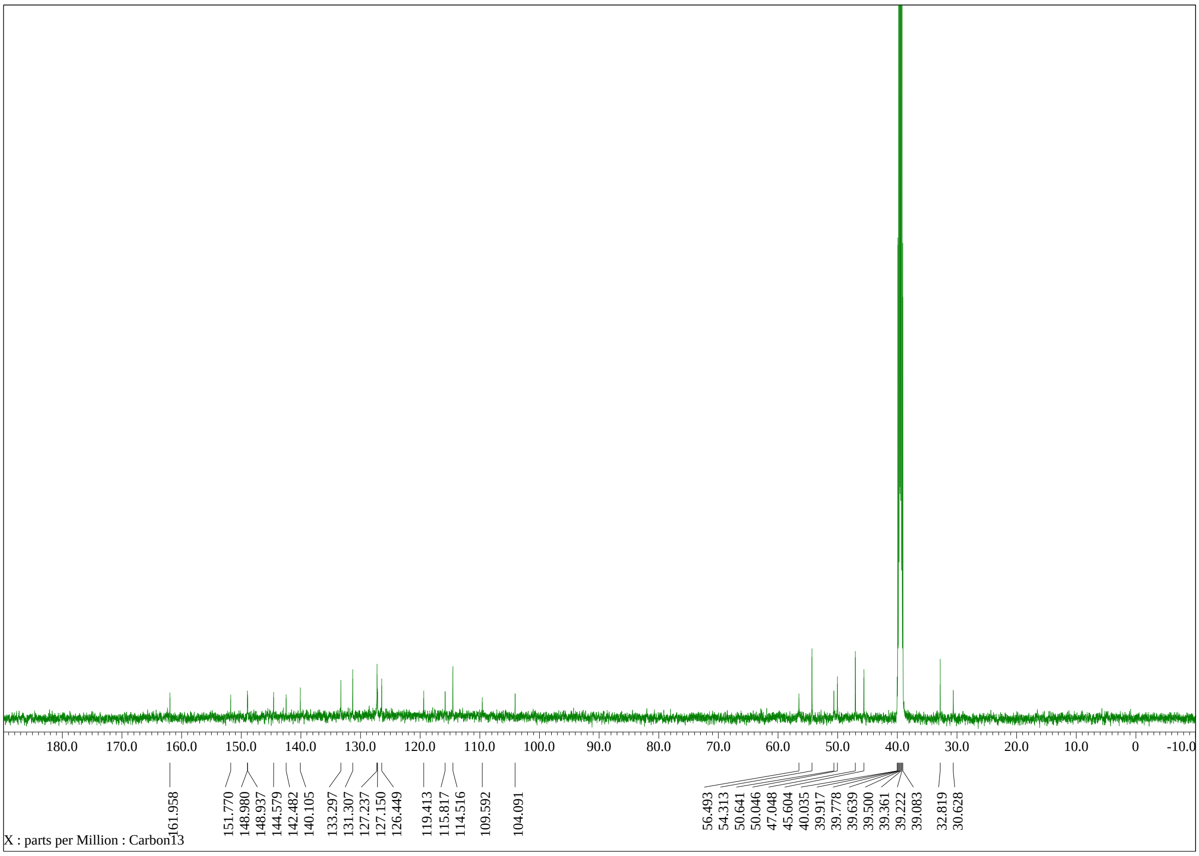
180.0
170.0
160.0
150.0
140.0
130.0
120.0
110.0
100.0
90.0
80.0
70.0
60.0
50.0
40.0
30.0
20.0
10.0
0
-10.0
161.958
151.770
148.980
148.937
144.579
142.482
140.105
133.297
131.307
127.237
127.150
126.449
119.413
115.817
114.516
109.592
104.091
56.493
54.313
50.641
50.046
47.048
45.604
40.035
39.917
39.778
39.639
39.500
39.361
39.222
39.083
32.819
30.628
X : parts per Million : Carbon13

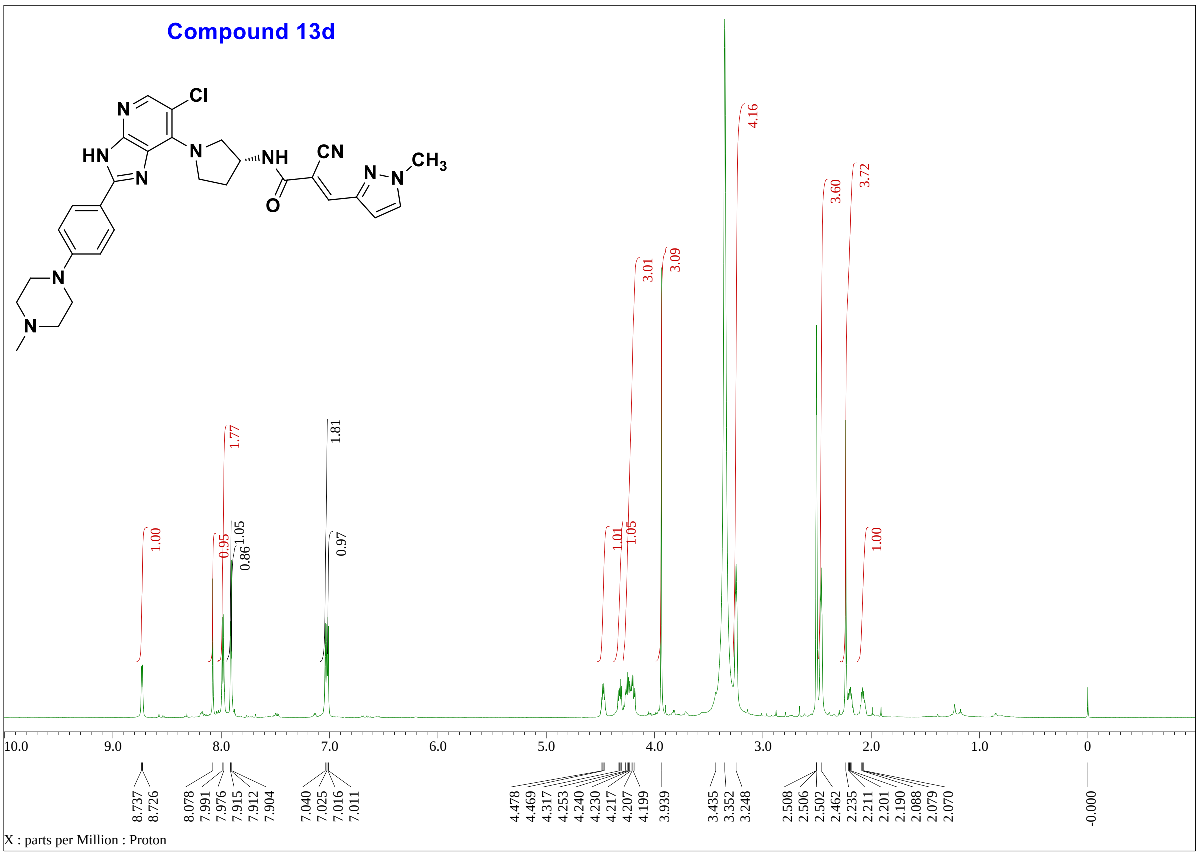
Compound 13d
Cl
N
HN
N
N
NH
CN
O
N
N
CH3
N
N
1.00
0.95
1.77
0.86
1.05
1.81
0.97
1.01
1.05
3.01
3.09
4.16
3.60
3.72
1.00
10.0
9.0
8.0
7.0
6.0
5.0
4.0
3.0
2.0
1.0
0
8.737
8.726
8.078
7.991
7.976
7.915
7.912
7.904
7.040
7.025
7.016
7.011
4.478
4.469
4.317
4.253
4.240
4.230
4.217
4.207
4.199
3.939
3.435
3.352
3.248
2.508
2.506
2.502
2.462
2.235
2.211
2.201
2.190
2.088
2.079
2.070
-0.000
X : parts per Million : Proton

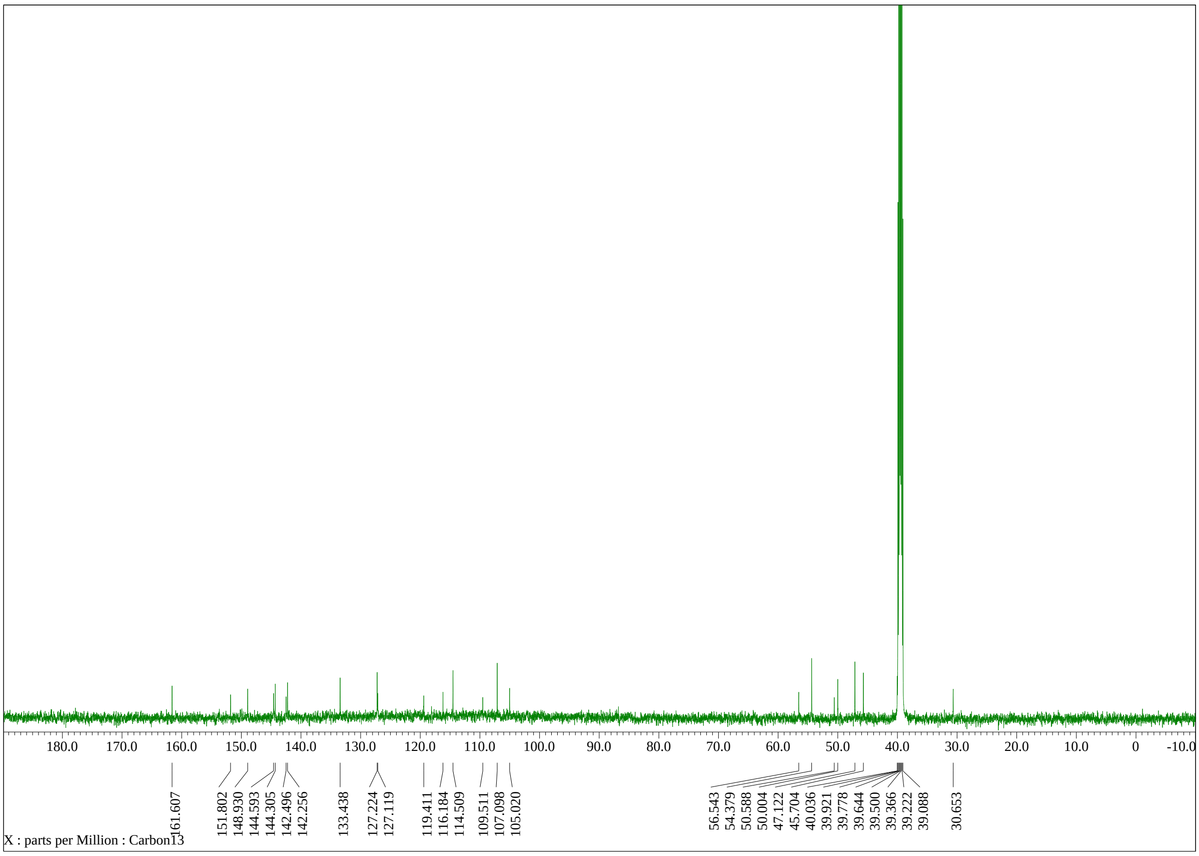
180.0
170.0
160.0
150.0
140.0
130.0
120.0
110.0
100.0
90.0
80.0
70.0
60.0
50.0
40.0
30.0
20.0
10.0
0
-10.0
161.607
151.802
148.930
144.593
144.305
142.496
142.256
133.438
127.224
127.119
119.411
116.184
114.509
109.511
107.098
105.020
56.543
54.379
50.588
50.004
47.122
45.704
40.036
39.921
39.778
39.644
39.500
39.366
39.222
39.088
30.653
X : parts per Million : Carbon13

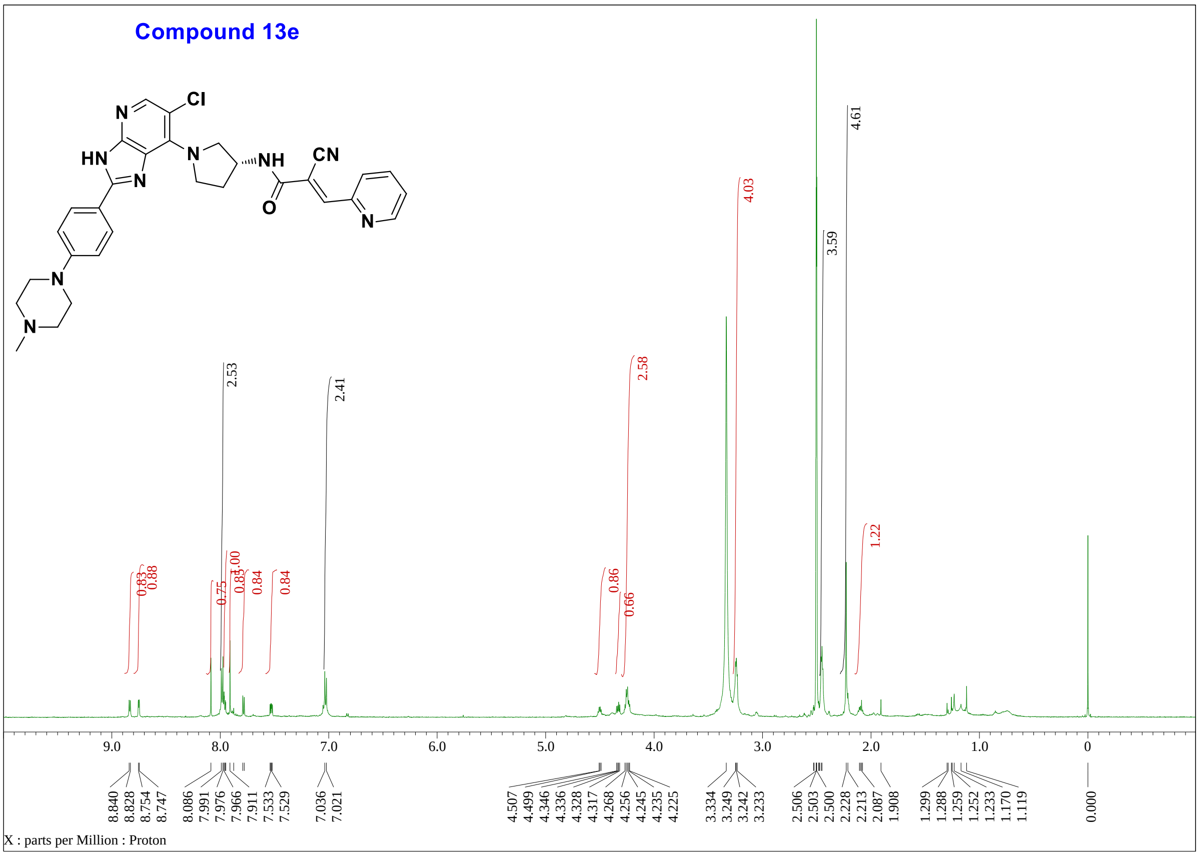
Compound 13e
Cl
N
HN
N
N
NH
CN
O
N
N
N
2.53
2.41
4.03
3.59
4.61
2.58
1.22
0.83
0.88
0.75
1.00
0.85
0.84
0.84
0.86
0.66
9.0
8.0
7.0
6.0
5.0
4.0
3.0
2.0
1.0
0
8.840
8.828
8.754
8.747
8.086
7.991
7.976
7.966
7.911
7.533
7.529
7.036
7.021
4.507
4.499
4.346
4.336
4.328
4.317
4.268
4.256
4.245
4.235
4.225
3.334
3.249
3.242
3.233
2.506
2.503
2.500
2.228
2.213
2.087
1.908
1.299
1.288
1.259
1.252
1.233
1.170
1.119
0.000
X : parts per Million : Proton

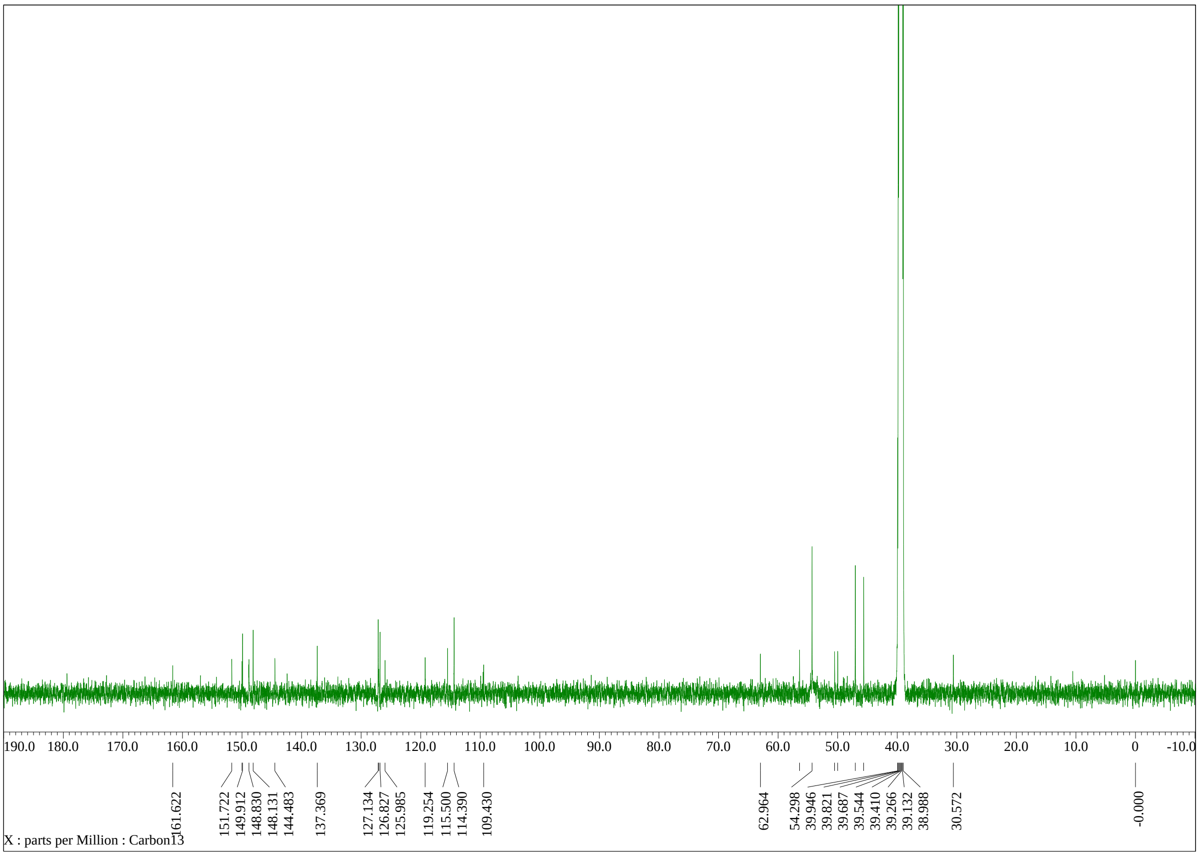
190.0
180.0
170.0
160.0
150.0
140.0
130.0
120.0
110.0
100.0
90.0
80.0
70.0
60.0
50.0
40.0
30.0
20.0
10.0
0
-10.0
161.622
151.722
149.912
148.830
148.131
144.483
137.369
127.134
126.827
125.985
119.254
115.500
114.390
109.430
62.964
54.298
39.946
39.821
39.687
39.544
39.410
39.266
39.132
38.988
30.572
-0.000
X : parts per Million : Carbon13

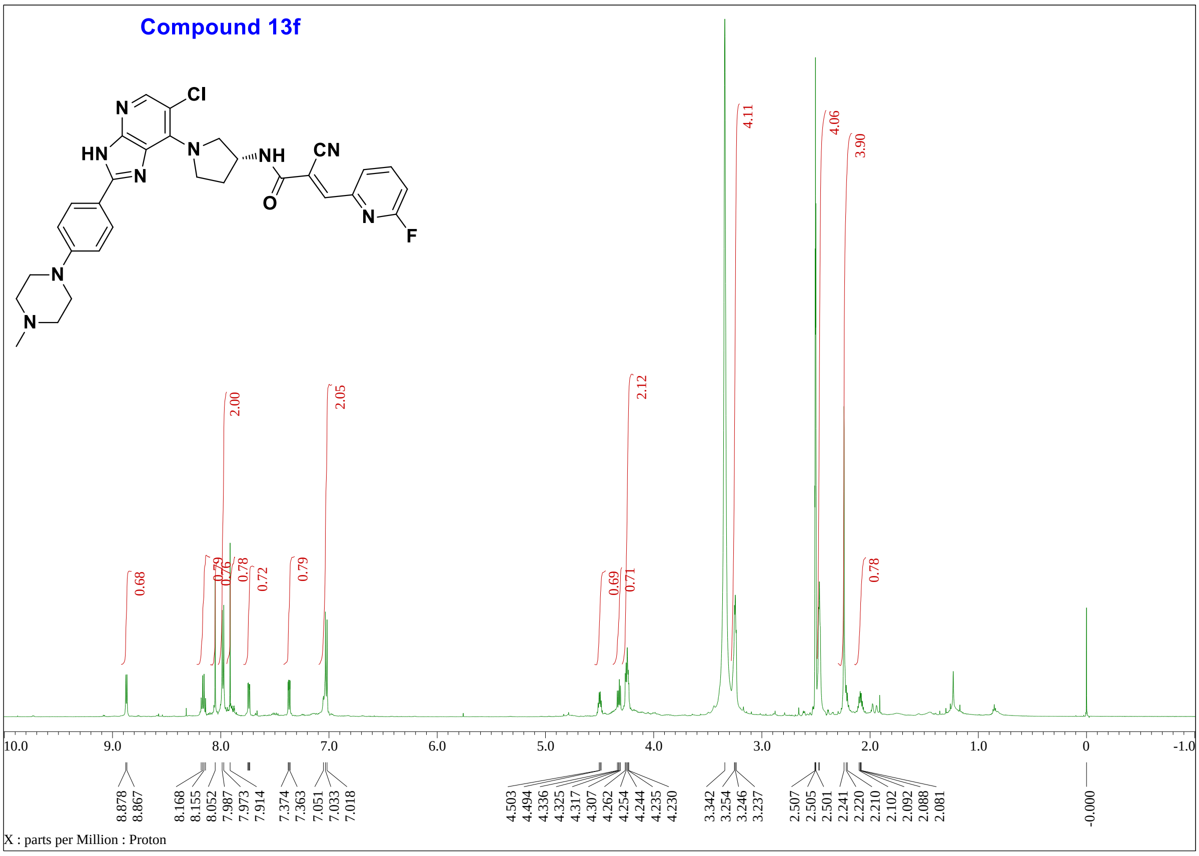

Compound 13f
X : parts per Million : Proton
10.0
9.0
8.0
7.0
6.0
5.0
4.0
3.0
2.0
1.0
0
-1.0

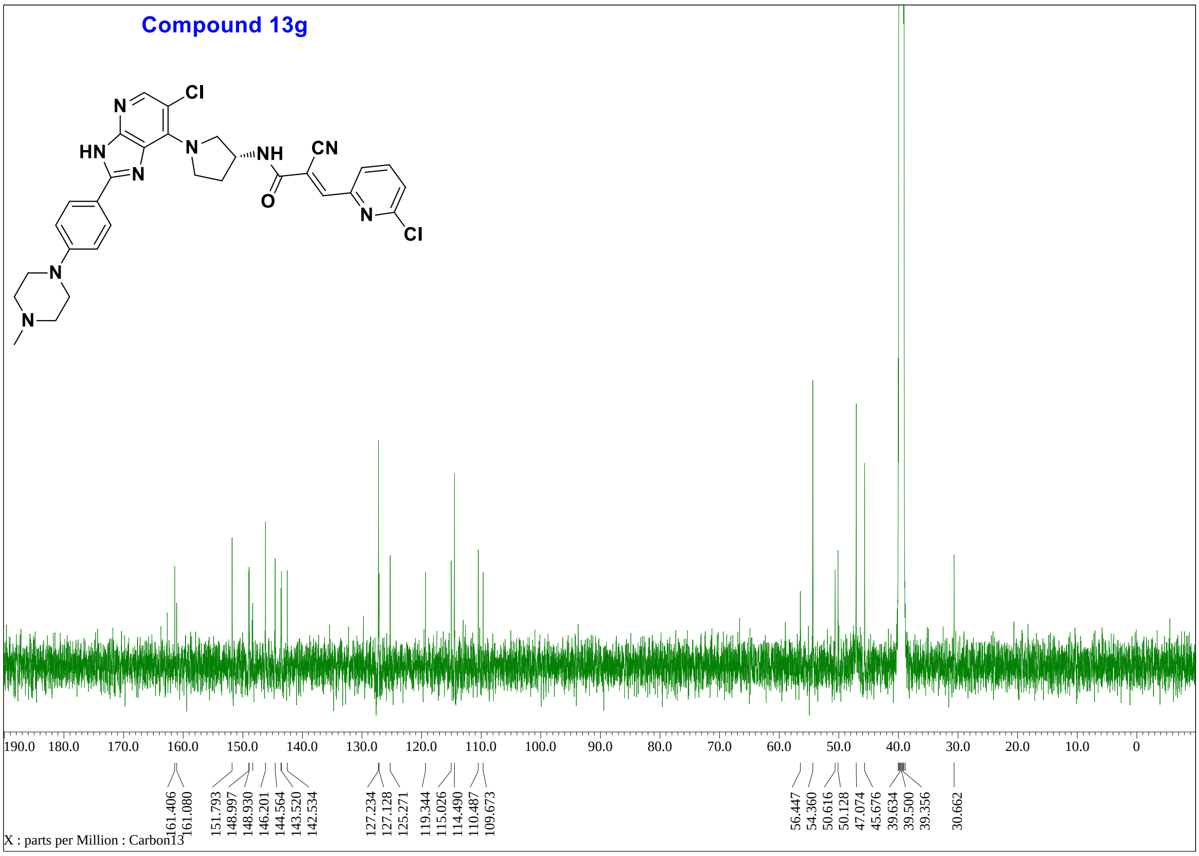
Compound 13g
Cl
N
HN
N
N
NH
CN
O
N
Cl
N
N
190.0 180.0 170.0 160.0 150.0 140.0 130.0 120.0 110.0 100.0 90.0 80.0 70.0 60.0 50.0 40.0 30.0 20.0 10.0 0
161.406
161.080
151.793
148.997
148.930
146.201
144.564
143.520
142.534
127.234
127.128
125.271
119.344
115.026
114.490
110.487
109.673
56.447
54.360
50.616
50.128
47.074
45.676
39.634
39.500
39.356
30.662
X : parts per Million : Carbon13

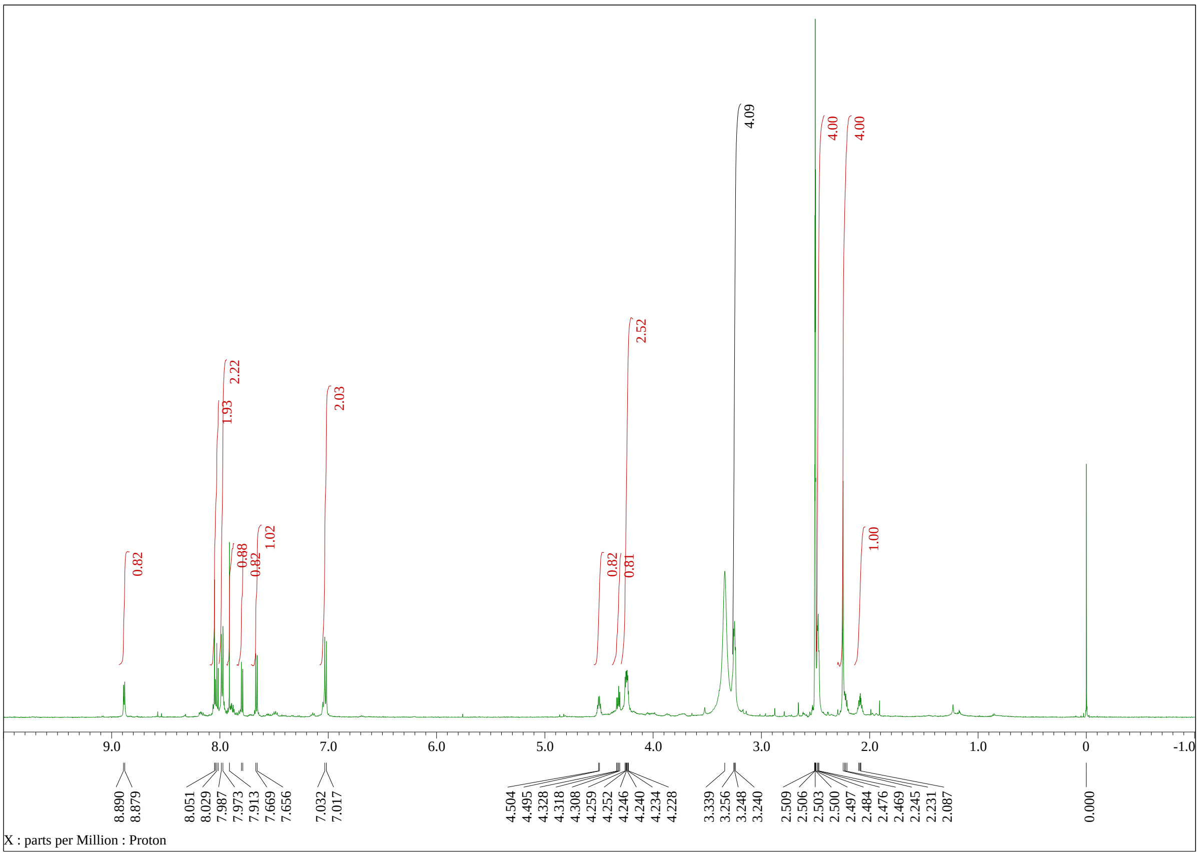
0.82
1.93
2.22
0.88
0.82
1.02
2.03
0.82
0.81
2.52
4.09
4.00
4.00
1.00
9.0 8.0 7.0 6.0 5.0 4.0 3.0 2.0 1.0 0 -1.0
8.890
8.879
8.051
8.029
7.987
7.973
7.913
7.669
7.656
7.032
7.017
4.504
4.495
4.328
4.318
4.308
4.259
4.252
4.246
4.240
4.234
4.228
3.339
3.256
3.248
3.240
2.509
2.506
2.503
2.500
2.497
2.484
2.476
2.469
2.245
2.231
2.087
0.000
X : parts per Million : Proton

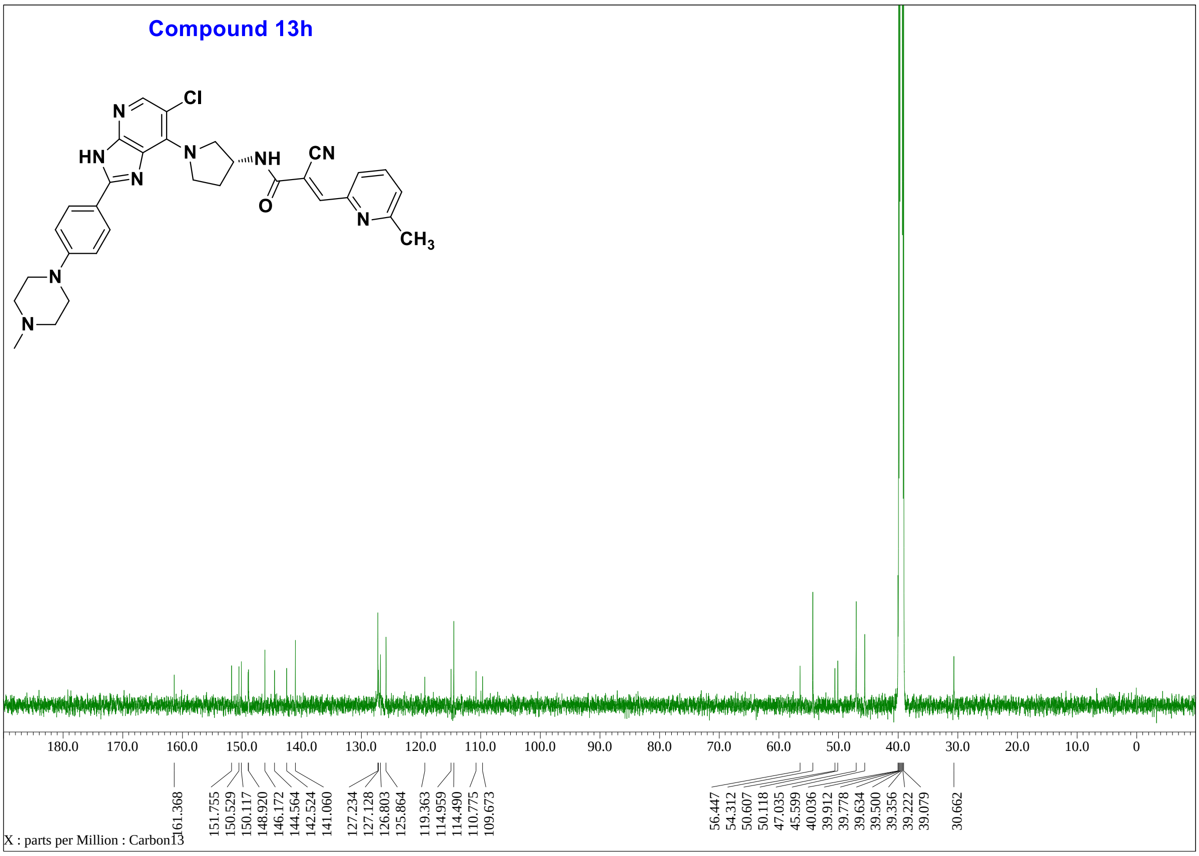
Compound 13h
Cl
N
HN
N
N
NH
CN
O
N
CH3
N
N
180.0 170.0 160.0 150.0 140.0 130.0 120.0 110.0 100.0 90.0 80.0 70.0 60.0 50.0 40.0 30.0 20.0 10.0 0
161.368
151.755
150.529
150.117
148.920
146.172
144.564
142.524
141.060
127.234
127.128
126.803
125.864
119.363
114.959
114.490
110.775
109.673
56.447
54.312
50.607
50.118
47.035
45.599
40.036
39.912
39.778
39.634
39.500
39.356
39.222
39.079
30.662
X : parts per Million : Carbon13

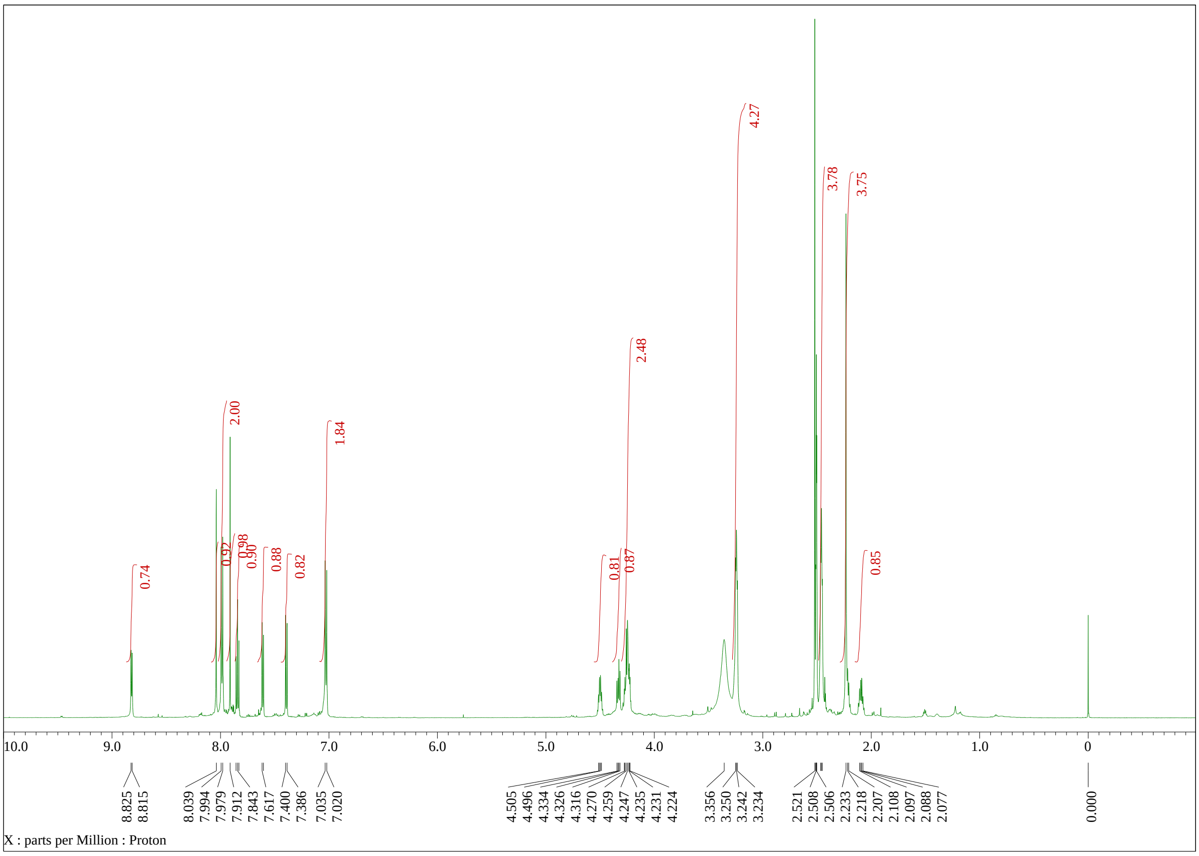
4.27
3.78
3.75
2.48
2.00
1.84
0.74
0.92
0.98
0.90
0.88
0.82
0.81
0.87
0.85
10.0 9.0 8.0 7.0 6.0 5.0 4.0 3.0 2.0 1.0 0
8.825
8.815
8.039
7.994
7.979
7.912
7.843
7.617
7.400
7.386
7.035
7.020
4.505
4.496
4.334
4.326
4.316
4.270
4.259
4.247
4.235
4.231
4.224
3.356
3.250
3.242
3.234
2.521
2.508
2.506
2.233
2.218
2.207
2.108
2.097
2.088
2.077
0.000
X : parts per Million : Proton

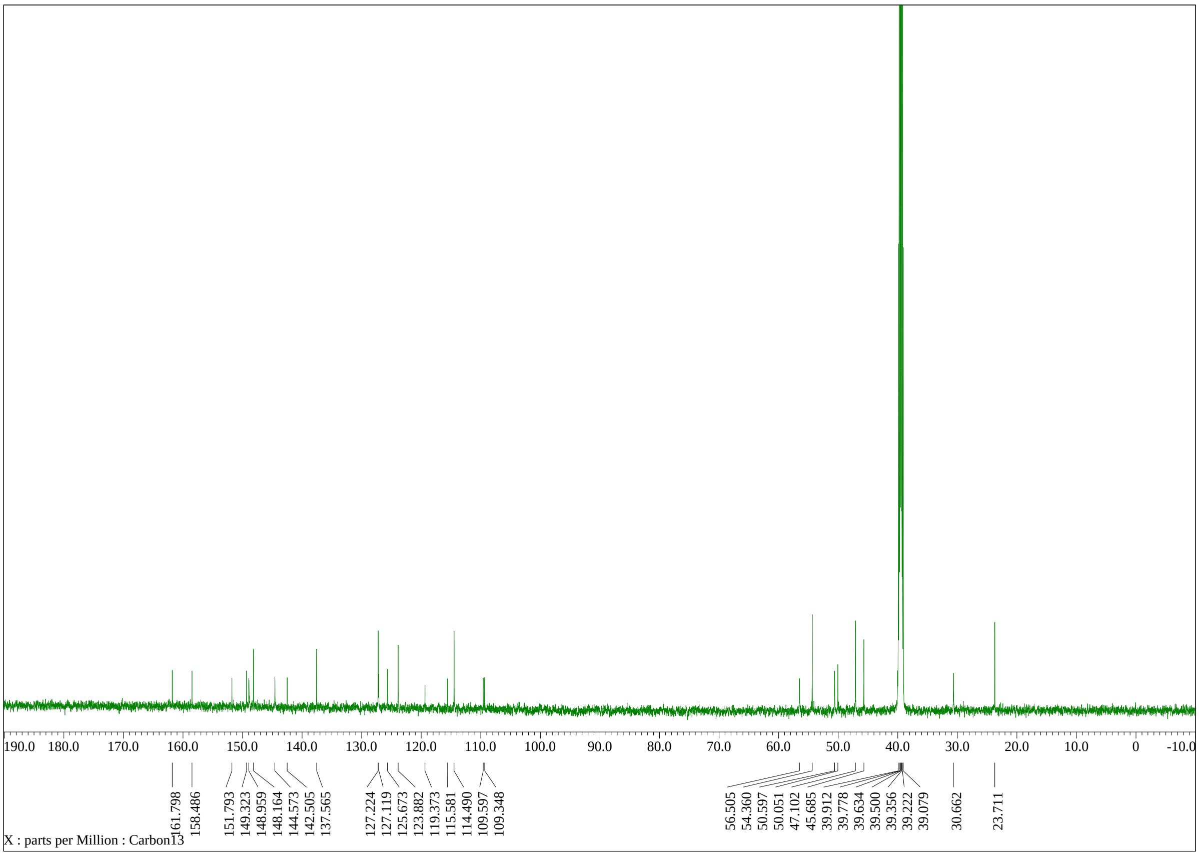
190.0 180.0 170.0 160.0 150.0 140.0 130.0 120.0 110.0 100.0 90.0 80.0 70.0 60.0 50.0 40.0 30.0 20.0 10.0 0 -10.0
161.798 158.486 151.793 149.323 148.959 148.164 144.573 142.505 137.565 127.224 127.119 125.673 123.882 119.373 115.581 114.490 109.597 109.348
56.505 54.360 50.597 50.051 47.102 45.685 39.912 39.778 39.634 39.500 39.356 39.222 39.079 30.662 23.711
X : parts per Million : Carbon13

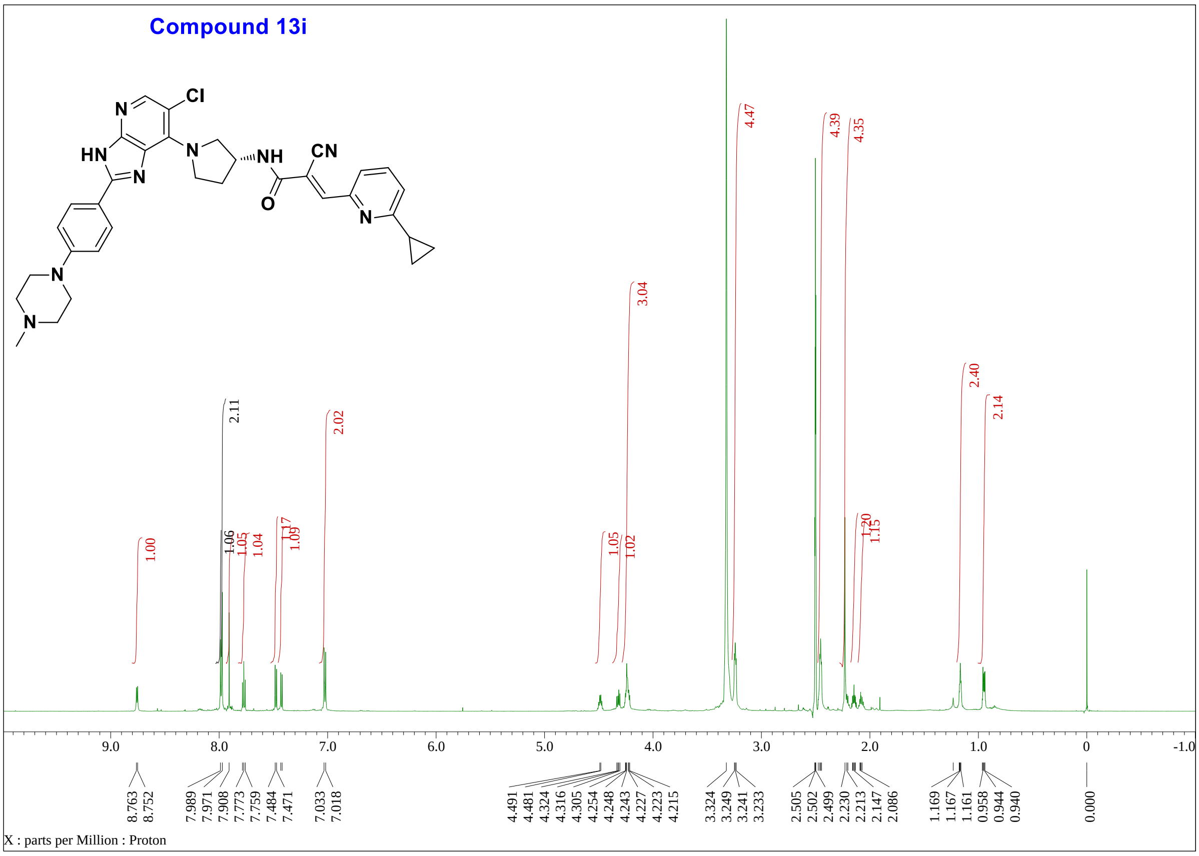
Compound 13i
N
Cl
HN
N
N
NH
CN
O
N
N
N
1.00 2.11 1.06 1.05 1.04 1.17 1.09 2.02 1.05 1.02 3.04 4.47 4.39 4.35 1.20 1.15 2.40 2.14
9.0 8.0 7.0 6.0 5.0 4.0 3.0 2.0 1.0 0 -1.0
8.763 8.752 7.989 7.971 7.908 7.773 7.759 7.484 7.471 7.033 7.018
4.491 4.481 4.324 4.316 4.305 4.254 4.248 4.243 4.227 4.223 4.215
3.324 3.249 3.241 3.233 2.505 2.502 2.499 2.230 2.213 2.147 2.086
1.169 1.167 1.161 0.958 0.944 0.940 0.000
X : parts per Million : Proton

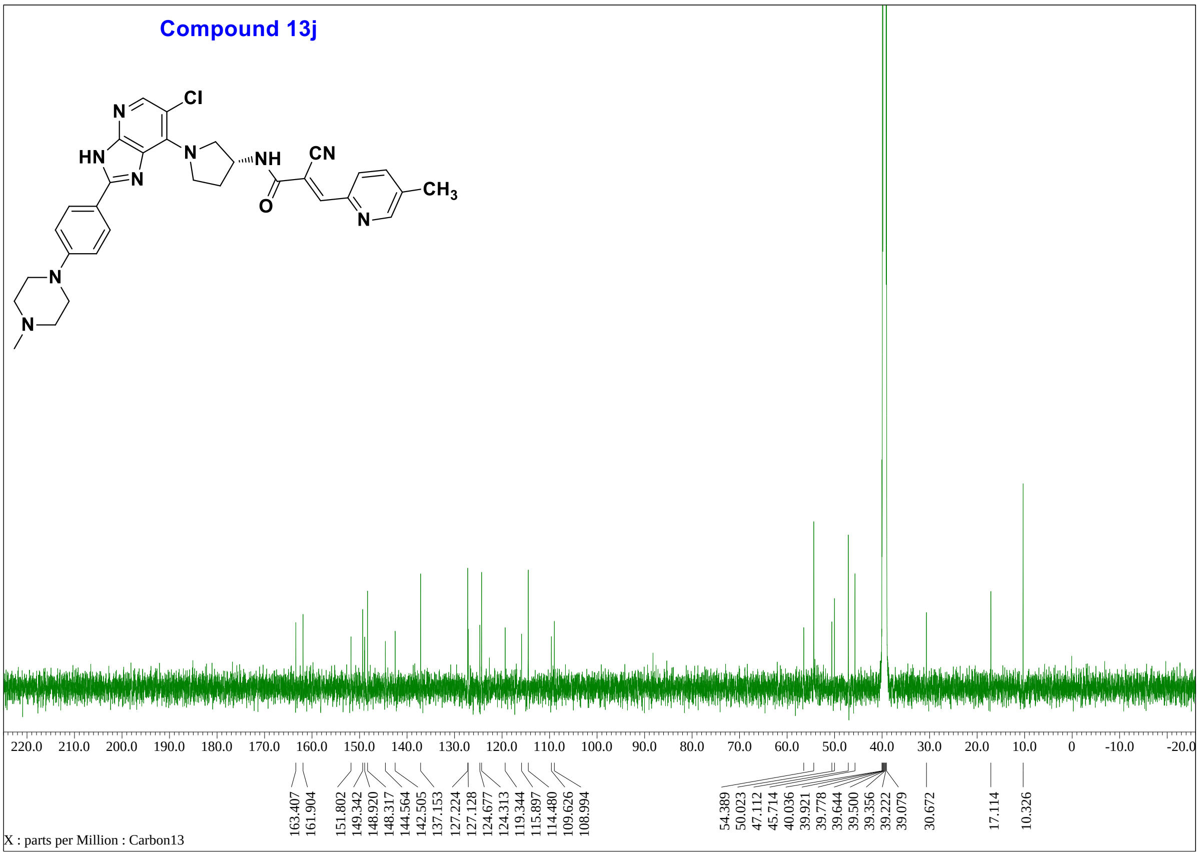
Compound 13j
Cl
N
HN
N
N
NH
CN
O
N
CH3
N
N
220.0 210.0 200.0 190.0 180.0 170.0 160.0 150.0 140.0 130.0 120.0 110.0 100.0 90.0 80.0 70.0 60.0 50.0 40.0 30.0 20.0 10.0 0 -10.0 -20.0
163.407
161.904
151.802
149.342
148.920
148.317
144.564
142.505
137.153
127.224
127.128
124.677
124.313
119.344
115.897
114.480
109.626
108.994
54.389
50.023
47.112
45.714
40.036
39.921
39.778
39.644
39.500
39.356
39.222
39.079
30.672
17.114
10.326
X : parts per Million : Carbon13

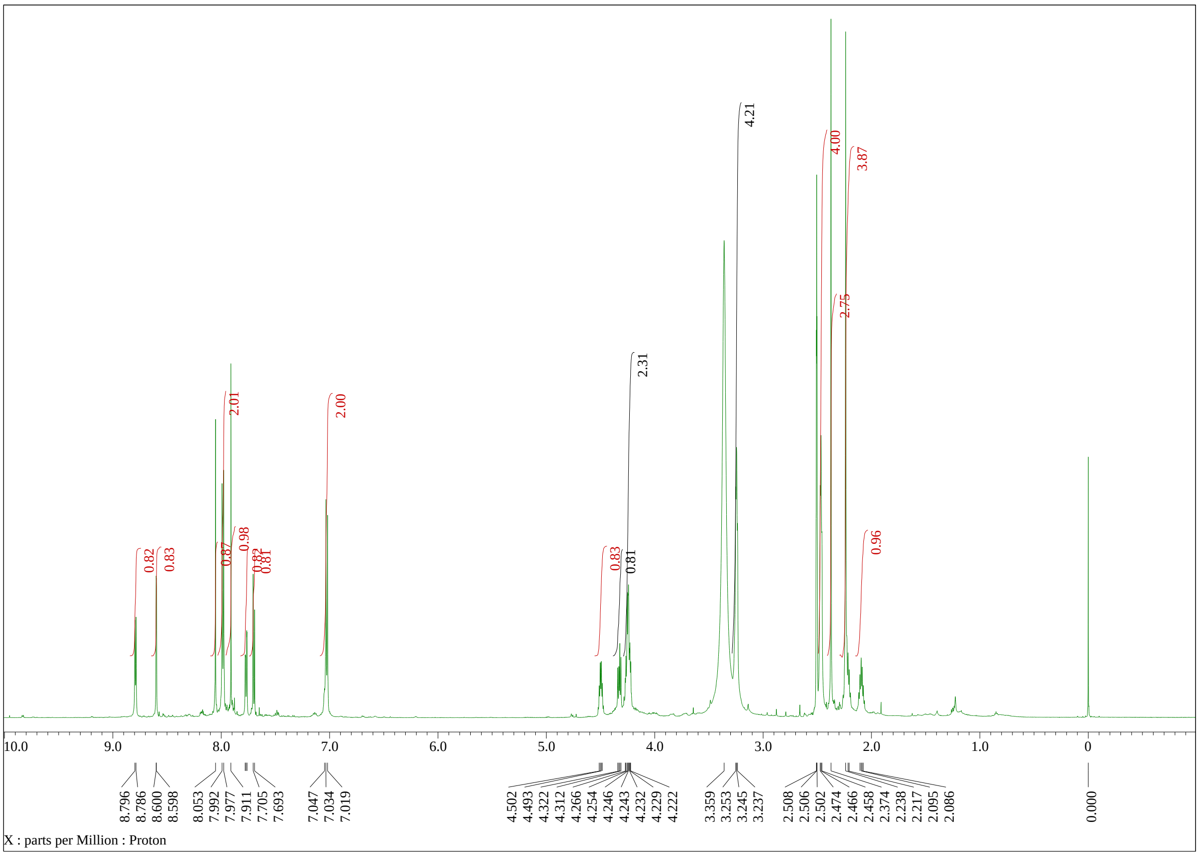
0.82
0.83
0.87
2.01
0.98
0.82
0.81
2.00
0.83
0.81
2.31
4.21
4.00
3.87
2.75
0.96
10.0 9.0 8.0 7.0 6.0 5.0 4.0 3.0 2.0 1.0 0
8.796
8.786
8.600
8.598
8.053
7.992
7.977
7.911
7.705
7.693
7.047
7.034
7.019
4.502
4.493
4.322
4.312
4.266
4.254
4.246
4.243
4.232
4.229
4.222
3.359
3.253
3.245
3.237
2.508
2.506
2.502
2.474
2.466
2.458
2.374
2.238
2.217
2.095
2.086
0.000
X : parts per Million : Proton

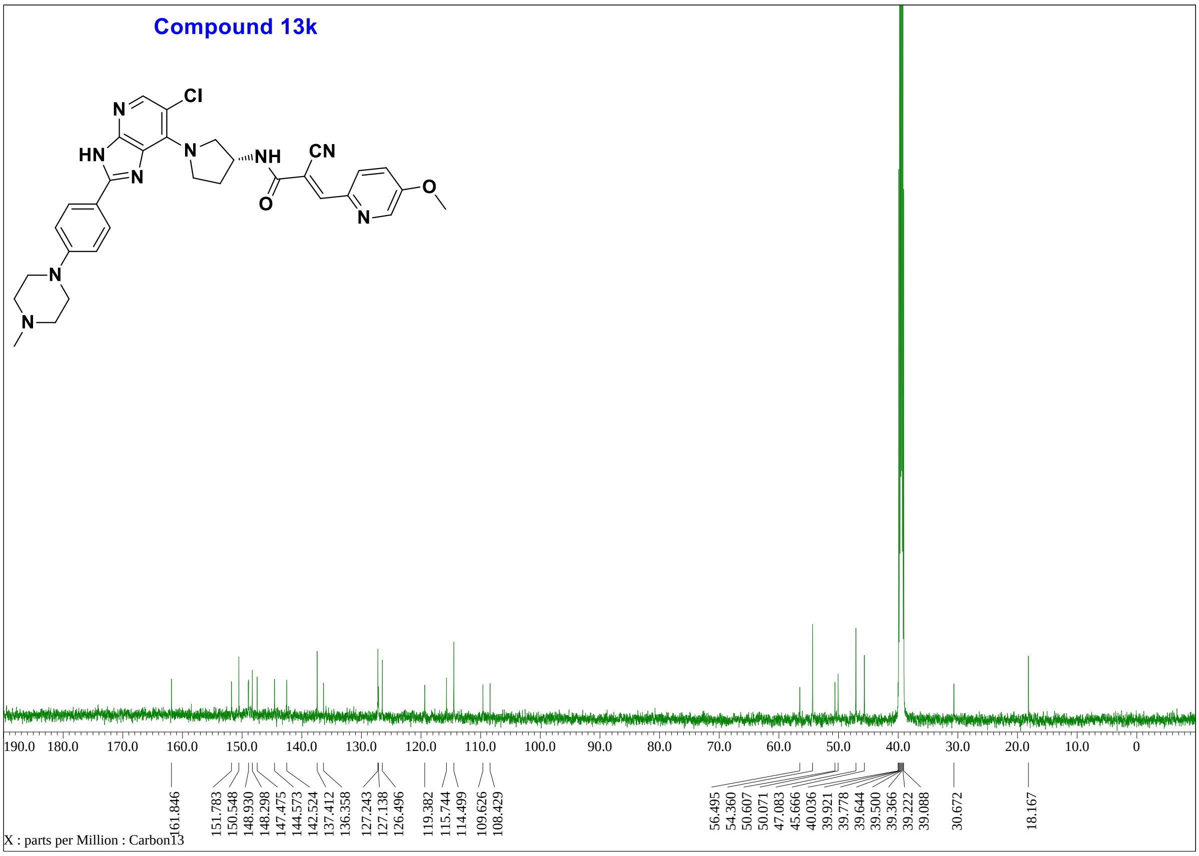
Compound 13k
Cl
N
HN
N
N
NH
CN
O
N
O
N
N
190.0 180.0 170.0 160.0 150.0 140.0 130.0 120.0 110.0 100.0 90.0 80.0 70.0 60.0 50.0 40.0 30.0 20.0 10.0 0
161.846
151.783 150.548 148.930 148.298 147.475 144.573 142.524 137.412 136.358
127.243 127.138 126.496
119.382 115.744 114.499 109.626 108.429
56.495 54.360 50.607 50.071 47.083 45.666 40.036 39.921 39.778 39.644 39.500 39.366 39.222 39.088
30.672
18.167
X : parts per Million : Carbon13

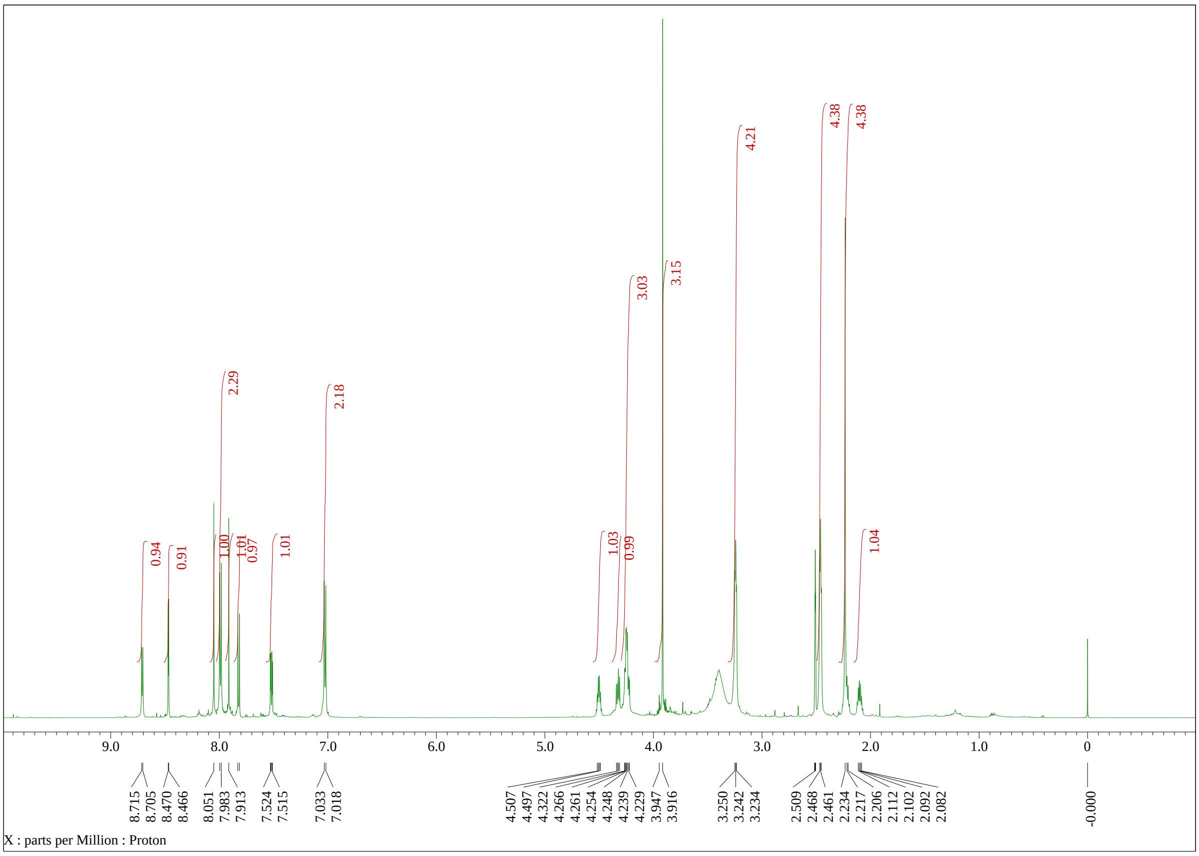
0.94 0.91 1.00 2.29 1.01 0.97 1.01 2.18 1.03 0.99 3.03 3.15 4.21 4.38 4.38 1.04
9.0 8.0 7.0 6.0 5.0 4.0 3.0 2.0 1.0 0
8.715 8.705 8.470 8.466 8.051 7.983 7.913 7.524 7.515 7.033 7.018
4.507 4.497 4.322 4.266 4.261 4.254 4.248 4.239 4.229 3.947 3.916
3.250 3.242 3.234
2.509 2.468 2.461 2.234 2.217 2.206 2.112 2.102 2.092 2.082
-0.000
X : parts per Million : Proton

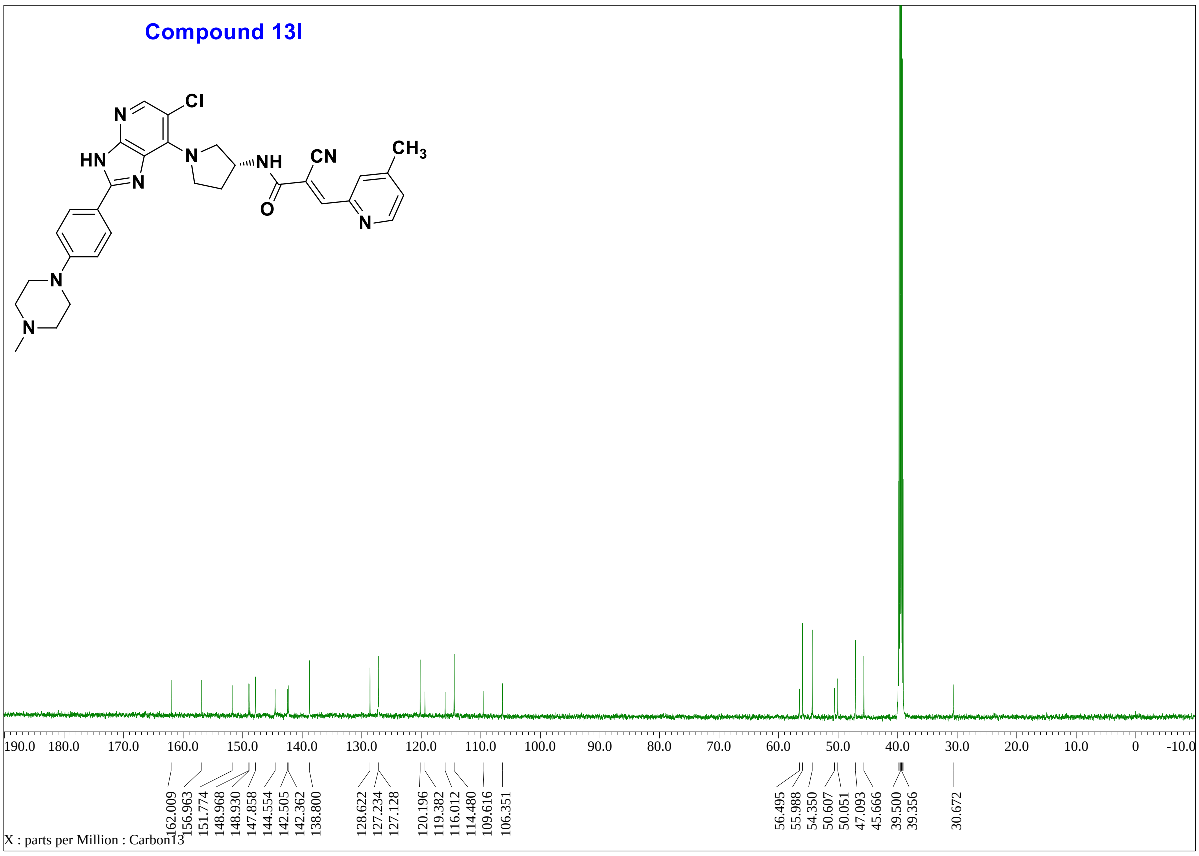
Compound 13l
Cl
N
HN
N
N
NH
CN
CH3
O
N
N
N
162.009
156.963
151.774
148.968
148.930
147.858
144.554
142.505
142.362
138.800
128.622
127.234
127.128
120.196
119.382
116.012
114.480
109.616
106.351
56.495
55.988
54.350
50.607
50.051
47.093
45.666
39.500
39.356
30.672
X : parts per Million : Carbon13

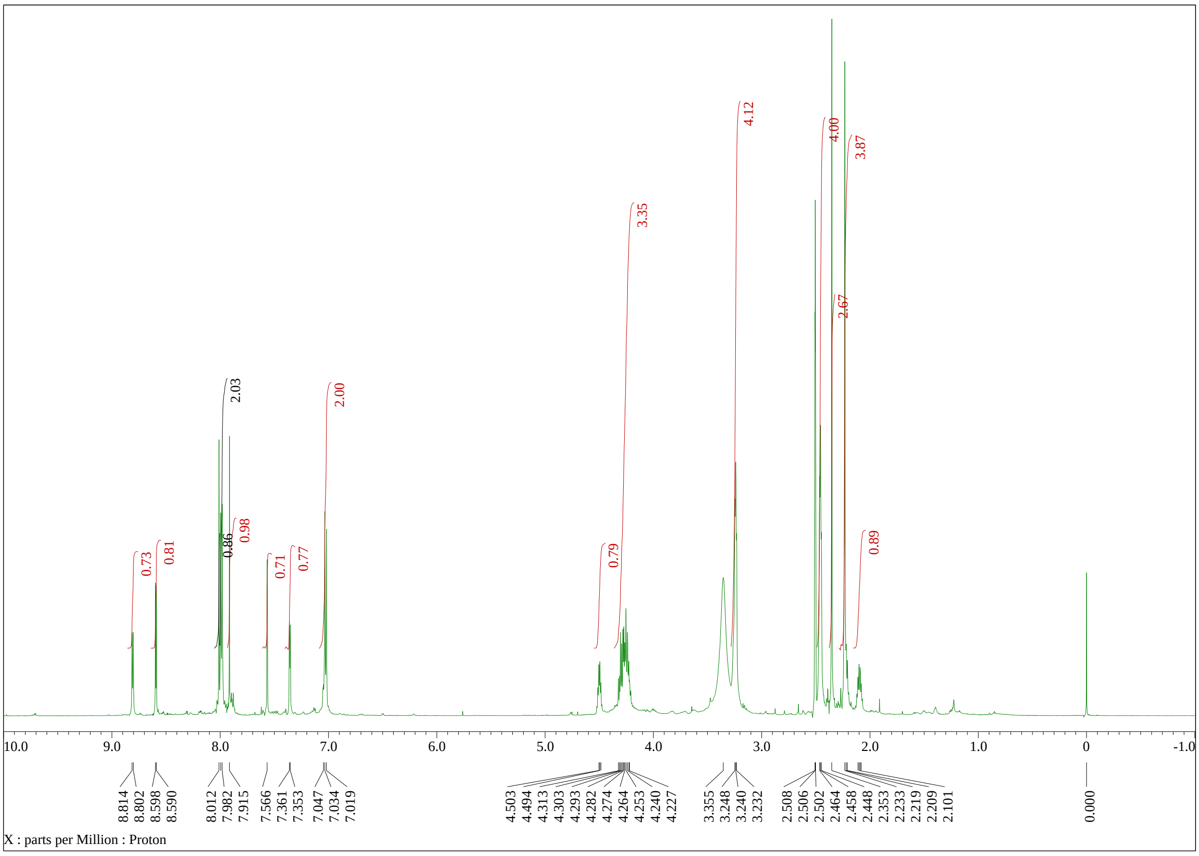
4.12
4.00
3.87
3.35
2.67
2.03
2.00
0.73
0.81
0.86
0.98
0.71
0.77
0.79
0.89
8.814
8.802
8.598
8.590
8.012
7.982
7.915
7.566
7.361
7.353
7.047
7.034
7.019
4.503
4.494
4.313
4.303
4.293
4.282
4.274
4.264
4.253
4.240
4.227
3.355
3.248
3.240
3.232
2.508
2.506
2.502
2.464
2.458
2.448
2.353
2.233
2.219
2.209
2.101
0.000
X : parts per Million : Proton

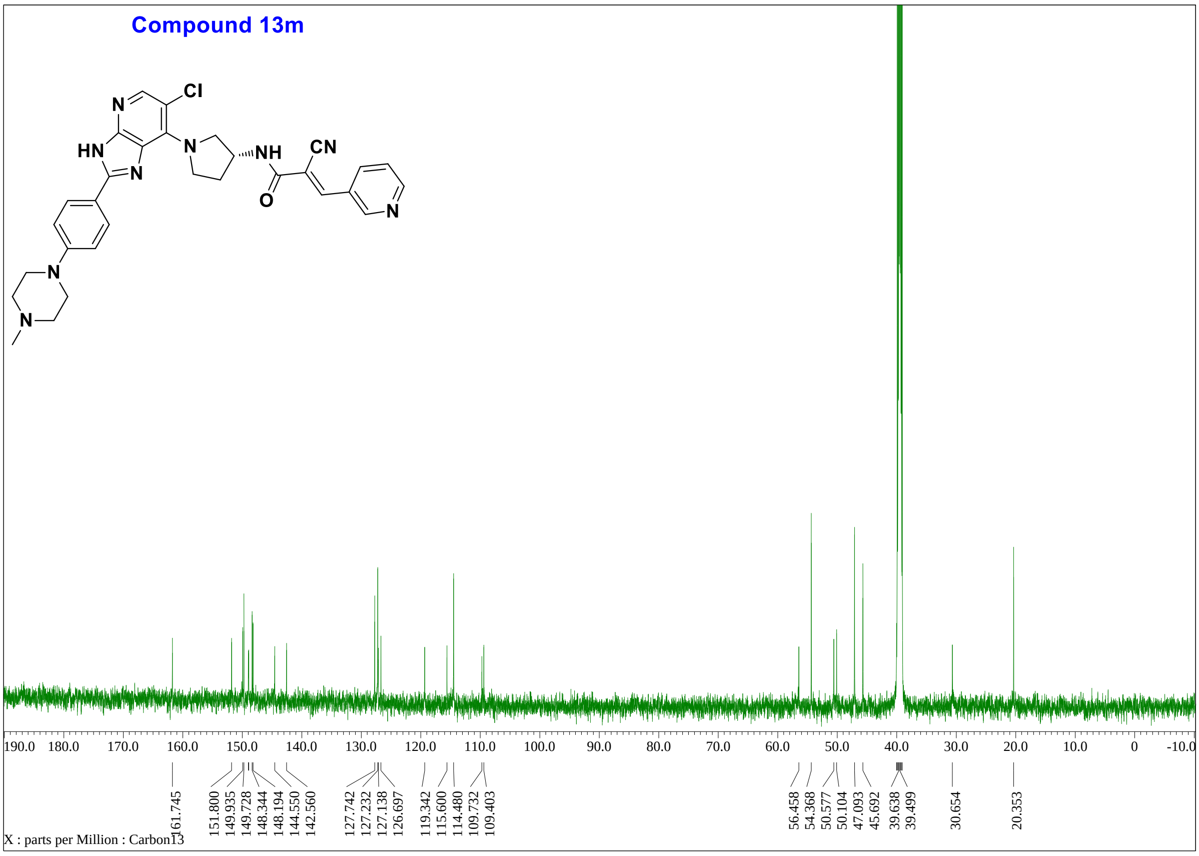
Compound 13m
Cl
N
HN
N
N
NH
CN
O
N
N
N
190.0 180.0 170.0 160.0 150.0 140.0 130.0 120.0 110.0 100.0 90.0 80.0 70.0 60.0 50.0 40.0 30.0 20.0 10.0 0 -10.0
161.745
151.800
149.935
149.728
148.344
148.194
144.550
142.560
127.742
127.232
127.138
126.697
119.342
115.600
114.480
109.732
109.403
56.458
54.368
50.577
50.104
47.093
45.692
39.638
39.499
30.654
20.353
X : parts per Million : Carbon13

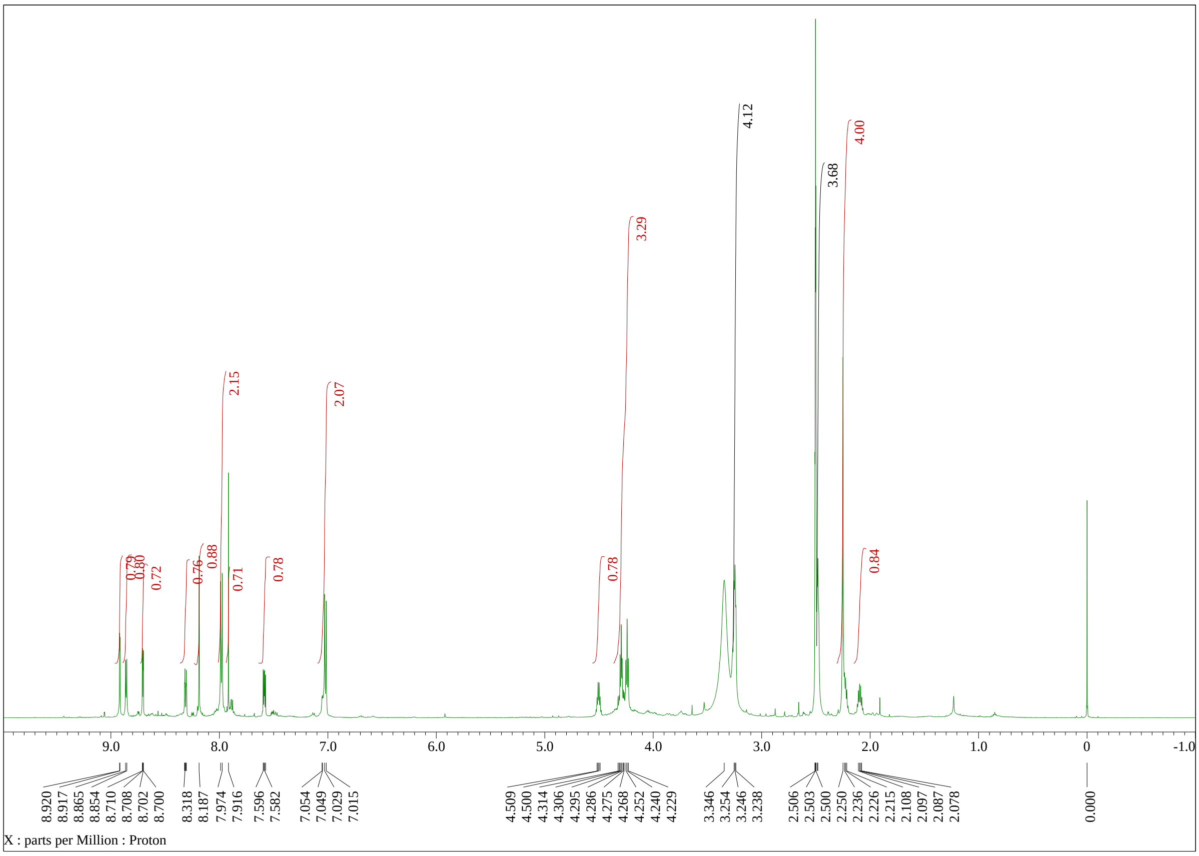
0.79
0.80
0.72
0.76
0.88
2.15
0.71
0.78
2.07
0.78
3.29
4.12
3.68
4.00
0.84
9.0 8.0 7.0 6.0 5.0 4.0 3.0 2.0 1.0 0 -1.0
8.920
8.917
8.865
8.854
8.710
8.708
8.702
8.700
8.318
8.187
7.974
7.916
7.596
7.582
7.054
7.049
7.029
7.015
4.509
4.500
4.314
4.306
4.295
4.286
4.275
4.268
4.252
4.240
4.229
3.346
3.254
3.246
3.238
2.506
2.503
2.500
2.250
2.236
2.226
2.215
2.108
2.097
2.087
2.078
0.000
X : parts per Million : Proton

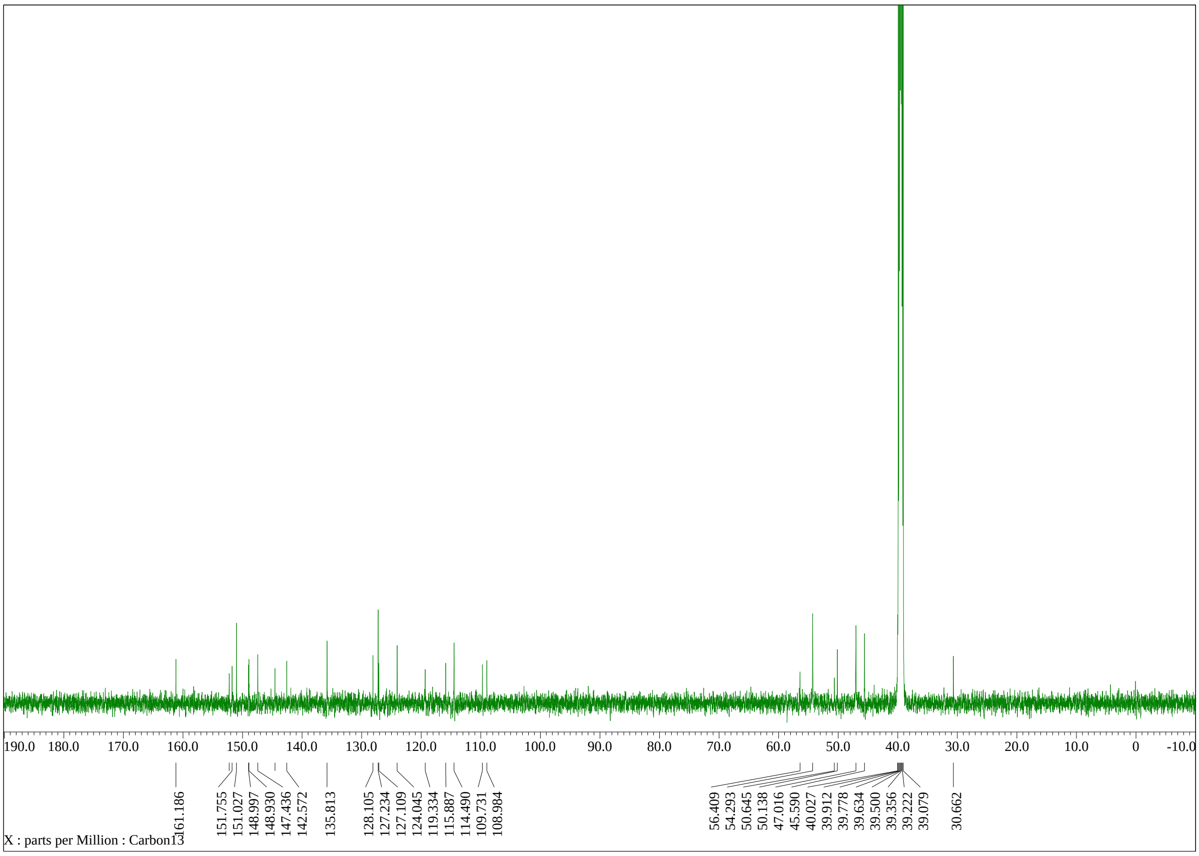
190.0 180.0 170.0 160.0 150.0 140.0 130.0 120.0 110.0 100.0 90.0 80.0 70.0 60.0 50.0 40.0 30.0 20.0 10.0 0 -10.0
161.186 151.755 151.027 148.997 148.930 147.436 142.572 135.813 128.105 127.234 127.109 124.045 119.334 115.887 114.490 109.731 108.984
56.409 54.293 50.645 50.138 47.016 45.590 40.027 39.912 39.778 39.634 39.500 39.356 39.222 39.079 30.662
X : parts per Million : Carbon13

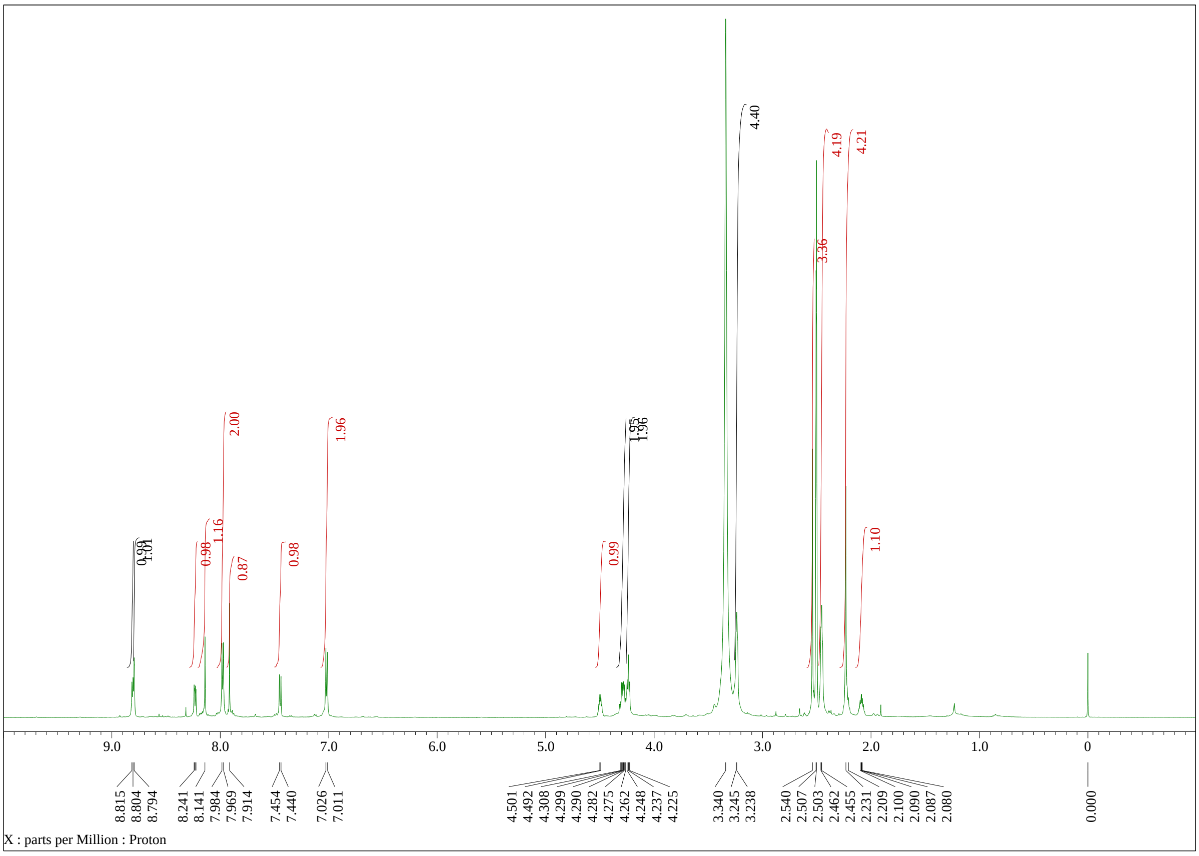
9.0 8.0 7.0 6.0 5.0 4.0 3.0 2.0 1.0 0
8.815 8.804 8.794 8.241 8.141 7.984 7.969 7.914 7.454 7.440 7.026 7.011
4.501 4.492 4.308 4.299 4.290 4.282 4.275 4.262 4.248 4.237 4.225
3.340 3.245 3.238 2.540 2.507 2.503 2.462 2.455 2.231 2.209 2.100 2.090 2.087 2.080 0.000
X : parts per Million : Proton

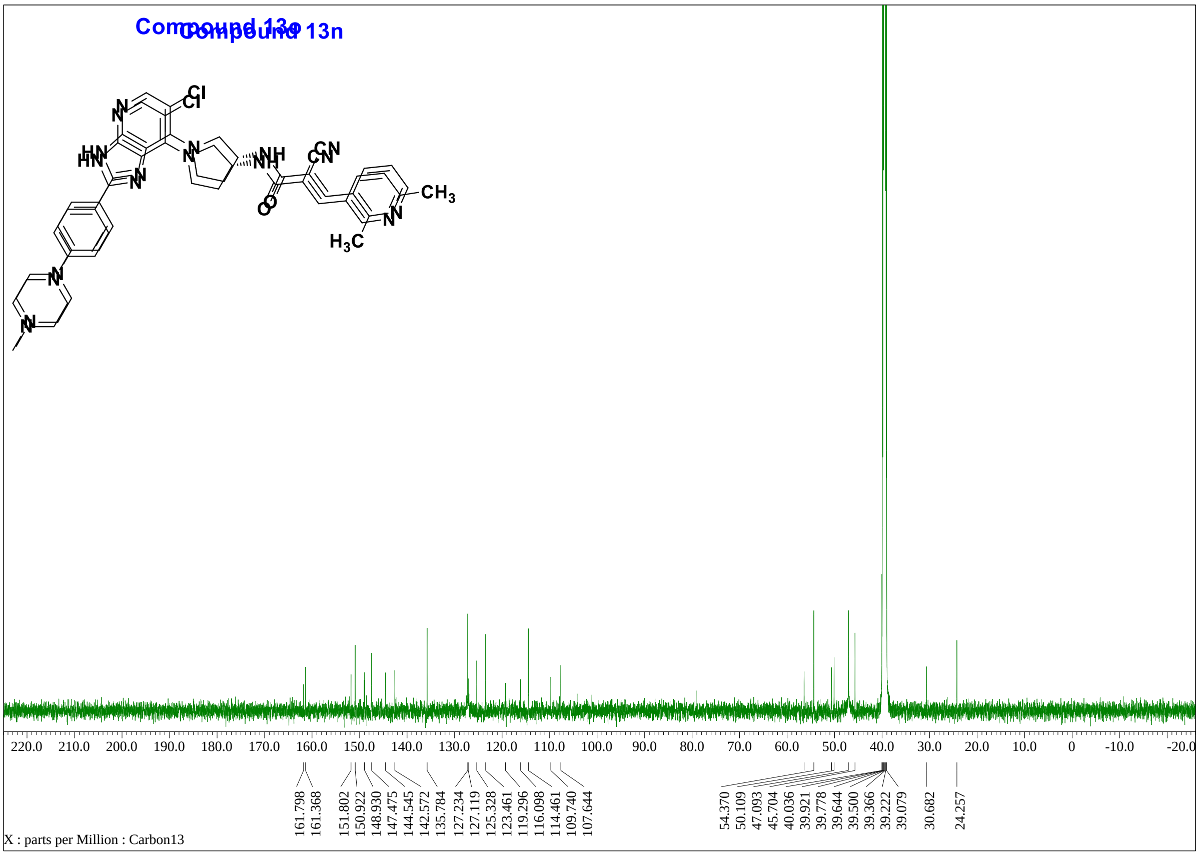
Compound 13n
CH3
H3C
CN
NH
HN
Cl
220.0 210.0 200.0 190.0 180.0 170.0 160.0 150.0 140.0 130.0 120.0 110.0 100.0 90.0 80.0 70.0 60.0 50.0 40.0 30.0 20.0 10.0 0 -10.0 -20.0
161.798 161.368 151.802 150.922 148.930 147.475 144.545 142.572 135.784 127.234 127.119 125.328 123.461 119.296 116.098 114.461 109.740 107.644
54.370 50.109 47.093 45.704 40.036 39.921 39.778 39.644 39.500 39.366 39.222 39.079 30.682 24.257
X : parts per Million : Carbon13

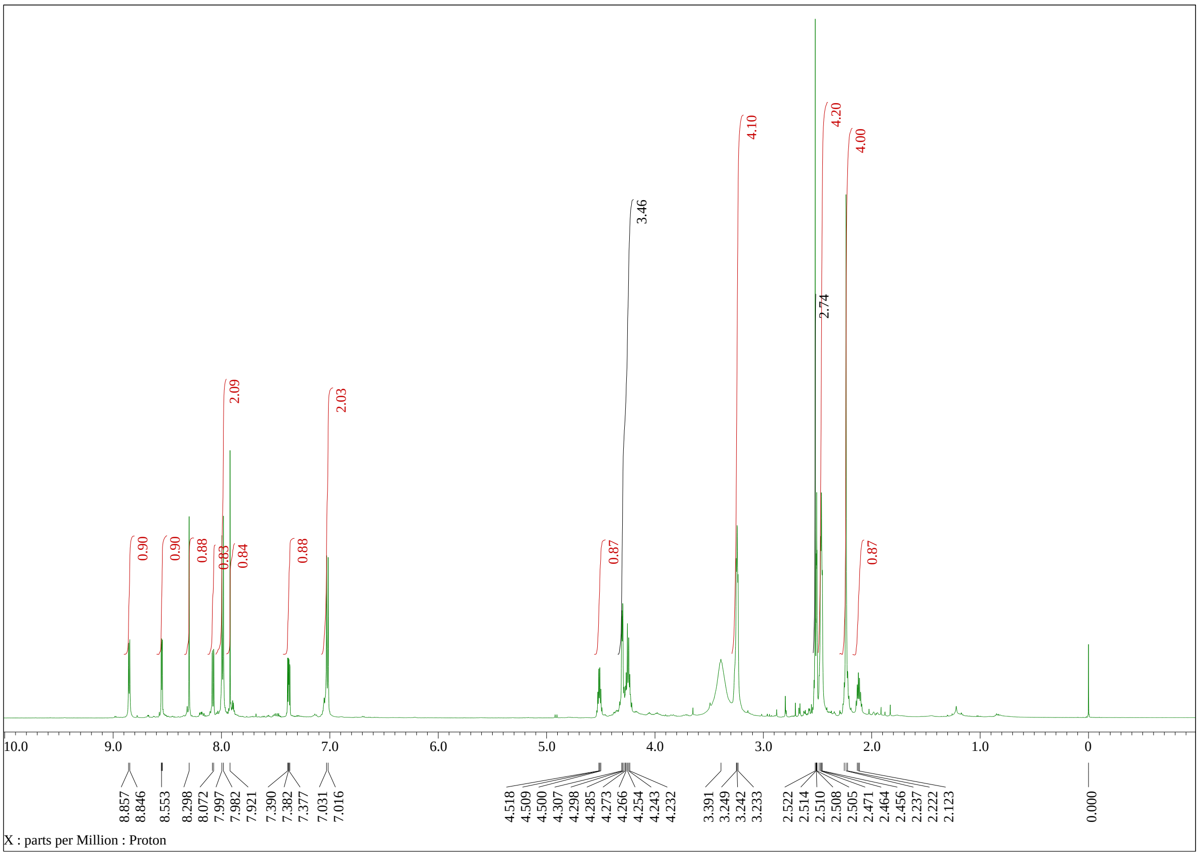
0.90 0.90 0.88 0.83 2.09 0.84 0.88 2.03 0.87 3.46 4.10 2.74 4.20 4.00 0.87
10.0 9.0 8.0 7.0 6.0 5.0 4.0 3.0 2.0 1.0 0
8.857 8.846 8.553 8.298 8.072 7.997 7.982 7.921 7.390 7.382 7.377 7.031 7.016
4.518 4.509 4.500 4.307 4.298 4.285 4.273 4.266 4.254 4.243 4.232 3.391 3.249 3.242 3.233 2.522 2.514 2.510 2.508 2.505 2.471 2.464 2.456 2.237 2.222 2.123 0.000
X : parts per Million : Proton

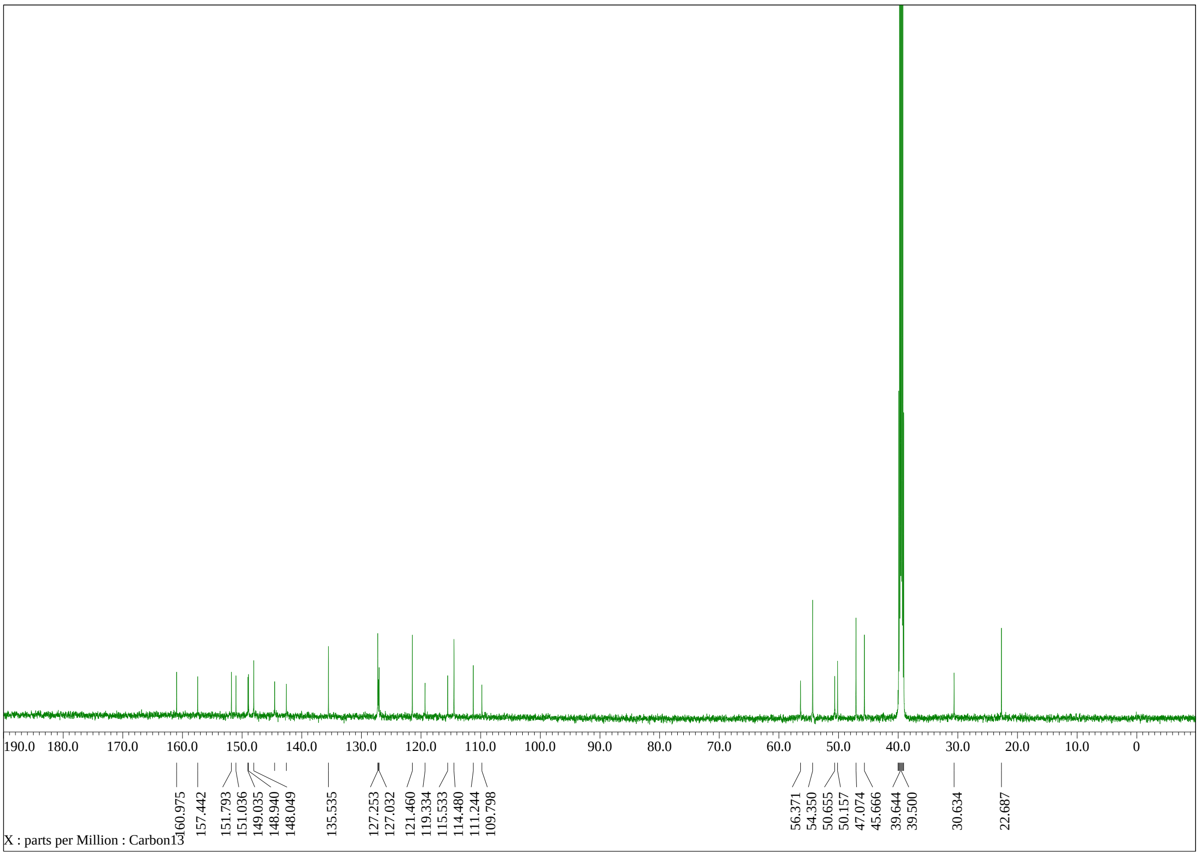
160.975
157.442
151.793
151.036
149.035
148.940
148.049
135.535
127.253
127.032
121.460
119.334
115.533
114.480
111.244
109.798
56.371
54.350
50.655
50.157
47.074
45.666
39.644
39.500
30.634
22.687
X : parts per Million : Carbon13

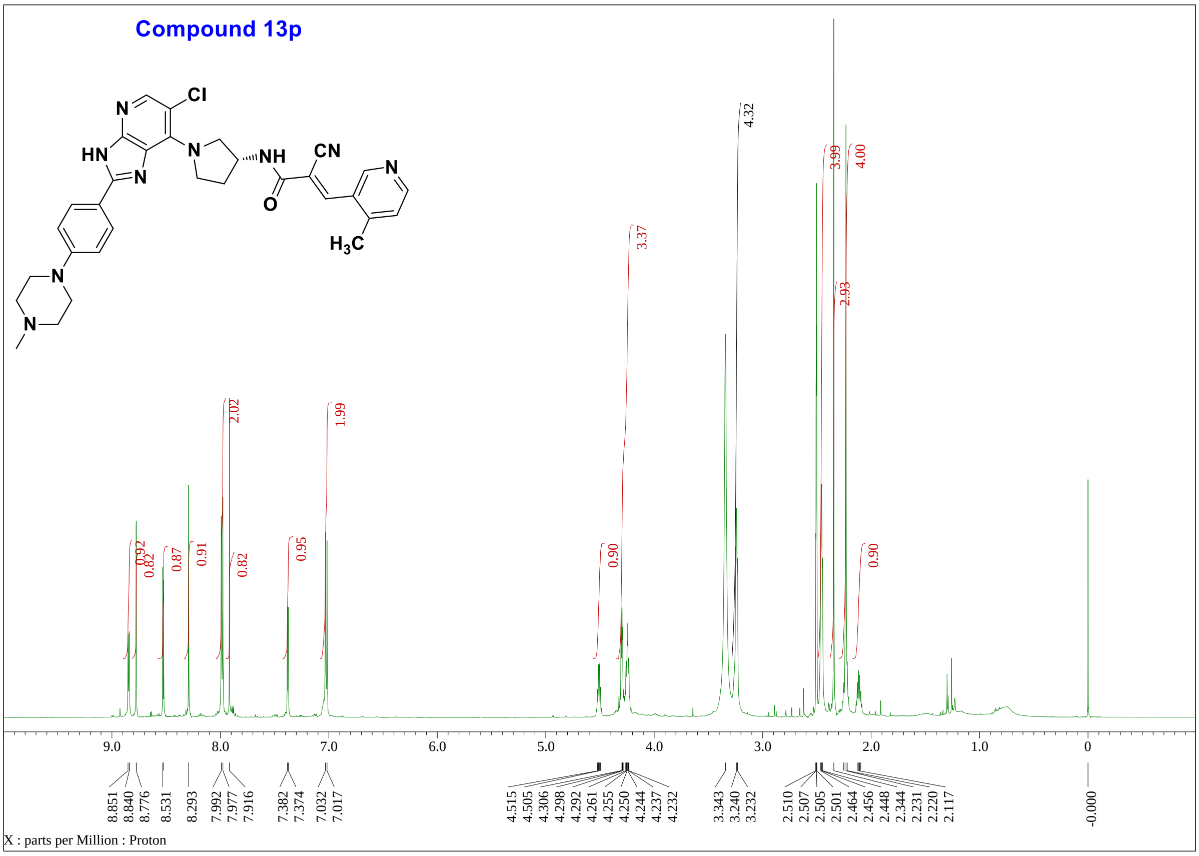
Compound 13p
Cl
N
HN
N
N
NH
CN
N
O
H_3C
N
N
4.32
3.99
4.00
3.37
2.93
2.02
1.99
0.92
0.82
0.87
0.91
0.82
0.95
0.90
0.90
8.851
8.840
8.776
8.531
8.293
7.992
7.977
7.916
7.382
7.374
7.032
7.017
4.515
4.505
4.306
4.298
4.292
4.261
4.255
4.250
4.244
4.237
4.232
3.343
3.240
3.232
2.510
2.507
2.505
2.501
2.464
2.456
2.448
2.344
2.231
2.220
2.117
-0.000
X : parts per Million : Proton

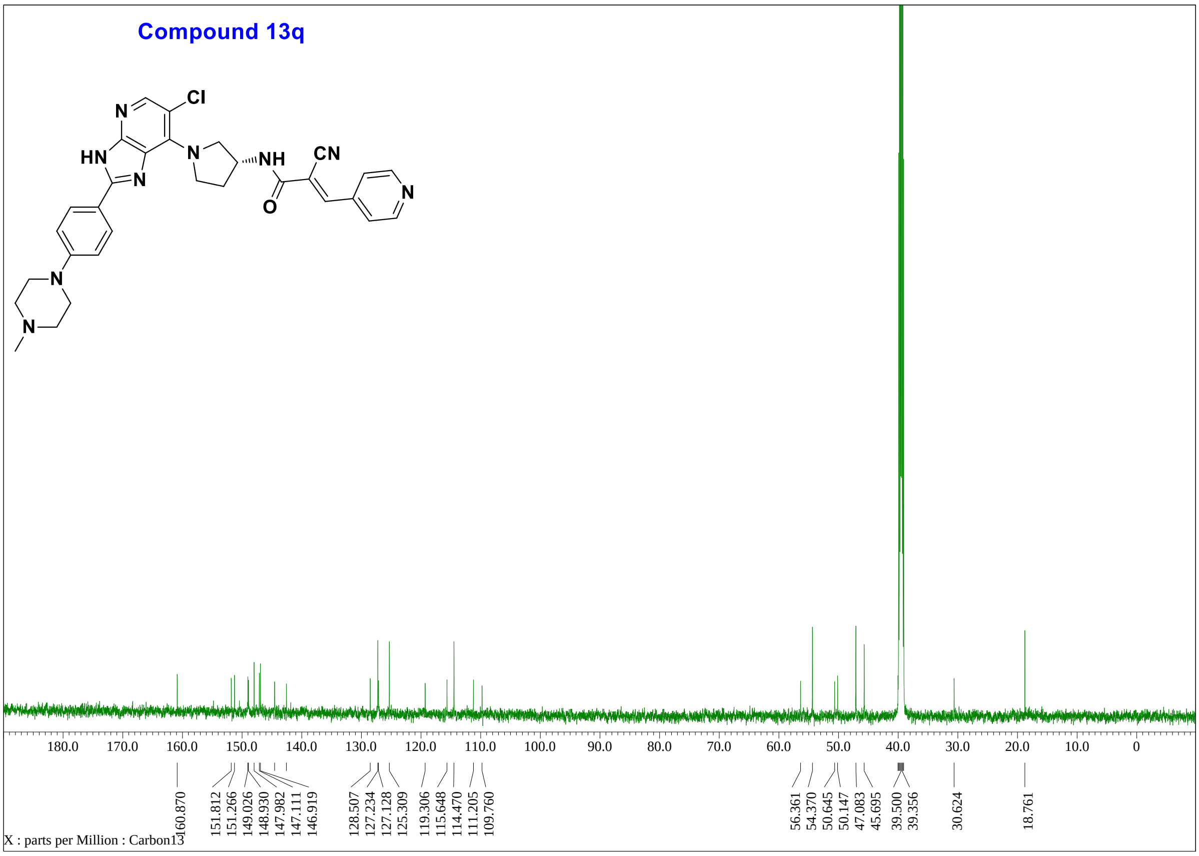
Compound 13q
Cl
N
HN
N
N
NH
CN
O
N
N
N
180.0 170.0 160.0 150.0 140.0 130.0 120.0 110.0 100.0 90.0 80.0 70.0 60.0 50.0 40.0 30.0 20.0 10.0 0
160.870
151.812
151.266
149.026
148.930
147.982
147.111
146.919
128.507
127.234
127.128
125.309
119.306
115.648
114.470
111.205
109.760
56.361
54.370
50.645
50.147
47.083
45.695
39.500
39.356
30.624
18.761
X : parts per Million : Carbon13

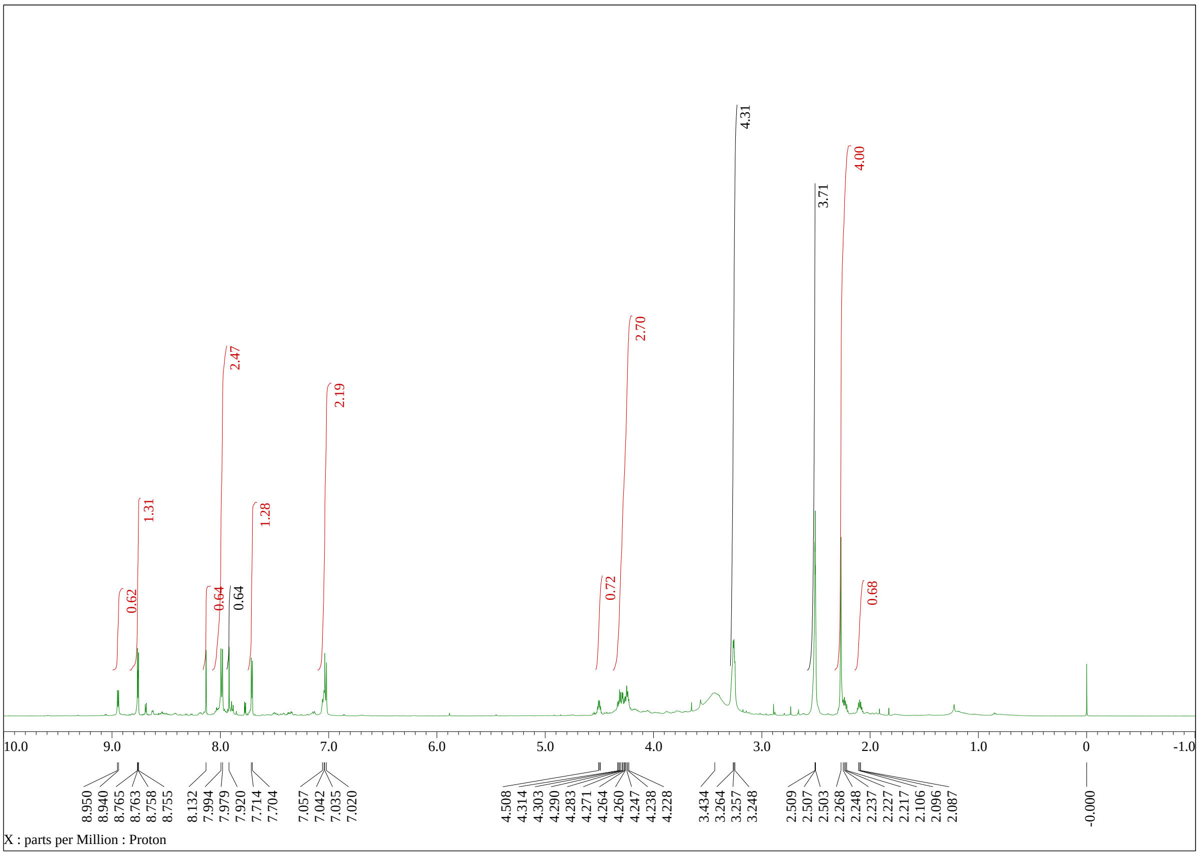
0.62
1.31
0.64
2.47
0.64
1.28
2.19
0.72
2.70
4.31
3.71
4.00
0.68
10.0 9.0 8.0 7.0 6.0 5.0 4.0 3.0 2.0 1.0 0 -1.0
8.950
8.940
8.765
8.763
8.758
8.755
8.132
7.994
7.979
7.920
7.714
7.704
7.057
7.042
7.035
7.020
4.508
4.314
4.303
4.290
4.283
4.271
4.264
4.260
4.247
4.238
4.228
3.434
3.264
3.257
3.248
2.509
2.507
2.503
2.268
2.248
2.237
2.227
2.217
2.106
2.096
2.087
-0.000
X : parts per Million : Proton

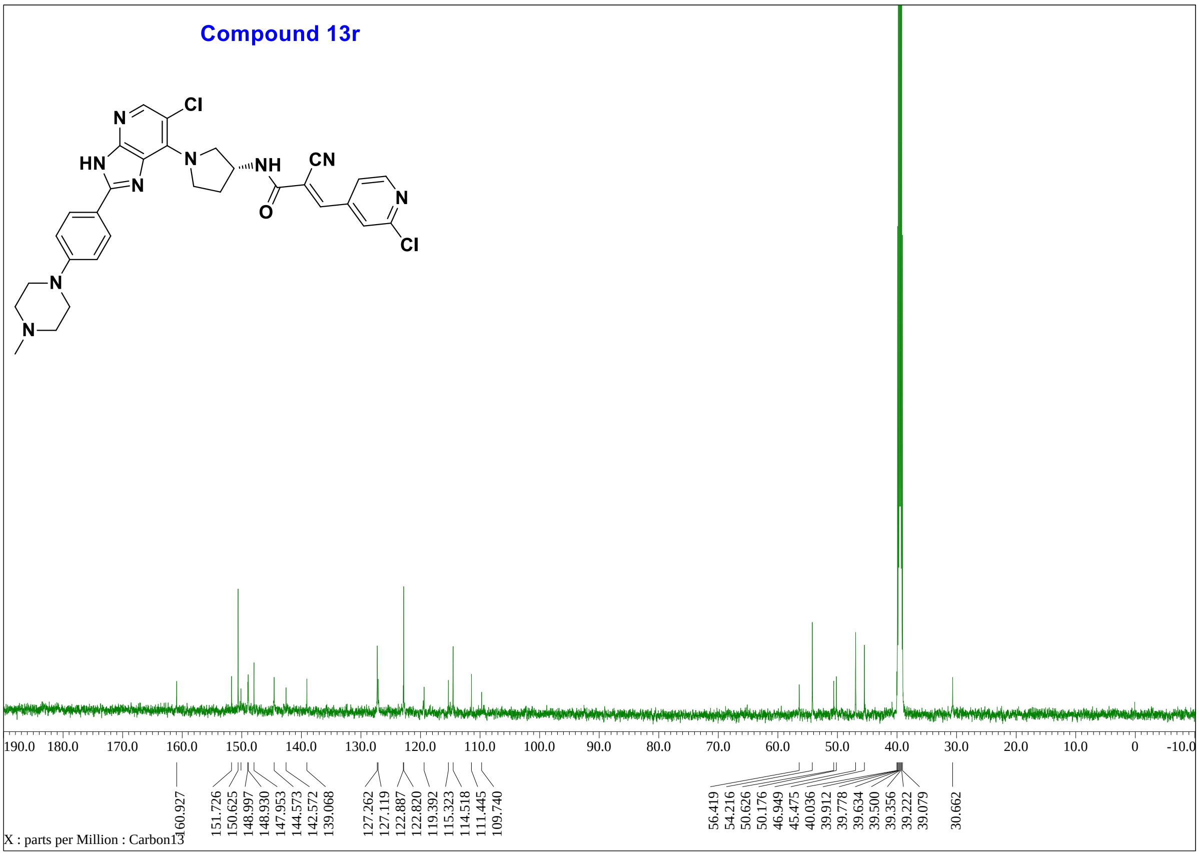
Compound 13r
Cl
N
HN
N
N
NH
CN
O
N
Cl
N
N
190.0 180.0 170.0 160.0 150.0 140.0 130.0 120.0 110.0 100.0 90.0 80.0 70.0 60.0 50.0 40.0 30.0 20.0 10.0 0 -10.0
160.927
151.726 150.625 148.997 148.930 147.953 144.573 142.572 139.068
127.262 127.119 122.887 122.820 119.392 115.323 114.518 111.445 109.740
56.419 54.216 50.626 50.176 46.949 45.475 40.036 39.912 39.778 39.634 39.500 39.356 39.222 39.079
30.662
X : parts per Million : Carbon13

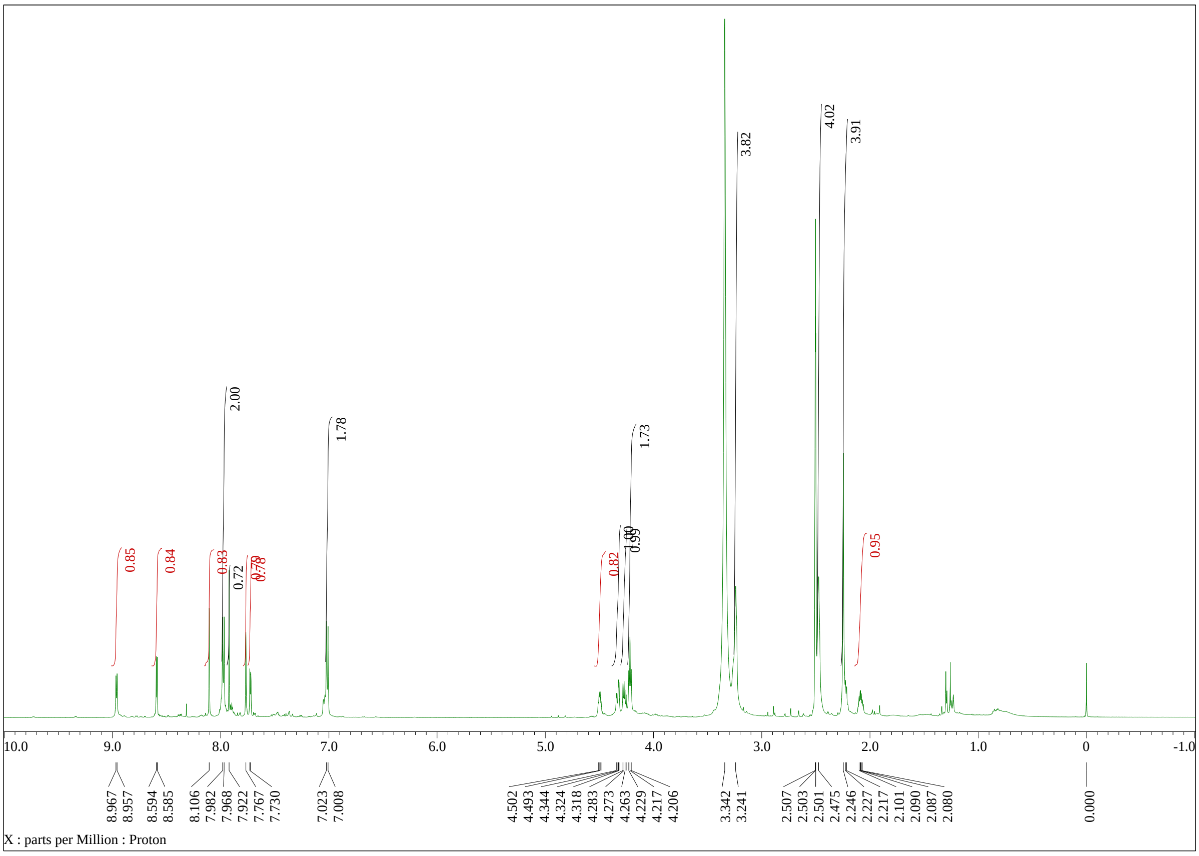
0.85 0.84 0.83 0.72 2.00 0.79 0.78 1.78 0.82 1.00 0.99 1.73 3.82 4.02 3.91 0.95
10.0 9.0 8.0 7.0 6.0 5.0 4.0 3.0 2.0 1.0 0 -1.0
8.967 8.957 8.594 8.585 8.106 7.982 7.968 7.922 7.767 7.730 7.023 7.008
4.502 4.493 4.344 4.324 4.318 4.283 4.273 4.263 4.229 4.217 4.206
3.342 3.241
2.507 2.503 2.501 2.475 2.246 2.227 2.217 2.101 2.090 2.087 2.080
0.000
X : parts per Million : Proton

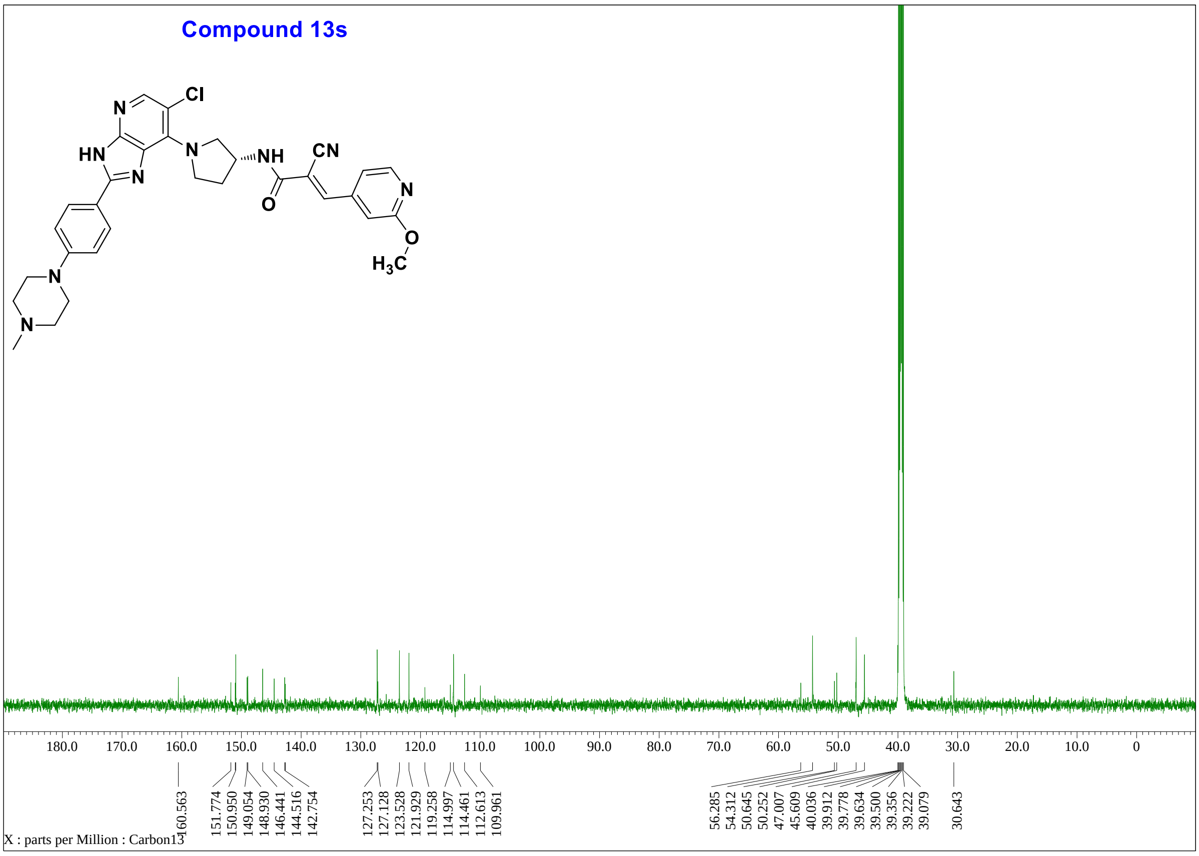
Compound 13s
Cl
N
HN
N
N
NH
CN
O
N
O
H3C
N
N
180.0 170.0 160.0 150.0 140.0 130.0 120.0 110.0 100.0 90.0 80.0 70.0 60.0 50.0 40.0 30.0 20.0 10.0 0
160.563
151.774 150.950 149.054 148.930 146.441 144.516 142.754
127.253 127.128 123.528 121.929 119.258 114.997 114.461 112.613 109.961
56.285 54.312 50.645 50.252 47.007 45.609 40.036 39.912 39.778 39.634 39.500 39.356 39.222 39.079
30.643
X : parts per Million : Carbon13

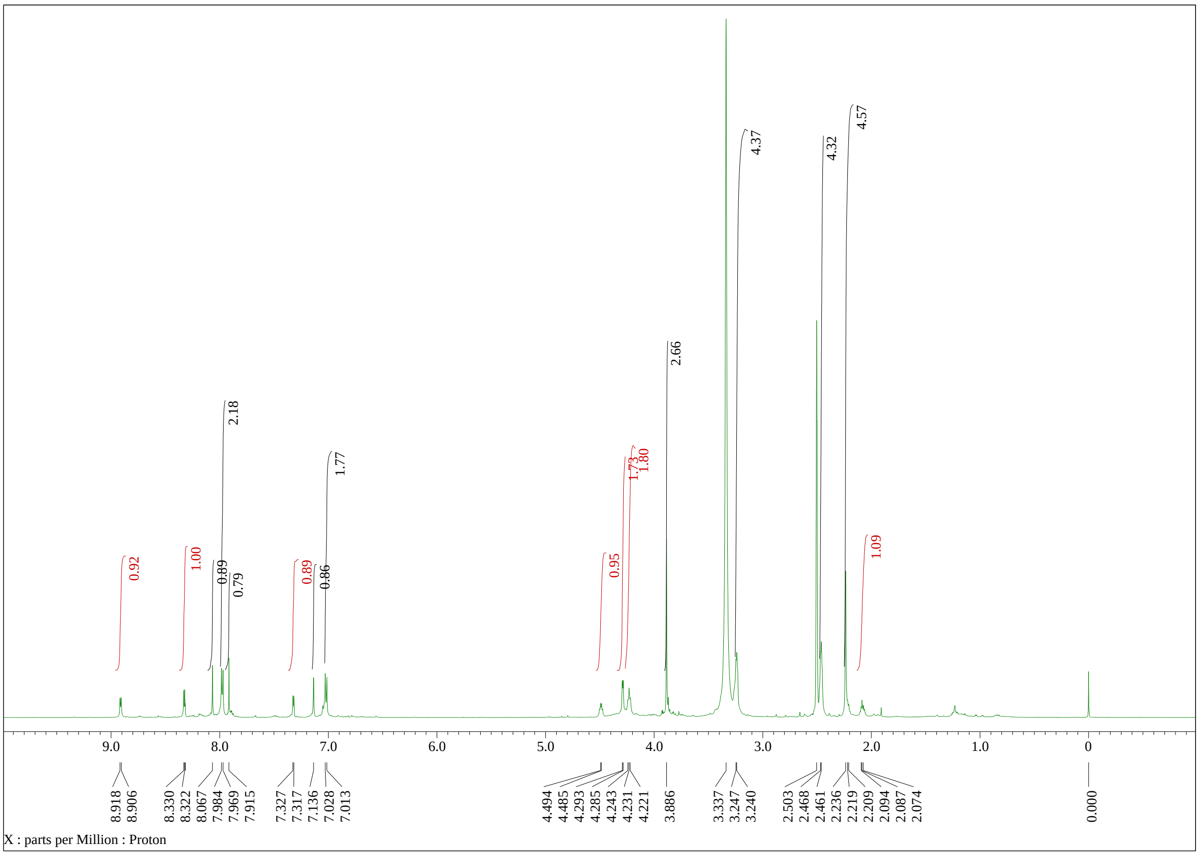
0.92 1.00 0.89 0.79 2.18 0.89 0.86 1.77 0.95 1.73 1.80 2.66 4.37 4.32 4.57 1.09
9.0 8.0 7.0 6.0 5.0 4.0 3.0 2.0 1.0 0
8.918 8.906 8.330 8.322 8.067 7.984 7.969 7.915 7.327 7.317 7.136 7.028 7.013
4.494 4.485 4.293 4.285 4.243 4.231 4.221 3.886 3.337 3.247 3.240
2.503 2.468 2.461 2.236 2.219 2.209 2.094 2.087 2.074
0.000
X : parts per Million : Proton

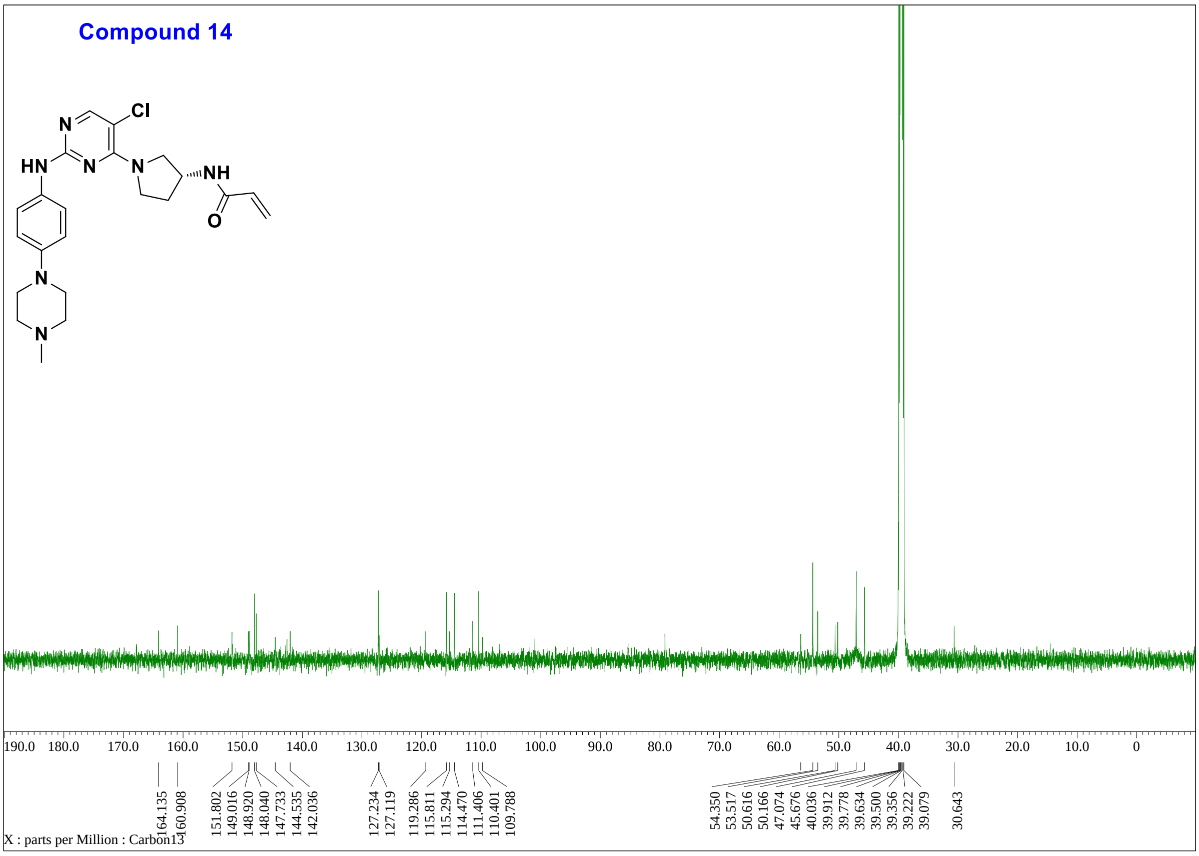
Compound 14
Cl
N
HN
N
N
NH
O
N
N
190.0 180.0 170.0 160.0 150.0 140.0 130.0 120.0 110.0 100.0 90.0 80.0 70.0 60.0 50.0 40.0 30.0 20.0 10.0 0
164.135
160.908
151.802
149.016
148.920
148.040
147.733
144.535
142.036
127.234
127.119
119.286
115.811
115.294
114.470
111.406
110.401
109.788
54.350
53.517
50.616
50.166
47.074
45.676
40.036
39.912
39.778
39.634
39.500
39.356
39.222
39.079
30.643
X : parts per Million : Carbon13

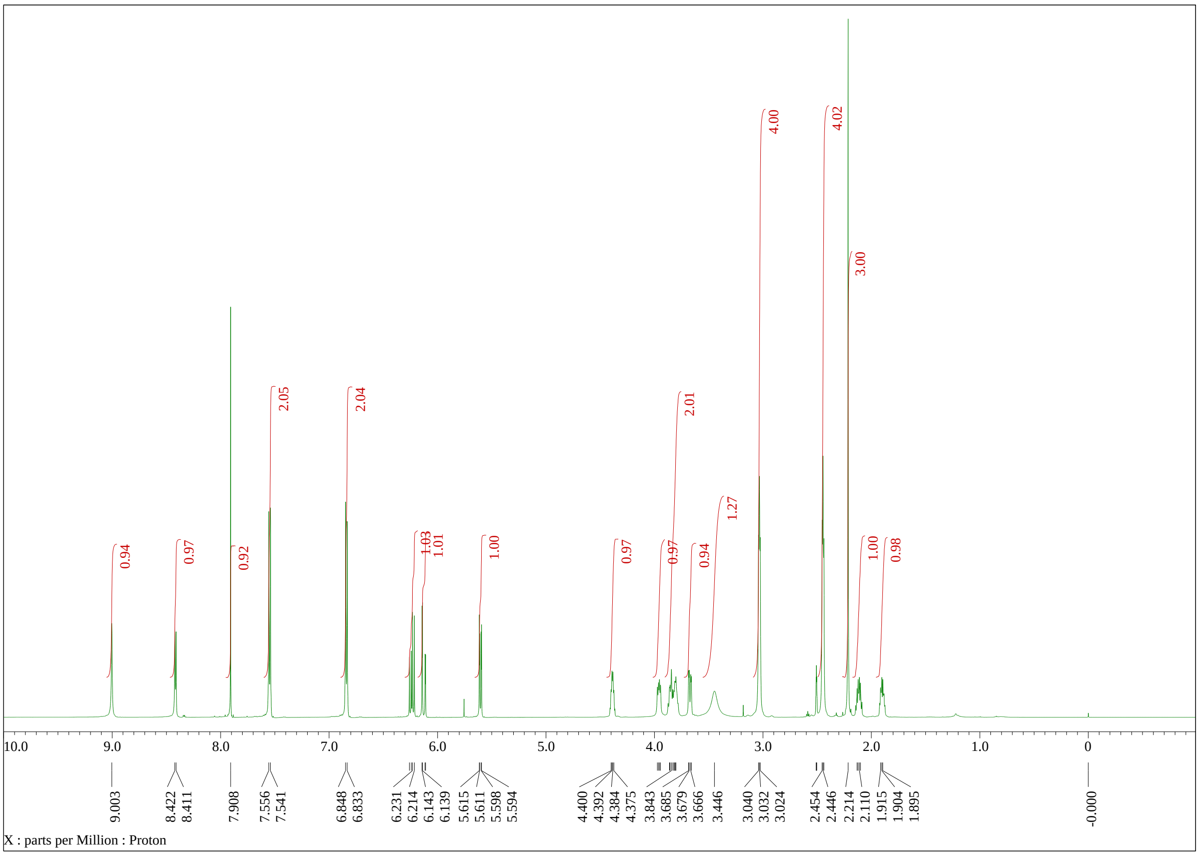
0.94
0.97
0.92
2.05
2.04
1.03
1.01
1.00
0.97
0.97
0.94
1.27
2.01
4.00
4.02
3.00
1.00
0.98
10.0 9.0 8.0 7.0 6.0 5.0 4.0 3.0 2.0 1.0 0
9.003
8.422
8.411
7.908
7.556
7.541
6.848
6.833
6.231
6.214
6.143
6.139
5.615
5.611
5.598
5.594
4.400
4.392
4.384
4.375
3.843
3.685
3.679
3.666
3.446
3.040
3.032
3.024
2.454
2.446
2.214
2.110
1.915
1.904
1.895
-0.000
X : parts per Million : Proton

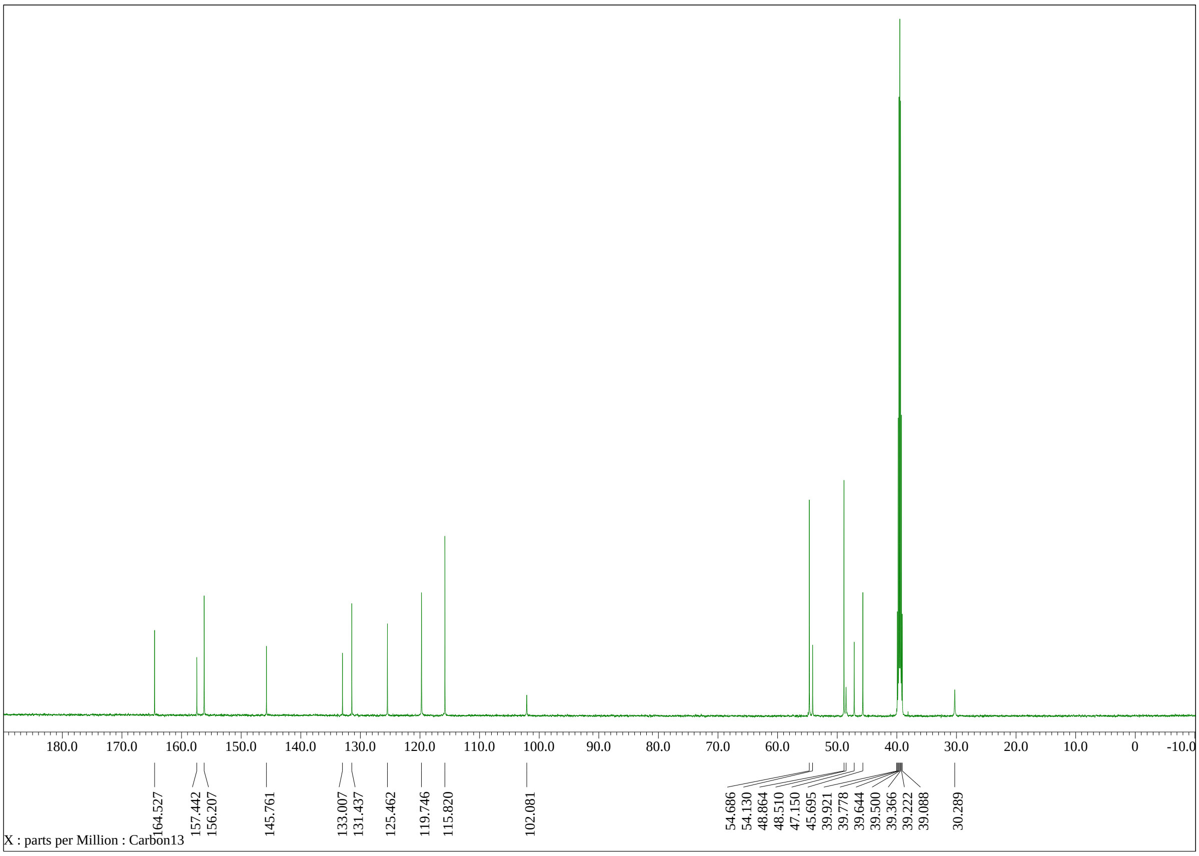
180.0
170.0
160.0
150.0
140.0
130.0
120.0
110.0
100.0
90.0
80.0
70.0
60.0
50.0
40.0
30.0
20.0
10.0
0
-10.0
164.527
157.442
156.207
145.761
133.007
131.437
125.462
119.746
115.820
102.081
54.686
54.130
48.864
48.510
47.150
45.695
39.921
39.778
39.644
39.500
39.366
39.222
39.088
30.289
X : parts per Million : Carbon13
